# Pharmacology of Adenosine A_1_ Receptor Agonist in a Humanized Esterase Mouse Seizure Model Following Soman Intoxication

**DOI:** 10.1007/s12640-024-00717-z

**Published:** 2024-09-04

**Authors:** Tsung-Ming Shih, Crystal Munoz, Cindy Acon-Chen, Zora-Maya Keith

**Affiliations:** 1 Neuroscience Department, Medical Toxicology Research Division, U.S. Army Medical Research Institute of Chemical Defense, Aberdeen Proving Ground, MD 21010-5400 USA; 2 Present Address: University of Texas Southwestern Medical Center, 5323 Harry Hines Blvd, Dallas, TX 75390-9162 USA

**Keywords:** A_1_ adenosine receptor agonist, Anticonvulsant, Electroencephalographic activity, Genetically modified mouse strain, Neuroprotectant, Soman

## Abstract

Recently a novel genetically modified mouse strain with serum carboxylesterase knocked-out and the human acetylcholinesterase gene knocked-in (KIKO) was created to simulate human responses to nerve agent (NA) exposure and its standard medical treatment. A_1_ adenosine receptor (A_1_AR) agonist N-bicyclo-(2.2.1)-hept-2-yl-5'-chloro-5'-deoxyadenosine (ENBA) alone is a potent anticonvulsant and neuroprotectant (A/N) in both rat and KIKO mouse soman (GD) seizure models. In this study we utilized the KIKO mouse to evaluate further the basic pharmacologic A/N effects of ENBA as an adjunct to standard NA medical treatments (i.e., atropine sulfate, pralidoxime chloride [2-PAM], and midazolam). Male mice, implanted with cortical electroencephalographic (EEG) electrodes, were pretreated with asoxime (HI-6) and exposed to an epileptogenic dose of GD (33 µg/kg, s.c.) or saline (sham exposure) and then treated 15 min after seizure onset with ENBA at 15 mg/kg, i.p. (a minimum efficacy dose in suppressing NA-induced seizure) alone or as an adjunct to standard medical treatments. We collected EEG activity, seizure suppression outcomes, daily body temperature and weight, heart rate, toxic signs, neuropathology, and lethality data for up to 14 days. Without ENBA, death from NA exposure was 45%, while with ENBA, either alone or in combination with midazolam, the survival improved to 80% and 90%, respectively. Additionally, seizure was suppressed quickly and permanently, toxic signs, hypothermia, and bradycardia recovered by 48 h, and no neuropathology was evident. Our findings confirmed that ENBA is a potent A/N adjunct for delayed medical treatments of NA exposure.

**Supplementary Information:**

The online version contains supplementary material available at 10.1007/s12640-024-00717-z.

## Introduction

Organophosphorus nerve agents (NAs) such as soman (GD) and sarin (GB) are potent acetylcholinesterase (AChE) inhibitors. Inhibition of AChE can lead to a progression of toxic signs including hypersecretion, convulsions, seizures, respiratory distress, and death (Taylor 2011). Current standard medical treatments for NAs consist of combined therapy with an anticholinergic drug such as atropine sulfate, an oxime AChE reactivator such as pralidoxime chloride (2-PAM), and a benzodiazepine anticonvulsant such as diazepam or midazolam (MDZ) (Moore et al. 1995). Atropine antagonizes the effects of excess acetylcholine at postsynaptic muscarinic receptors, 2-PAM reactivates the activity of inhibited AChE, and the benzodiazepine controls convulsions and seizure activity. Current treatments reduce the toxicity of NA exposure and enhance the likelihood of survival, but when treatment is delayed, they are less likely to terminate sustained *status epilepticus* (SSE) causing subsequent neuropathology and behavioral deficits. Neuronal inhibitory drugs that block cholinergic receptors, increase the effect of γ-amino-butyric acid (GABA) receptors, or antagonize N-methyl-D-aspartic acid glutamatergic receptors are efficacious when administered quickly after NA exposure (Shih et al. 1991, 2003). However, after a period of SSE, the central nervous system (CNS) often becomes refractory to these treatments and seizures cannot be readily terminated (Shih and McDonough 1997; McDonough et al. 2010; Niquet et al. 2016). Therefore, a more complete understanding of the basic mechanisms driving neuroprotection are needed.

Toward that goal, our team has been investigating the adenosine signaling pathway (Cunha 2005) as a more effective inhibitory mechanism to terminate NA-induced seizure and neuropathology with promising results in rat models (Thomas et al. 2019). Our *in vivo* adenosine research initially utilized the A_1_ adenosine receptor (A_1_AR) agonist N6-cyclopentaladenosine (CPA) to prevent seizure and neuropathology after GB and GD exposure (Thomas and Shih 2014). While efficacious, CPA’s anti-seizure dose produced marked side effects such as sedation, hypothermia, bradycardia and hypotension (Thomas et al. 2019; Shih 2023). Consequently, we assessed two newer A_1_AR agonists, N-bicyclo-(2.2.1)-hept-2-yl-5'-chloro-5'-deoxyadenosine (ENBA) and 2-Chloro-N6-cyclopentyladenosine (CCPA), with greater affinity for the A_1_AR (Luongo et al. 2012; Thomas et al. 2019). They have proven to be efficacious against NA-induced seizure as well but with reduced side effects. When given one min after GD exposure, all these three A_1_AR agonists proved to be highly efficacious in preventing seizure occurrence.

While CPA’s and CCPA’s side effects persisted, ENBA displayed a faster recovery to consciousness, normal body temperatures, and baseline heart rates (Thomas et al. 2019; Loughery et al; 2021). In addition to preventing seizure onset, A_1_AR agonists can terminate ongoing SSE activity and limiting neuropathology when administered 15, 30, or 60 min after seizure onset. The most remarkable finding was that in GB-exposed rats, ENBA was able to limit the pathology to a score of 11.6 (out of 24 maximum) with a 60 min delay in treatment. Minimal or no neuropathology was observed with a 15- or 30-min treatment delay, indicating a strong neuroprotective capability for ENBA. A similar finding was observed in GD-exposed rat model for up to a 30 min treatment delay (Loughery et al. 2021).

The weakness of traditional rodent models of NA-induced SSE (Shih et al. 2003) is the presence of plasma carboxylesterase (CES) activity in rodents which may confound results, because humans and non-human primates do not express plasma CES (Li et al. 2005; Duysen et al. 2011). In 2011, Duysen and associates genetically created a plasma CES gene knock-out (Es1-/-) mouse strain that specifically lacks the activity of this enzyme in plasma. Expanding this approach, a novel humanized mouse strain was successfully generated (Cerasoli et al. 2019) in which plasma CES knock-out (KO) mice (Es1-/-) were cross-bred with a mouse strain expressing human AChE (knock-in; KI). The resulting human AChE KI/CES KO (or KIKO) mouse strain exhibited NA intoxication in a manner more like humans, as well as mimicking human therapeutic responses (DeBus et al. 2019; Reinhardt 2020).

**Fig. 1:**
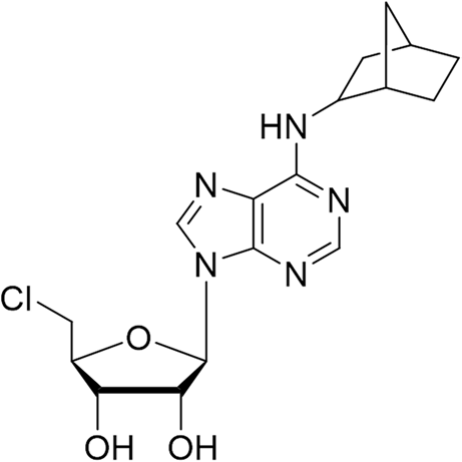
Chemical structure of ENBA (N-bicyclo-(2.2.1)-hept-2-yl-5'-chloro-5'-deoxyadenosine)

In this study, a recently developed KIKO mouse seizure model (Shih 2023) was used to evaluate the anticonvulsant and neuroprotective (A/N) effects of the A_1_AR agonist ENBA (see Fig. 1 for chemical structure) as adjunct to standard medical treatments following GD exposure at a delayed time point (15 min after SSE onset).

## Materials and Methods

### Subjects

Male KIKO (C57BL/6-Ces1^ctm1.1Loc^AChE^tm1.1Loc/J^) mice were obtained from the breeding colony at the U.S. Army Medical Research Institute of Chemical Defense (USAMRICD) (Aberdeen Proving Ground, MD) at 10—20 weeks of age (body weight: 25—35 g). They were housed in individual cages in temperature (21 ± 2 °C)- and humidity (50 ± 20%)-controlled quarters that were maintained on a 12-h light–dark cycle (with lights on at 06:00 AM) and received *ad libitum* access to food (Laboratory Rodent Diet Chow, LabDiet) and water except during experimental periods. Animals were acclimated for 3–10 days prior to surgery and experiments were conducted 7 days after surgery.

The experimental protocols were approved by the Institutional Animal Care and Use Committee at USAMRICD, and all procedures were conducted in accordance with the principles stated in the Guide for the Care and Use of Laboratory Animals, the Public Health Service Policy on Humane Care and Use of Laboratory Animals, and the Animal Welfare Act of 1966 (P.L. 89–544), as amended.

### Materials

Normal saline (0.9% NaCl) injection, USP, was purchased from Quality Biological (Gaithersburg, MD). Atropine methyl nitrate (AMN) and atropine sulfate (ATSO_4_) were purchased from Sigma-Aldrich (St. Louis, MO). Pralidoxime chloride (2-PAM) was purchased from Dishman Pharmaceuticals (Middlesex, NJ). Midazolam (MDZ) in solution was purchased from Hospira, Inc (Lake Forrest, IL). Asoxime chloride (HI-6; 1-[[2-[(E)-hydroxyiminomethyl]pyridin-1-ium-1-yl]methoxymethyl] pyridin-1-ium-4-carboxamide dichloride) was synthesized by Kalexyn Medicinal Chemistry (Kalamazoo, MI). Soman (GD, pinacolyl methylphosphonofluoridate) was obtained from the U.S. Army Combat Capabilities Development Command Chemical Biological Center (Aberdeen Proving Ground, MD). N-Bicyclo(2.2.1)-hept-2-yl-5'-chloro-5'-deoxyadenosine (ENBA) was purchased from R&D Systems (Minneapolis, MN). Model IPTT-300 Temperature transponders were purchased from Biomedic Data Systems Inc. (Seaford, DE). The MouseOX Plus Pulse Oximeter was purchased from STARR Life Science Corporation (Oakmont, PA). CED 1902 amplifiers and Spike2 software (Version 7) were purchased from Cambridge Electronic Design, Ltd. (Cambridge, UK). ENBA was prepared in multisol vehicle (48.5% distilled H_2_O, 40% propylene glycol, 10% ethanol, and 1.5% benzyl alcohol) on the day of treatment. Diluted preparations were sonicated until the drug was completely dissolved. GD, HI-6, 2-PAM, AMN, ATSO_4_ were diluted in normal saline. GD was injected subcutaneously (s.c.), and HI-6, 2-PAM, AMN, ATSO_4_, MDZ, and ENBA were injected intraperitoneally (i.p.). Injection volumes utilized, based on the drugs solubility in powder form or original solution concentration received from the pharmaceutical suppliers, were 2.2 ml/kg for GD and saline, 4.2 ml/kg for HI-6, 5.3 ml/kg for AMN, 5 ml/kg for 2-PAM and ATSO_4_, 0.7 ml/kg for MDZ, and 2.0 – 10.0 ml/kg for ENBA.

### Experimental Procedures

Approximately one week prior to experimentation, KIKO mice were surgically prepared with the subcutaneous implantation of a temperature transponder for recording body temperature and cortical wire electrodes for recording brain electroencephalographic (EEG) activity and detecting seizure onset and termination. All surgical procedures used in these experiments were conducted as previously described (Thomas et al. 2019; Loughery et al. 2021; Meads et al. 2021; Shih 2023). Briefly, animals were shaved (head and neck) in preparation for the implantation of the transponder and EEG electrodes while under isoflurane anesthesia (5% for less than or equal to 3 min) and oxygen mixture. Following anesthesia induction, an implantable electronic ID transponder (Biomedic Data Systems Inc., Seaford, DE) was inserted subcutaneously (s.c.) between the shoulder blades. Each animal was then mounted in a Kopf stereotaxic frame and three cortical stainless-steel screw electrodes were implanted in the skull: two were placed bilaterally ~ 3.0 mm lateral from the midline and equidistant between bregma and lambda; the third was placed on the posterior calvaria as the reference electrode. Stainless-steel wires attached the screws to a miniature connector plug. The screws, wires, and connector were then anchored to the skull with dental acrylic.

On the experiment day, mice were randomly assigned to various experimental groups and placed in individual chambers (dimensions in cm: 23 L × 31 W × 45 H). The cortical screw electrodes were connected to an amplifier and tethered via a rotating swivel for free movement. Their EEG activity was recorded using CED 1902 amplifiers and Spike2 software. Baseline brain EEG activity was collected for a minimum of 30 min. During this time, baseline physiological readings (such as heart rate and body temperature) and functional observational battery (FOB)-based neurobehavioral scores (such as righting reflex, startle reflex, motor activity, gait, and arousal) were recorded. However, our presentation focused on righting reflex, general state of movement, and toxic motor activity (Table 1), because ambulation (e.g., general state of movement) was the most representative measure of function or incapacitation for rodents under NA exposure and ENBA treatment (Loughery et al. 2021; Shih 2023). Heart rate was noninvasively monitored using the MouseOX Plus Pulse Oximeter and body temperature measured through the implanted temperature transponder.

**Table 1:** Functional Observational Battey (FOB) scoring rubric for toxic signs after exposure and treatments

Observation category	Sign score			
	0	1	2	3
Righting reflex	Normal (Immediate righting)	Impaired (< 1 s)	Impaired (> 1 s)	------------------------
General state of movement	Normal (Purposeful)	Uncoordinated (Voluntary Movement)	Impaired (Unresponsive > 1 s)	Prostrated (Loss of Tension)
Toxic motor activity (convulsion)	Normal (Absence of activity)	Fasciculation (Twitching, spontaneous)	Tremors (Localized hyperactivity)	Convulsion (Global)

The observational scoring for righting reflex (0 to 2) and general state of movement and toxic motor activity (0 to 3) were recorded just before and at 0, 4, 8, 15, 30, 45, and 60 min, and thereafter at 30-min intervals for 5 h after soman (GD) or saline exposure. Final score was taken at 24 h

**Fig. 2:**
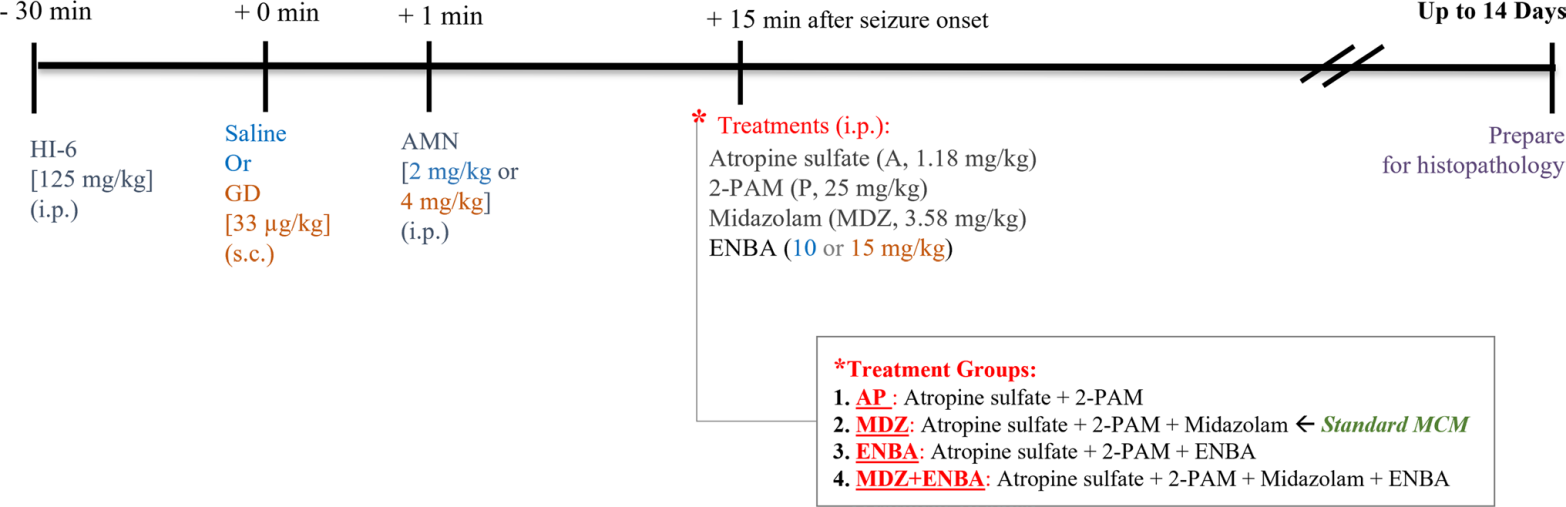
Experimental exposure and treatment paradigm of the KIKO mouse soman (GD) seizure model. KIKO mice were pretreated with HI-6 (125 mg/kg, i,p,) 30 min prior to challenge with a dose of saline (sham) or GD (33 µg/kg, s.c.) and treated one min later with atropine methyl nitrate (2 mg/kg for saline-exposed, 4 mg/kg for GD-exposed, i.p.). Animals were randomly assigned to one of the 4 treatment groups (as shown under [  ]). Treatment group #2 is designated as MDZ which is the standard medical countermeasure (MCM) for organophosphorus NA. Treatments were administrated i.p. at 15 min after GD-induced EEG seizure onset or relevant time for saline exposure groups. Brain EEG seizure activity was recorded for 24 h, and neuropathology scores were assessed at 14 days following saline or GD exposure

The experimental exposure and treatment paradigm is shown in Fig. 2, as previously described (Shih 1990, 2023). Mice were administered HI-6 (125 mg/kg, i.p.) at 30 min before exposure to GD or saline (sham exposure). One min later, animals received an i.p. injection of atropine methyl nitrate (AMN; 4 mg/kg for GD-exposed, 2 mg/kg for saline-exposed). A lower dose of AMN was used in saline-exposed animals to prevent choking due to the dehydrating effect of AMN. GD was chosen as the representative NA for the study because it’s the most challenging NA agent to treat due to rapid aging of its binding complex with AChE. Furthermore, a test compound found to be successful against GD intoxication was usually shown to be equally efficacious toward other NAs as well (McDonough et al. 1999, 2000; Shih et al. 2003). HI-6 and AMN, both of which are charged quaternary ammonium compounds and do not affect brain EEG seizure onset or severity, were given to ensure survival long enough for the appropriate measurement of any A/N activity of the tested compounds. Because of rapid aging of the GD-AChE complex, HI-6 was given in anticipation of the GD exposure. Additional doses of AMN (up to 8 mg/kg/mouse) were administered upon observation of peripheral symptoms (i.e., rhinorrhea, mucus secretions, salivation) to keep the air way open and clear. This is in line with our previous HI-6-pretreated GD seizure rat models (Loughery et al. 2021; Meads et al. 2021). We previously determined the minimum dose of GD (33 ug/kg, s.c.) that induces 100% EEG seizure activity in exposed KIKO mice while resulting in minimum lethality (< 50%) at 24 h and the minimum effective dose (MED) of ENBA (15 mg/kg, i.p.) that stops GD-induced SSE activity in 100% of animals when treated at 15 min after EEG seizure onset in this KIKO mouse seizure model (Shih 2023). Similarly, we found the MED for ENBA to induce EEG suppression (i.e., to isoelectric state) in the saline-exposed (non-EEG seizures) KIKO mice to be 10 mg/kg, i.p. (Shih 2023). It’s to be expected that with GD-induced seizure activity, a higher dose (15 mg/kg) of ENBA is required to counter the SSE after 15 min delay in treatment to produce same level of isoelectric activity in 100% of animals as 10 mg/kg does to saline-exposed animals (see Shih 2023). Each treatment was given at 15 min after GD-induced SSE or equivalent time point (e.g., 9 + 15 = 24 min) following saline exposure, since the average time for GD to induce SSE was 8.9 ± 0.9 min (Shih 2023). This delayed animal therapy model better mimics the time interval a NA-exposed human casualty might experience before receiving medical intervention(s) from a first responder in a mass civilian exposure scenario.

Visual determination of seizure onset and seizure termination required the consensus of two trained individuals following pre-determined criteria. Seizure onset was defined as rhythmic spiking of at least twice the amplitude of baseline recordings lasting at least 30 s. Seizure termination was defined as a) spike amplitude below 100 µV, b) spike frequency of at least 10 s apart, and c) absence of spiking for at least 5 min. Re-onset was defined as any instance of repetitive spikes or sharp waves exceeding an amplitude of twice that of baseline (McCarren et al. 2018).

Fifteen min after SSE onset, animals were treated i.p. with one of the following 4 treatment groups: atropine sulfate (ATSO_4_) + 2-PAM (AP), ATSO_4_ + 2-PAM + midazolam (MDZ), ATSO_4_ + 2-PAM + ENBA, and ATSO_4_ + 2-PAM + MDZ + ENBA. The ATSO_4_ + 2-PAM (called AP for short) treatment serves as control to compare the effects of 3 other therapies (all included AP) listed above: designated as MDZ, ENBA, and MDZ + ENBA treatments, respectively. The doses for the current medical treatments (doses are human equivalent scaled based on body surface for mice) were as follows: ATSO_4_ (1.18 mg/kg), 2-PAM (25 mg/kg), and MDZ (3.58 mg/kg). For ENBA the dose was 10 and 15 mg/kg for saline and GD exposure, respectively (Shih 2023).

Mice were left on EEG recording for 24 h after GD exposure to determine if/when GD-induced EEG seizure was terminated, and if/when seizure activity returned to determine the duration of anti-seizure action of ENBA and other treatments. The mouse’s heart rate, body temperature, and FOB responses were assessed continuously after saline- or GD-exposure at 4, 8, 15, 30, 45, and 60 min and, thereafter, at 60-min increments until the end of the initial experiment day (5 h post-exposure), and subsequently at 24 h, 7 day and day 14 following GD or saline exposure. Specifically, after exposure to GD, the cholinergic toxic syndromes involve loss of temperature regulation, reduced respiratory function, loss of meaningful physical activity (such as food-seeking), and muscular impairments due to seizure which all affect normal behavior necessary for recovery. To measure the toxic impact of exposure to GD on the quality of health or quality of life (QoL) over time, daily body weight and temperature checks and post-exposure supportive care were given to capture overall health status and recovery. Body weight and temperature served as continuous variable measures to assess the degree of toxic effect in all animals. These data sets were analyzed further to measure overall QoL of those alive at endpoint. Fourteen days after exposure, final physiological parameters, FOB measurement, and toxic signs were taken and animal’s EEG activity was monitored for a half hour before being deeply anesthetized, euthanized, and perfused.

Behavioral deficits and brain pathology do not always correlate. While ENBA demonstrated the potential to prevent or reduce neuropathology after GD exposure, comprehensive evaluation of neuroprotection requires functional behavioral assessments. During this experiment, we assessed ENBA’s neuroprotective efficacy by measuring changes in motor and memory function via a two-way active avoidance shuttle box test with these treatment groups in the KIKO mouse and related those outcomes to neuropathological analysis. The active avoidance shuttle box test has been described elsewhere (Meads et al. 2021). Each mouse underwent active avoidance shuttle box behavioral testing at 7 and 14 days after saline or GD exposure. At 14-day after exposure, animals were anesthetized, euthanized, and perfused to permit brain extraction for further histological analysis. The neuropathology and behavioral testing scores between treatment and exposure groups were compared. The active avoidance shuttle box test results were summarized and are discussed briefly in this report since they have just been published elsewhere (reported in Harkins et al. 2024). Any animal that exhibited loss of body weight or hypothermia after the initial exposure day was provided food, heat, and fluid support post-exposure. Any animal that was found to be in a moribund condition was removed from the study as a treatment failure and then euthanized. Mice assigned to a treatment group that did not demonstrate EEG seizure activity within two hours of GD exposure were excluded from the data analysis and animal count of their assigned groups and humanely euthanized via overdose of pentobarbital sodium as soon as possible after this 2-h window.

### Neuropathology

Fourteen days after saline or GD exposure and treatments and following final test measurements and body weight taken, the survivors were deeply anesthetized, euthanized, and perfused. Their brains were extracted and prepared for histology with H&E staining (Loughery et al. 2021; Shih 2023). Six vulnerable brain regions associated with severe neurological deficits after GD exposure (i.e., cerebral cortex, amygdala, piriform cortex, dorsal hippocampus, ventral hippocampus, and thalamus) were each evaluated by a trained pathologist who was unaware of treatment paradigm and scored using the established standard rubric for each brain region as described in detail elsewhere (McDonough et al. 1995; Loughery et al. 2021; Shih 2023): 0 = No lesion; 1 = Minimal (1 – 10%); 2 = Mild (11 – 25%); 3 = Moderate (26 – 45%); and 4 = Severe (> 45%). The maximum neuropathology score for each individual brain region was 4. A total neuropathology score was then calculated and summed for each mouse from 0 (normal) to 24 (the most severe brain damage). Brain pathology for each treatment group was compared among those obtained in saline-exposed or GD-exposed animals.

### Data Analysis

GraphPad Prism 9 and SPSS 22 was utilized for inferential analysis. Descriptive analyses of the physiological (i.e., body temperature, heart rate, and weight) and neurobehavioral variables (i.e., functional operational battery and toxic scoring) were calculated for each animal within a treatment group and used to indicate significant skew or deviation from normality for the group, then compared across groups with the appropriate method. Grubb’s or ROUT outlier test was utilized on baseline data before group comparisons were made. Due to anticipated death in GD-exposed groups and the lethality-dependent fluctuations in statistical power between comparisons, the number of animals per GD-exposed group was larger than saline-exposed. Data were evaluated based on established criteria. Due to the nature of our repeated-measures studies, in which animals are observed repeatedly over time under different conditions (treatments and exposure challenges), we divided groups by exposure condition for analysis. This also accounted for the difference in controls and in the distribution of the data. Statistical analyses were first performed separately for saline- and GD-exposed groups, with each individual group containing at least *n* = 4 at multiple time-points in each analysis. To compare across exposures, a Mann–Whitney test was conducted to detect significant main effects. Some analyses included timepoints that were deemed relevant based on graphical data (such as day 1, 7, and 14). For all statistical evaluations, *p* < 0.05 was considered significant.

#### ENBA Duration of Action

**Fig. 3:**
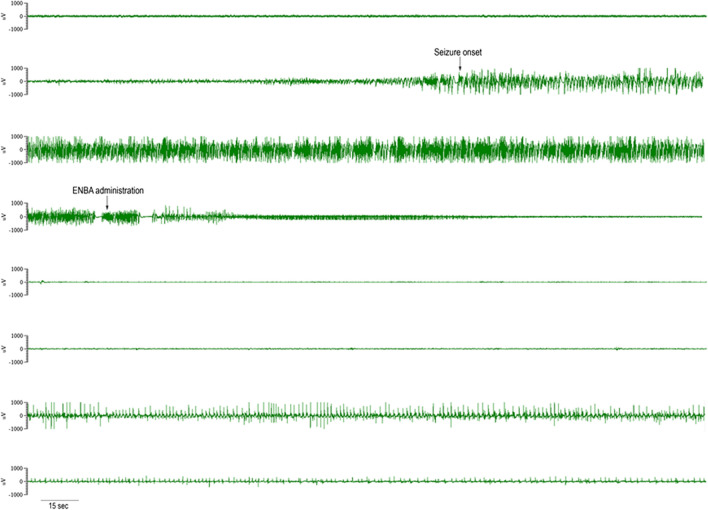
Example of EEG tracings of soman (GD)-induced *status epileptics* and ENBA or midazolam (MDZ) treatments. KIKO mice were treated with HI-6 (125 mg/kg, i,p,) 30 min prior to challenge with a dose of GD (33 µg/kg, s.c.) and treated one min later with atropine methyl nitrate (AMN; 4 mg/kg, i.p.). Fifteen min after GD-induced EEG seizure onset, animals were treated with ENBA (i.e., AP + ENBA) or MDZ (i.e., AP + MDZ). The top tracing is the baseline brain EEG activity; second tracing shows the onset of *status epilepticus*; third tracing displays the sustained *status epilepticus* (SSE); the 4th tracing shows treatment with ENBA and the termination of SSE into an isoelectric state; 5th and 6th tracings display the continuing isoelectric activity at 5 h and 24 h, respectively, after ENBA treatment; while 7th and 8th tracings show EEG seizure activities were unabated at 5 h and 24 h, respectively, after MDZ treatment

The effect of ENBA administration on EEG was assessed for duration based on exposure. For the saline-exposed control group, the time to the awake state was defined as median time between end of the isoelectric state and the onset of awake brain wave. The difference between ENBA’s duration of action in saline-exposed mice was not different after ENBA administration to 24 h. To assess ENBA’s duration of seizure suppression action in GD-exposed groups, the EEG was assessed from ENBA administration to 24 h after (Fig. 3). Time to seizure suppression was defined as the latency for the EEG to become comparable to baseline EEG, which was rapidly proceeded by the onset of the isoelectric state. Within 24 h after treatment, EEG was observed for seizure return which defined the end of ENBA’s duration of seizure suppression action; however, a seizure return response was not observed.

#### EEG Gamma Frequency Analysis

EEG recordings from the initial experiment day were analyzed using QuoStatus custom statistical software (Python-based software, University of Utah) to calculate gamma band power and spike frequency (Lehmkuhle et al. 2009; McCarren et al. 2018). QuoStatus was used to assess changes in spike and power; this software has the capability to evaluate various frequency bands individually, but our analysis was confined to the gamma band within the 5-h post-exposure timeframe to determine seizure onset and termination (when EEG was isoelectric). Each recording was subsequently reviewed by a trained investigator for up to 24 h to monitor the return of seizure activity or the end of sedation. Follow-up EEGs on days 7 and 14 post-exposure were also reviewed to observe changes relative to the baseline. Although delta power is critical to healthy EEG activity, we did not include an analysis of this or other bands in this study due to the pathological conditions of the NA-exposed population. Additionally, given the sedative nature of our treatment compounds, like MDZ and ENBA, we would expect to see a lot of artificial noise in the delta band. For these reasons, we chose to isolate the gamma band, as it is the most reliable measure of seizure initiation and termination for our exposure and treatment paradigms.

The analysis was performed for all treatment groups. Data were reported as mean changes from baseline ± SD for each group. Mean changes in gamma band power (20–60 Hz) and spike frequency from baseline at 15-min intervals were compared between experimental groups and their respective controls using a two-way repeated measures ANOVA followed by Dunnett’s test if treatment effect was significant (*p* < 0.05). Values for change in spike rate and gamma power relative to baseline for each treatment group of interest were compared at the selected time points: at 30 min before SSE onset, at treatment time (15 min after SSE), and at every h for 1–4 h after SSE onset. We performed unpaired t-tests when two groups were compared or one-way ANOVAs when three or more groups were compared. For significant ANOVAs, Tukey’s comparison was used to evaluate differences between each group.

#### Lethality

Lethality was defined as a binomial event, with 'yes or no' outcome and the time of event was recorded for every animal. The probability of time-dependent survival was tested using the Kaplan–Meier survival analysis and log rank test. Time to death by GD across treatment groups for 14 days was input by exact time within 24 h and approximated for the following days if captured after the event occurred (e.g., an overnight death leading to day 13 = 12.5). The mean survival time was not significantly different between the curves. When reviewing the curves, the distribution reflects death events clustered at 1 timepoint: 24 h.

#### Daily Assessments for Health, GD Toxicity Index, and Quality of Life (QoL) after Treatment for up to Day 14

Temperature and body weight were selected as biological pre-cursers indicative of illness over time to serve as measures of toxicity after exposure to GD. With body weight and temperature as continuous variable measures, the degree of effect was indexed in all animals across day 1–14 and given rank relative to severity, regardless of group membership (treatment).

1. Quality of daily health across recovery for up to 14 days was assessed through temperature and body weight for all experimental animals regardless of saline or GD exposure. Every day, each individual animal had temperature and weight indexed for degree of effect such that measurements were given a rank relative to severity. A normal body temperature range was defined between 36–38 °C, below normal was between 35.0–35.9 °C, and hazardous (approaching hypothermic) at 34.9 °C or below. Body weight was normalized for each animal as follows: (recorded weight/baseline weight) *100. A normal range for the % weight of baseline was 95.0–100%, below normal was between 94.0–94.9%, and hazardous was at or below 93.9% of baseline. The hazard index for temperature and weight measurements were set using Microsoft Excel ‘Conditional Formatting Rules’ for immediate feedback on animal condition (Supplemental Table 20).
2. For the GD-exposed animals, daily observations across time (or days 1–14 post-treatment) were ranked within the measure for degree of toxicity, then summarized for the number of observations indicative of toxic effect out of all observations, by treatment group (Table 4). The level of toxicity after GD exposure was indexed to measure toxic effect and orders the level of toxicity as such: absence of toxic effect = 0%, negligeable toxic effect = 25%, median toxic effect = 50%, high impact toxic effect = 75%, and gross toxic effect = 100%. If death occurred, then animal automatically placed in the 100% quartile. The degree and rank by measure for all 40 GD-exposed animals was reviewed across time (day 1–14). The distance between rank distribution for the groups indicates unmitigated toxicity after treatment. If death occurred, then animal was automatically placed in the 100% quartile. The saline controls had temperature and body weight trends examined to determine the effects of the pharmacology against the cholinergic syndrome and to, thus, determine absence of GD toxicity (non-toxic level). The toxic index, therefore, summarized the results of the experimental (GD-exposure) situation and ability to recover.
3. These data sets were analyzed further to quantify Quality of life (QoL) of those alive at the endpoint of the study after exposure to GD, (Table 5). Lethality was recorded daily, and of those who survived to the end of the study, or 14 day, QoL by treatment was assessed to measure whether there was any effect on the impact of GD exposure on quality of health and therefore, likelihood of survival beyond the study. The interaction of the temperature rank, weight rank, and time determined overall average rank. The data scattering, average rank and distance from the mean over time then served as the measure of overall toxicity, such that if an animal had a high rank (or high degree of toxic effect) over a period, this was then turned into percent of total time spent with high effect. Finally, group membership was re-introduced to determine the average rank of toxicity and distribution within each group.

#### Body Temperature, Heart Rate and Weight

Prior to each analysis of variance (ANOVA) test, we verified normal distribution using a residuals QQ plot and validated equality of variance by Brown-Forsythe test, *p* > 0.05. In general, the following tests were used to detect significant main effects for normal distributed data: ANOVA equivalent to the general linear model (GLM), and a linear mixed effect model (LMEM) using restricted maximum likelihood (REML) calculations with the Geisser-Greenhouse correction in Prism. These tests were chosen based on the repeated-measures method used in collecting the data (animals were observed repeatedly over time). For analysis purposes, pair-wise comparisons were made by the treatment group to evaluate simple effects within animals, and then pair-wise comparisons were made across treatment groups to evaluate treatment effects. For temperature and heart rate comparisons, a LMEM followed by a Dunn’s multiple comparison test to compare within groups and across group was selected post-hoc to control the family-wise error rate and identify which treatment had significant outcomes. For weight comparisons, a LMEM followed by a Dunn’s multiple comparison test to compare within groups was selected while a Tukey’s multiple comparison test was conducted for across group comparisons post-hoc to control the family-wise error rate and identify which treatment had significant outcomes. Absent values in one or multiple measures are missing for completely indiscriminate reasons (e.g., an observation at a time-point was missed due to accidental error, value for animal could not be determined due to technical issue, or due to animal death) unless noted. These incidences are mentioned below and were considered when conducting each analysis; any variation otherwise to outcomes is noted alongside the data. A “treatment group” was defined as the combination of exposure (saline or GD) and treatment (AP, AP + MDZ, AP + ENBA, or AP + MDZ + ENBA).

## Results

### Effects on EEG Seizure Activity and Subsequent Neuropathology (Figs. 3 and 4; Table 2, Supplemental Tables 7–8)

The goal of this study was to evaluate if A_1_AR stimulation is the mechanistic driver of the A/N effect of ENBA. Therefore, our focus was the pharmacologic actions of ENBA on prevention and termination of seizures, mitigation of brain pathology, and enhancement of survival.

**Fig. 4:**
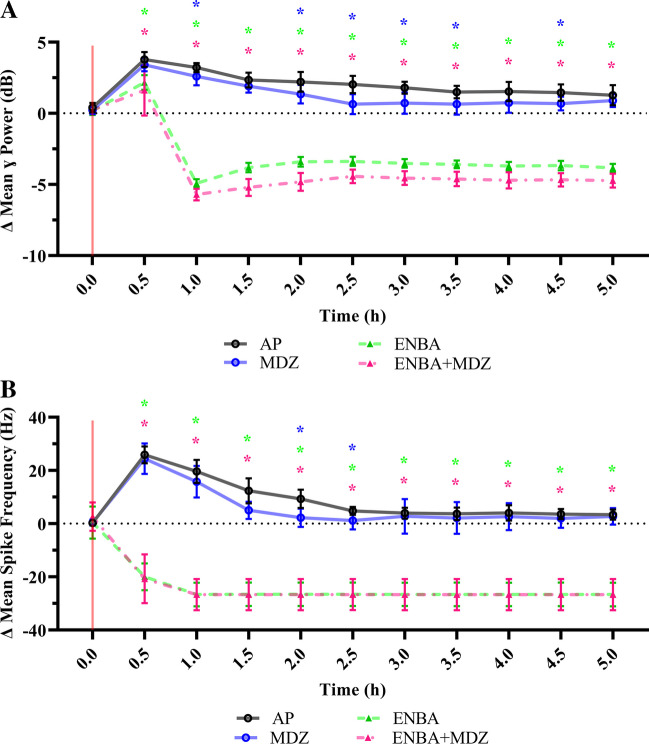
EEG gamma power and spike frequency analysis following soman (GD) exposure and treatments. Recording was analyzed using QuoStatus (Python-based software, University of Utah) to calculate gamma band power (4A) and spike frequency (4B). KIKO mice were pretreated with HI-6 (125 mg/kg, i,p,) 30 min prior to challenge with a dose of GD (33 µg/kg, s.c) and treated one min later with atropine methyl nitrate (4 mg/kg, i.p.). Animals were randomly assigned to one of the 4 treatment groups: AP (atropine sulfate + 2-PAM), MDZ (AP + midazolam), ENBA (AP + ENBA), or MDZ + ENBA (AP + midazolam + ENBA). Treatments were administrated i.p. at 15 min after GD-induced EEG seizure onset. EEG activity was analyzed for the first 5 h following GD exposure using REMLS/Mixed effect model and Dunn’s multiple comparisons. (*) indicates significantly different (*p* ≤ 0.05) from AP group. Significant reductions in both power (4A) and spike frequency (4B) were prominent within 15 min of treatment ([AP vs. ENBA: Power: *p* < 0.001 and Spike Frequency: *p* < 0.001] and [AP vs. ENBA + MDZ: Power: *p* = 0.016 and Spike Frequency: *p* < 0.001]). Robust control of EEG spiking in the gamma power band was maintained only by ENBA-included treatment groups throughout the rest of the recording, confirming no anti-seizure effects from the AP and AP + MDZ treatments

**Table 2:**
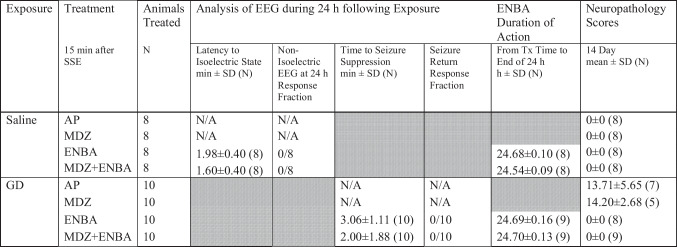
Efficacy of medical treatments with and without ENBA following saline and soman (GD) exposure

Comparing ENBA administration across saline and soman (GD) exposures at respective minimum effective dose. KIKO mice were pretreated with HI-6 (125 mg/kg, i,p,) 30 min prior to challenge with a dose of GD (33 µg/kg, s.c.) or saline and treated one min later with atropine methyl nitrate (2 mg/kg, i.p. for saline exposure and 4 mg/kg, i.p. for GD exposure). Animals were randomly assigned to one of the 4 treatment groups: AP (atropine sulfate + 2-PAM served as control), MDZ (AP + MDZ = standard medical countermeasure), ENBA (AP + ENBA), and MDZ + ENBA (AP + MDZ + ENBA). Treatments were administrated i.p. at 15 min after GD-induced EEG seizure onset and relevant time for saline exposure groups. Brain EEG activity was recorded for 24 h. Neuropathology scores were assessed at 14 days following exposure, if available (max score possible of 24.0). ENBA in saline-exposed mice produced > 24-h sedation and isoelectric activity on the EEG. All 40 GD-exposed animals seized approximately 3.92 ± 2.8 min after GD. When ENBA was involved in the treatment regimen (alone or with MDZ), SSE was suppressed quickly and did not return. GD-exposed and ENBA-treated mice were absent of neuropathology while mice in the GD-exposed AP- or MDZ-treated groups seized on EEG for the 24 h and had resulted in moderate neuropathology (scores 13.71 – 14.20 out of 24.0 maximum). SSE = sustained *status epilepticus*; Tx = treatment

A typical example of EEG tracings of GD-induced SSE and ENBA or MDZ treatment in KIKO mice is shown in Fig. 3. Fifteen min after GD-induced SSE onset, animals were treated with either  ENBA or MDZ (i.e., AP + ENBA or AP + MDZ treatment group, respectively). The top tracing is the baseline brain EEG activity; second tracing shows the onset of *status epilepticus*; third tracing displays the SSE; the 4th shows treatment with ENBA and termination of SSE with the tracing gradually turning into an isoelectric state; the 5th and 6th tracings display the continuing isoelectric activity at 5 h and 24 h, respectively, after ENBA treatment. The 7th and 8th tracings show EEG seizure activities were unabated at 5 h and 24 h, respectively, after MDZ (i.e., AP + MDZ) treatment, which are similar to previous observations with rats and guinea pigs; the seizure activities were still visible at 24–48 h after GD exposure in the survivors, even though the frequency and spike amplitude were much reduced (McDonough and Shih 1993, 1997).

Table 2 shows the effects of treatment groups on latency to isoelectric state, response fraction of non-isoelectric EEG at 24 h, time to seizure suppression, response fraction of seizure return, ENBA’s duration of action, and neuropathology scores in survivors at 14 days. In animals subjected to saline control exposure, groups of mice treated with ENBA and MDZ + ENBA produced a very rapid suppression of normal EEG activity to an isoelectric state within minutes with an average of 1.98 ± 0.40 and 1.60 ± 0.40 min, respectively. Furthermore, this effect lasted for 24 h after treatment; no single animal presented with normal baseline-like EEG activity within 24 h. The latency to isoelectric state and ENBA’s duration of action for all 16 saline-exposed ENBA-treated mice were within seconds of each other demonstrating ENBA at 10 mg/kg was having the same impact on EEG, with and without MDZ. On the other hand, for the groups treated with AP and AP + MDZ (= standard MCM), their EEG activity did not deviate much from baseline and were not sedated throughout the 24 h period after treatment. None of these 4 treatment groups following saline control exposure caused any visible neuropathology and all animals survived 14 days after saline exposure and treatments.

In mice exposed to GD, treatment with ENBA and MDZ + ENBA at 15 min after SSE onset induced a very rapid suppression of ongoing SSE activity (3.06 ± 1.11 and 2.00 ± 1.88 min, respectively; Table 2). Furthermore, this promoted an isoelectric state almost immediately after and lasted for the 24 h after treatment. With and without MDZ, all 20 mice presented without seizure reoccurrence at 24 h, demonstrating no difference on EEG between these two ENBA-treated groups in terms of time to seizure suppression and ENBA’s duration of action. Furthermore, neither of these two ENBA-included treatment groups displayed any visible neuropathology at 14 days after GD exposure and treatments, even though they endured at least 15 min of continuing SSE seizure activity before treatment was given. On the other hand, for the groups treated with AP and AP + MDZ, their ongoing SSE seizure activity continued for more than 24 h and subsequently exhibited severe neuropathology (~13 to 14 out of 24 maximum scores) in survivors at 14 days.

It is interesting to note that the effects of ENBA, in either ENBA alone or MDZ + ENBA treatment groups, on the latency to isoelectric state and seizure suppression under saline and GD exposure, respectively, and on the duration of EEG suppression under both saline and GD exposures are identical. This indicates that the action of ENBA is not influenced by the state of brain activities, whether it’s normal (saline exposure) or under severe ongoing SSE activity (GD exposure). Furthermore, under 15 min of severe and intense CNS seizure activity after GD intoxication, treatment with an MED of ENBA relieved neurons from pathological consequences observed at 14 days after such exposure.

Figure 4 displays the EEG gamma power band (Fig. 4A) and spike frequency (Fig. 4B) analysis during the first 5 h following GD exposure and treatments on the experimental day. The EEG reflects changes in seizure intensity after GD and treatment. Recording was analyzed using QuoStatus (Python-based software, University of Utah) to calculate gamma band power and spike frequency. All groups were not significantly different at baseline for power (range = 0.18 ± 0.30 to 0.41 ± 0.33 dB; AP vs. MDZ: DF = 17.7, *P* = 0.469; AP vs. ENBA: DF = 17, *P* = 0.329; AP vs. MDZ + ENBA: DF = 16.9, *P* = 0.455) and spike frequency measurements (range = 2.62 ± 5.35 to 0.25 ± 0.59 Hz; AP vs. MDZ: DF = 14.1, *P* = 0.977; AP vs. ENBA: DF = 8.15, *P* =  > 0.999; AP vs. MDZ + ENBA: DF = 8.2, *P* = 0.451). Within the AP group, power ranged from 3.79 ± 0.52 to 1.57 ± 0.70 dB and spike frequency ranged from 25.87 ± 3.18 to 3.40 ± 1.91 Hz for the 5 h after GD exposure. Power was significantly above baseline for all 5 h (1 h: DF = 9, *P* =  < 0.001; 5 h: DF = 9, *P* = 0.049). Spike frequency was also significantly above baseline for all 5 h (1 h: DF = 8, *P* =  < 0.001; 5 h: DF = 8, *P* = 0.005) AP failed to reduce the SSE activity on EEG after GD. Within the MDZ group, power ranged from 3.40 ± 0.45 to 0.89 ± 0.45 dB and spike frequency ranged from 24.42 ± 5.74 to 1.18 ± 3.33 Hz throughout the 5 h after GD. MDZ power and spike frequency were significantly above baseline for 1.5 h after GD (power at 1.5 h: DF = 9, *P* =  < 0.001; spike frequency at 1.5 h: DF = 9, *P* = 0.008) then began ramping down afterwards. Within the ENBA group, mean power reduced rapidly from 2.17 ± 0.51 at 15 min of treatment to -4.91 ± 0.28 dB within 45 min after treatment, while change in mean spike frequency dropped to -20.00 ± 5.05 Hz within 15 min of treatment. This overall reduction effect on EEG activity sustained throughout the 5 h after GD. Power was significantly reduced compared to baseline beginning at 0.5 h after GD (− 4.91 ± 0.28 dB; DF = 8, *P* =  < 0.001). Spike frequency was significantly below baseline 0.5 h after GD (− 20.00 ± 5.05 Hz; DF = 8, *P* =  < 0.001) and maintained below this throughout the rest of the initial experimental day, (5 h: − 26.65 ± 4.42 Hz). Within the MDZ + ENBA group, the impact on EEG was similar in trend to ENBA alone group. The mean power reduced from 1.62 ± 1.78 at 15 min of treatment to -5.71 ± 0.28 dB within 45 min after treatment which was significantly reduced compared to baseline (or 1 h after GD: − 5.71 ± 0.41 dB, DF = 8, *P* =  < 0.001). The mean spike frequency was significantly below baseline at 15 min after treatment (or 0.5 h after GD: − 20.75 ± 9.15 Hz, DF = 8, *P* =  < 0.001) and reduced further to -26.70 ± 5.85 Hz from 1 h and sustained throughout the 5 h after GD. Within 15 min of treatment, significant differences in change for ENBA and MDZ + ENBA against the control (AP group) were evident for both power (AP vs. ENBA: DF = 16.9, *P* =  < 0.001; AP vs. MDZ + ENBA: DF = 9.25, *P* = 0.016; Fig. 4A) and spike frequency (AP vs. ENBA: DF = 13.2, *P* =  < 0.001; AP vs. MDZ + ENBA: DF = 9.74, *P* =  < 0.001; Fig. 4B).

Overall, the MDZ treatment intermittently reduced power significantly compared to the control group (AP vs. MDZ at 1 h: DF = 13.4, *P* = 0.037; 2 h: DF = 17.9, *P* = 0.027, and 4.5 h: DF = 17.5, *P* = 0.013). The effect on spike frequency with MDZ was similar with a short reduction lasting approximately 1 h when compared to the AP control group (AP vs. MDZ at 1.5 h: DF = 16, *P* = 0.003; and 2.5 h: DF = 12.5, *P* = 0.022). However, only the ENBA and MDZ + ENBA treatments resulted in a lasting reduction in mean power throughout the 5 h (AP vs. ENBA at 5 h: DF = 11.9, *P* =  < 0.001; AP vs. MDZ + ENBA at 5 h: DF = 16, *P* =  < 0.001). Changes in the mean spike frequency were also lasting throughout the 5 h (AP vs. ENBA at 5 h: DF = 10.7, *P* =  < 0.001; AP vs. MDZ + ENBA at 5 h: DF = 9.54, *P* =  < 0.001). The ENBA alone or in combination to MDZ, effectively controlled the SSE-dependent rise in power and spike frequency compared to AP.

Neuropathological damage was assessed by a trained veterinary pathologist who was unaware of treatment paradigm and scores determined for each of the six brain regions (amygdala, cerebral cortex, piriform cortex, thalamus, dorsal hippocampus, and ventral hippocampus) by the percentage of degeneration and necrosis of neurons in each region with a maximum total severity score of 24 (McDonough et al. 1995; Loughery et al. 2021; Shih 2023). Other neuropathologic changes such as neuropil vacuolation and gliosis were noted in the GD-exposed mice but not used to determine the damage severity. Generally, animals with neuropathological damage in one region had damage in all regions, though the hippocampus was spared in some instances. The degree of damage across regions varied. For all of the survivors at 24 h, no brain pathology occurred when their seizure activity was terminated by the treatment of ENBA (Table 2). For example, ENBA treatment at 15 min after SSE onset showed a total protection in neuropathology score (0.0 ± 0.0) compared to AP- and AP + MDZ-treated groups (13.71 ± 5.65 [7] and 14.20 ± 2.68 [5], respectively). In the latter two treatment groups, brain sections have multifocally extensive neuronal necrosis and degeneration with neuropil vacuolation, while in the two ENBA-included groups brain sections have undamaged neurons with multifocal areas of ‘dark neurons’ (a handling artifact). The detailed examples of H&E-stained histology images in 6 brain regions of KIKO mouse induced by GD exposure and protected by treatment with ENBA alone have been published elsewhere (see Shih 2023 for details).

### GD Toxicity Effects, Lethality and Quality-of-Life. (Tables 2, 3, 4, and 5, Supplemental Table 20)

For saline-exposed groups, there was no mortality at 24 h following any of the 4 treatments; thus, the KIKO mouse is able to tolerate doses of ENBA and MDZ + ENBA, even though they produced a very rapid suppression of normal EEG activity to an isoelectric state within min and the effect lasted for 24 h. We had observed earlier, in our MED dose determination study, that ENBA up to 45 mg/kg in KIKO mouse did not cause mortality in 24-h (Shih 2023).

**Table 3:** Quality of health after soman (GD) exposure and treatment

Quality of health after GD across 14 days				
Treatment	% observations indicative of measure (*n*/total # of observations for measure)			
	Poor temperature	Distribution	Poor weight	Distribution
AP	8% (5/65)	Throughout day 14 days	31% (20/65)	Throughout day 1–10
MDZ	57% (37/65)	Throughout 14 days	94% (61/65)	Throughout 14 days
ENBA	12% (10/81)	Primarily on day 1	11% (9/81)	Across days 1–2
MDZ + ENBA	11% (9/85)	Primarily on day 1	11% (11/85)	Across days 2–3

KIKO mice were pretreated with HI-6 (125 mg/kg, i,p,) 30 min prior to challenge with a dose of soman (GD, 33 µg/kg, s.c.) and treated one min later with atropine methyl nitrate (4 mg/kg, i.p.). Animals were randomly assigned to one of the 4 treatment groups: AP (atropine sulfate + 2-PAM served as control), MDZ (AP + MDZ = standard medical countermeasure), ENBA (AP + ENBA), and MDZ + ENBA (AP + MDZ + ENBA). Treatments were administrated i.p. at 15 min after GD-induced EEG seizure onset. Lethality was recorded daily, and survivors’ quality of health was assessed daily. Quality of health was assessed using plots of daily assessment data (temperature and body weight), which were used to calculate the variability in severity across time. A poor temperature observation was defined as 35 °C or below and a poor body weight observation was defined as a change in weight from baseline of -7% or greater. ENBA-treated groups regained ability to self-regulate body temperature and maintain weight such that groups given ENBA (with and without MDZ) were all self-sustainable by study endpoint. The AP and MDZ groups had a higher likelihood of requiring supportive care throughout daily observations and presenting poor quality of health at 14 days, regardless of supplemental support

**Table 4:**
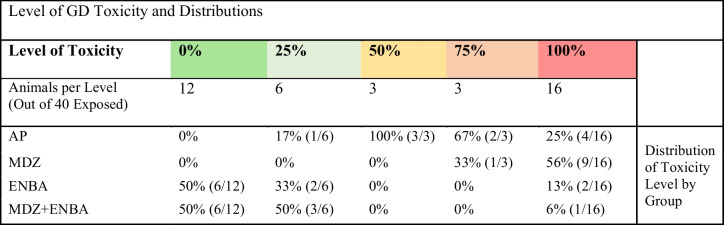
Toxicity index after soman (GD) exposure

KIKO mice were pretreated with HI-6 (125 mg/kg, i,p,) 30 min prior to challenge with a dose of soman (GD, 33 µg/kg, s.c.) and treated one min later with atropine methyl nitrate (4 mg/kg, i.p.). Animals were randomly assigned to one of the 4 treatment groups: AP (atropine sulfate + 2-PAM served as control), MDZ (AP + MDZ = standard medical countermeasure), ENBA (AP + ENBA), and MDZ + ENBA (AP + MDZ + ENBA). Treatments were administrated i.p. at 15 min after GD-induced EEG seizure onset. Lethality was recorded daily, and survivors’ quality of health was assessed daily. Quality of health was further assessed for the index of toxicity across time. The level of toxicity after GD exposure was indexed across all 40 GD-exposed animals to measure toxic effect and orders the level of toxicity as such; absence of toxic effect = 0%, negligeable toxic effect = 25%, median toxic effect = 50%, high impact toxic effect = 75%, and gross toxic effect = 100%. If death occurred, then animal automatically placed in the 100% quartile. The distance between rank distribution for the AP and MDZ groups reflects high variability and unmitigated toxicity after treatment. The ENBA and MDZ + ENBA groups were primarily distributed across the lowest 2 levels (0–25%), reflecting the lowest toxicity with low variability

**Table 5:**
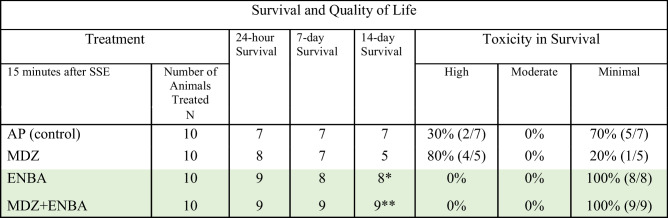
Quality of life at 14 days after soman exposure and treatment

KIKO mice were pretreated with HI-6 (125 mg/kg, i,p,) 30 min prior to challenge with a dose of soman (GD, 33 µg/kg, s.c.) and treated one min later with atropine methyl nitrate (4 mg/kg, i.p.). Animals were randomly assigned to one of the 4 treatment groups: AP (atropine sulfate + 2-PAM served as control), MDZ (AP + MDZ = standard medical countermeasure), ENBA (AP + ENBA), and MDZ + ENBA (AP + MDZ + ENBA). Treatments were administrated i.p. at 15 min after soman-induced EEG seizure onset. Lethality was recorded daily, and survivors’ quality of life was assessed at day 14 following GD exposure and treatment. ENBA increased survival and reduced the impact of exposure on quality of health. When ENBA was involved in the treatment regimen (alone or in combination with MDZ), group survival increased (to 80%* and 90%**) and of those that survived, all presented with the lowest level of toxicity. Additionally, groups given ENBA (with and without MDZ) were all self-sustainable and did not require extra post-exposure food, heat, and fluid support at study endpoint. The AP and MDZ groups had a higher likelihood of presenting with high levels of toxicity at 14 days, even with post-exposure supplemental support. ENBA overall mitigated the sustained high toxicity after soman exposure (ENBA & MDZ + ENBA = 0% vs. AP control = 30%)

For GD exposed groups, after AP, AP + MDZ, AP + ENBA and AP + MDZ + ENBA treatments (10 mice per group), the lethality at 24 h was 3, 2, 1, and 1, respectively, and at 14 days progressed to a final total of 3, 5, 2, and 1, respectively (Table 2). A probability assessment on time and survival determined no difference in mean time of survival between the treatment curves. Death events were clustered around 24 h. Additionally, animals who were dependent on supportive post-exposure care due to a plateau in recovery were observed and further noted as survival with abnormality. To assess the interaction of survival event with abnormalities (e.g., poor health), a measure of effects associated with GD toxicity was done. Specifically, a plot of daily assessments (temperature and body weight) was used to calculate the variability in severity across time and to capture the index of toxic effect across all treatments with higher confidence, when compared to a probability assessment of binomial event outcomes.

The quality of health was summarized for temperature and body weight by counting total observations and identifying the sum of poor observations (indicative of poor health) and expressed as ratios for each treatment group (Table 3). A temperature at or below 35 °C and -7% weight loss from baseline or greater were thresholds indicative of poor health. For the AP (control) group, 8% of the temperature and 31% of the weight observations were below threshold, of those observations the scatter was throughout the 14 days after treatment. This reflects variable recovery after GD. The MDZ group had high percentages of poor health observations in both measures (temperature = 57% and weight = 94%) dispersed throughout the 14 days. On the other hand, ENBA and MDZ + ENBA had minimal poor observations in both measures (temperature = 11–12% and weight = 11%). The observations were clustered between day 1–2, which aligns to the recovery period of A_1_AR agonist-induced sedation and hypothermia.

The level of toxicity after GD exposure was indexed across all 40 GD-exposed animals (Table 4). This quantifies the toxic effect and orders the level of toxicity as such; absence of toxic effect = 0%, negligeable toxic effect = 25%, median toxic effect = 50%, high impact toxic effect = 75%, gross toxic effect = 100%. The distance between average rank per group measures the toxicity variability for each treatment group. Consequently, as toxic effect increased over time (remained unmitigated), the risk of lethality over time also increased. Across all GD-exposed animals, 12 of the 40 had an absence of toxicity after treatment (0%), 6 of the 40 had inconsequential effects (25%), 6 had median to high impact toxic effects (50–75%), and 16 of the 40 were grossly impacted (100%). Next, we reviewed the distribution within the levels of toxicity by treatment group membership. The AP group had mice distributed between the upper 4 levels of toxicity (25–100%). Of the 10 mice belonging to the AP group, 5 were at the 50–75% levels and 4 were at the 100% level. Even more severe, the MDZ group had mice distributed between the upper 2 levels of toxicity (75–100%). Of the 10 MDZ-treated mice, 1 was at 75% while 9 were at the 100% level. In contrast, the ENBA and MDZ + ENBA groups had mice distributed primarily between the lower 2 levels of toxicity (0–25%). The ENBA group had 8 mice at 0–25%, and 2 at 100% who died around 24 h. The MDZ + ENBA group was similar, with 9 mice at 0–25% and 1 at 100% who died early on.

At 14-days, the quality-of-life (QoL) measures indicated that the control group treated with AP had 2 out of 7 animals present with high levels of toxicity, and MDZ had 4 out of 5 animals display high levels of toxicity (Table 5). While none of the 8 or 9 survivors in either ENBA or MDZ + ENBA groups had animals in such a weak condition. For the latter two ENBA-treated groups, the surviving animals were all self-sustainable and did not require extra post-exposure food, heat, and fluid support at the end point of the study.

### Effects on Body Temperatures (Fig. 5; Supplemental Fig. 1; Supplemental Tables 9–11)

**Fig. 5:**
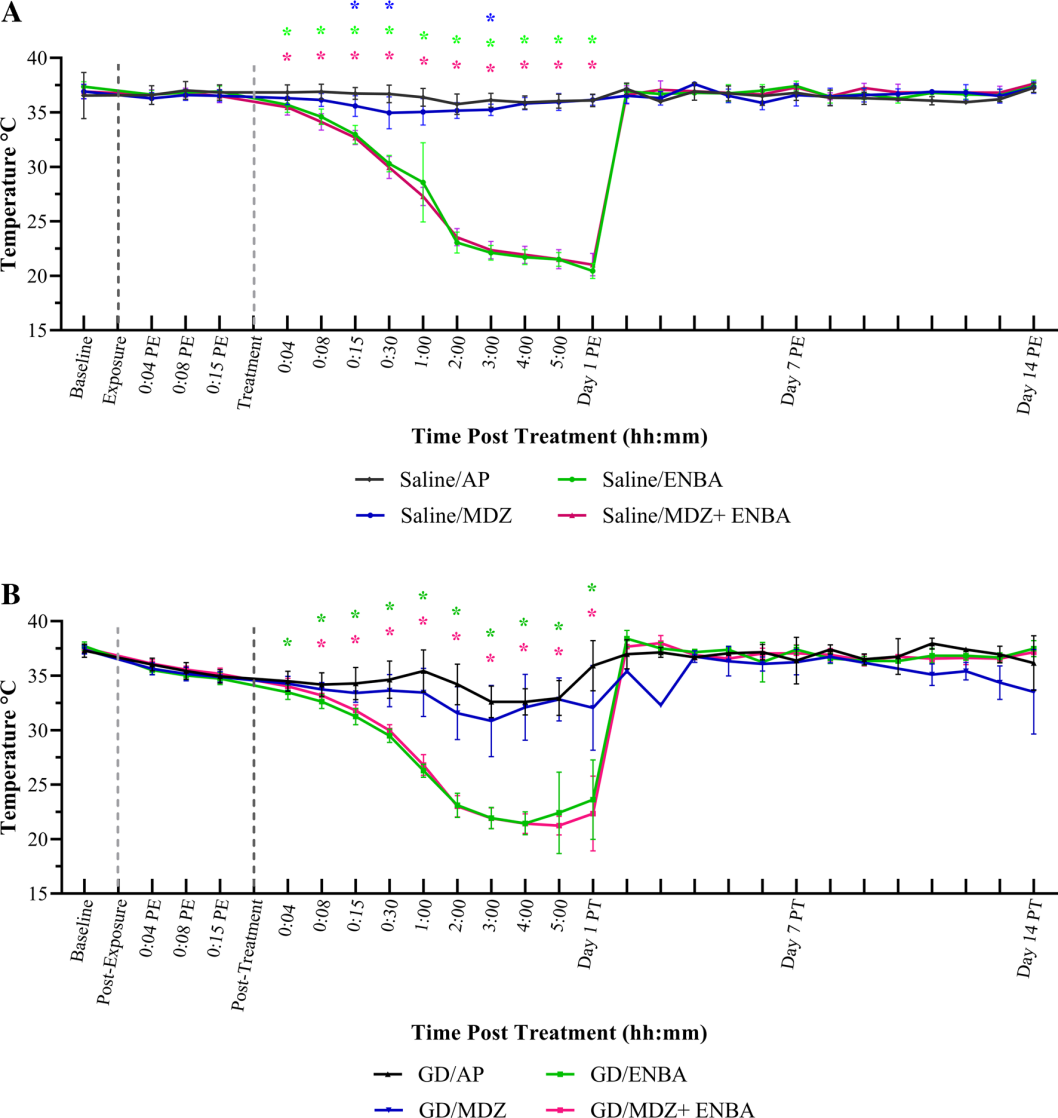
Fourteen-day body temperature recordings following saline (sham) or soman (GD) exposure and treatments. KIKO mice were treated with HI-6 (125 mg/kg, i,p,) 30 min prior to challenge with a dose of saline (5A) or GD (33 µg/kg, s.c.; 5B) and treated one min later with atropine methyl nitrate (2 mg/kg for saline-exposed, 4 mg/kg for GD-exposed, i.p.). Animals were randomly assigned to one of the 4 treatment groups: AP (atropine sulfate + 2-PAM), MDZ (AP + midazolam), ENBA (AP + ENBA), or MDZ + ENBA (AP + midazolam + ENBA). Treatments were administrated i.p. at 15 min after GD-induced EEG seizure onset or relevant time after saline exposure groups. Body temperature was recorded and statistically compared for the 5 h during experimental day, at 24 h, and on day 7 and 14 following exposure. Temperature was analyzed using REMLS/Mixed effect model and Dunn’s multiple comparisons for within and between group comparisons within each exposure. (*) indicates significantly different (*p* ≤ 0.05) from respective AP treatment group. ENBA treatments affect temperature profoundly within 4 min of treatment in saline-exposed mice (5A) compared to AP-treated mice ([AP vs. ENBA: *p* = 0.016] and [AP vs. ENBA + MDZ: *p* = 0.005]) up to 24 h after administration (AP vs. ENBA and ENBA + MDZ: *p* < 0.001); however, this was reversible with the addition of short-term thermal support at the 24-h mark. Similarly, ENBA profoundly impacted temperature in GD-exposed mice compared to AP-treated mice (5B) shortly after treatment ([at 4 min, AP vs. ENBA, *p* = 0.029] and [at 8 min, AP vs. MDZ + ENBA, *p* = 0.062] for up to 24 h after administration (AP vs. ENBA and ENBA + MDZ: *p* < 0.001)

Since A_1_AR agonists induce pharmacologic effects on various bodily physiological functions (see review by Borea et al. 2018; Effendi et al. 2020), the following are a collection of the actions of ENBA we have observed in association with our main objective of investigating ENBA’s neuroprotective effects following GD exposure.

Beyond suppression of neuronal activity in the CNS, A_1_AR agonists had significant peripheral effects, such as hypothermia (Carlin et al. 2017). We defined the hypothermic body temperature range at 32.0 ± 2.0 °C in our studies. All ENBA-included treatments with and without GD exposure induced some degree of hypothermia, bradycardia (i.e., reduction of heart rate), and sedation in rats (Thomas et al. 2019; Loughery et al. 2021). Similar effects are observed in the KIKO mice following ENBA treatment. In saline exposure (Fig. 5A), mean baseline body temperatures ranged between 36.9 ± 0.7 °C to 37.4 ± 0.4 °C across all treatment groups. After AP (control) treatment, there was no appreciable change in body temperature from baseline throughout the study (mean range was 36.8 ± 0.7 °C to 36.0 ± 0.6 °C). The MDZ group’s mean body temperatures ranged from 35.0 ± 1.5 °C to 36.3 ± 0.6 °C after treatment. Within the MDZ group, treatment induced a significant reduced body temperature from baseline beginning at 1 h (35.1 ± 1.2 °C; DF = 7, *P* = 0.034), through 4 h after treatment (35.8 ± 0.6 °C; DF = 7, *P* = 0.004). When compared directly against the control (AP group), MDZ had significantly lower body temperatures intermittently after treatment (Saline/AP vs. MDZ: 15 min (DF = 11.1, *P* = 0.031); at 30 min (DF = 10.9, *P* = 0.035); and at 3 h (DF = 13.5, *P* = 0.027). Mean body temperatures for MDZ corrected by 4 h after treatment (35.8 ± 0.6 °C), and were comparable to AP. On the other hand, the reduction of temperature by ENBA (10 mg/kg, i.p.) was large and prevalent for 24 h. The rate of change for body temperature within the first h after ENBA was -0.13 °C per min and for MDZ + ENBA was -0.15 °C per min. After only 4 min, ENBA administration reduced body temperature significantly compared to baseline (ENBA: 35.7 ± 0.7 °C (DF = 7, *P* = 0.018); MDZ + ENBA: 35.5 ± 0.7 °C (DF = 7, *P* = 0.042)). ENBA and MDZ + ENBA groups sustained the hypothermic effect throughout the 5 h after treatment. They presented with a baseline of 37.4 ± 0.4 °C (8) for ENBA and 36.9 ± 0.7 °C (8) for MDZ + ENBA then reducing to 21.5 ± 0.6 °C and 21.5 ± 0.9 °C, respectively, at 5 h (300 min) after treatment, or a reduction of 42% from baseline. These groups continued to experience a decrease in body temperature overnight; at 24 h after treatment, temperatures remained severely hypothermic compared to baseline (ENBA: 20.5 ± 0.7 °C (DF = 7, *P* =  < 0.001); MDZ + ENBA: 21.1 ± 1.0 °C (DF = 7, *P* =  < 0.001). However, by 48 h, the temperatures all returned to a normal range and maintained as such to the end of 14 days. There was no apparent difference between the rate of change in these two ENBA-treated groups overall, showing that MDZ, when part of the treatment regimen, had no added effect. Thus, the body temperature records showed that ENBA (10 mg/kg) induced significant hypothermia that lasted for about a day under saline control exposure condition.

For mice exposed to GD (Fig. 5B), mean baseline body temperatures were similar for all groups. Specifically, 37.3 ± 0.6 °C (10) for AP, 37.4 ± 0.4 °C (10) for MDZ, 37.7 ± 0.4 °C (10) for ENBA, and 37.6 ± 0.4 °C (10) for MDZ + ENBA. After exposure, temperatures ranged between 34.7 ± 0.5 °C to 35.2 ± 0.5 °C at 15 min after SSE onset. All groups were significantly below baseline before treatment (vs. 15 min PE: AP (DF = 9, *P* =  < 0.001); MDZ (DF = 10, *P* =  < 0.001); ENBA (DF = 9, *P* =  < 0.001); and MDZ + ENBA (DF = 9, *P* =  < 0.001)). In addition, all groups were not significantly different before treatment administration, when compared to the control AP designated mice.

Within 4 min of treatment, an equivalent and significant reduction on body temperature was observed within the AP (control) and MDZ treatment groups (AP: 33.0 ± 1.6 °C (DF = 9, *P* =  < 0.001); MDZ: 32.8 ± 2.0 °C (DF = 9, *P* =  < 0.001)). They were not significantly different from each other throughout the 5 h while presenting with a reduction of ~ 12% from baseline body temperature, which then could be a factor attributed to the GD exposure. A Mann–Whitney U test was performed to compare average body temperature between the saline exposed AP-treated group to the GD exposed AP-treated group. The temperature was significantly different between the two after exposure, (8 min PE: Mann–Whitney U = 6.5, q-value = 0.0015). The same test was performed between the saline exposed MDZ-treated group to the GD exposed MDZ-treated group and had similar results after exposure, (8 min PE: Mann–Whitney U = 2, q-value = 0.00016). Across exposures for both treatment groups, similar trends were observed regardless of AP or MDZ treatment (Supplemental Table 11), showing that GD has a physiologically relevant impact on body temperature overall. By the 24 h timepoint, AP had mean body temperatures comparable to baseline (35.9 ± 2.3 °C), but the MDZ group, compared to baseline, was hypothermic (32.1 ± 3.9 °C; DF = 8, *P* =  < 0.001). After 24 h observations, animals (regardless of treatment) who were at or below 35 °C were placed on the heat pad in the animal holding room and monitored daily. Animals were taken off heat support when temperatures were above 35.1 °C and had regained mobility. The body temperature of the AP group returned to 37.0 ± 1.3 °C on the second day on average. However, the AP + MDZ group remained between the range of 32.1 ± 3.9 °C to 32.3 ± 0.0 °C for the next 3 days before returning to 36.8 ± 0.5 °C on the 4th day after GD exposure. Thus, GD exposure might have extended the duration of mild hypothermia by MDZ when compared to the saline exposure groups.

On the other hand, the administration of ENBA (15 mg/kg, i.p.) and MDZ + ENBA treatment caused body temperature to reduce by over 10 °C from normal temperature ranges over 2 h. The rate of change for body temperature within the first h after ENBA was -0.13 °C per min and for MDZ + ENBA was -0.13 °C per min. At 15 min after GD and before treatment, body temperature was already reduced significantly to 34.7** ± **0.5 °C for ENBA designated mice (vs. baseline: DF = 9, *P* =  < 0.001) and 35.2 ± 0.5 °C for MDZ + ENBA designated mice (vs. baseline: DF = 9, *P* =  < 0.001). After only 4 min, body temperature dropped further compared to group baseline (ENBA: 33.5 ± 0.6 °C (DF = 9, *P* =  < 0.001); MDZ + ENBA: 34.0 ± 0.7 °C (DF = 9, *P* =  < 0.001)).

When compared changes in body temperature against the control AP group, the ENBA group presented with a significant difference at 4 min after treatment (ENBA: 33.5 ± 0.6 °C (DF = 16.1, *P* = 0.029)). After 15 min from treatment, both ENBA-treated groups were significantly lower compared to AP (ENBA: 31.0 ± 0.7 °C (DF = 13.3, *P* =  < 0.001); MDZ + ENBA: 31.8 ± 0.5 °C (DF = 11.2, *P* =  < 0.001).

At 5 h after treatment, both ENBA-treated groups had a severe reduction of ~ 42% from baseline in body temperatures with ENBA at 22.4 ± 3.7 °C (DF = 8, *P* =  < 0.001) and MDZ + ENBA at 21.2 ± 0.9 °C (DF = 9, *P* =  < 0.001). These two groups sustained the hypothermic body temperature overnight; the 24-h average temperatures were 23.6 ± 3.6 °C and 22.3 ± 3.4 °C, respectively. By 48 h, temperatures returned to a normal range of 38.4 ± 0.8 °C and 37.7 ± 0.8 °C, respectively, and were maintained as such to the end of 14 days. There was no difference between the rate of change for ENBA and MDZ + ENBA treatments, indicating that ENBA was the main factor in the cause of hypothermia. The data showed that ENBA (15 mg/kg) induced a marked reduction of body temperature that lasted for 24 h following GD exposure and 15 min of ongoing SSE.

When comparing the average body temperature between the saline exposed ENBA-treated group to the GD exposed ENBA-treated group, temperature was significantly different between the two after exposure, (4 min PE: Mann–Whitney U = 0, q-value = 0.00015) until 1 h due to the effect of GD on temperature significantly reducing temperature before treatment administration. However, they were no longer different by 2 h after treatment. This difference in temperature effect from agent was shorter between the saline exposed MDZ + ENBA group to the GD exposed MDZ + ENBA group, where by 8 min after treatment temperature was no longer significantly different between the two.

### Effects on Heart Rates (Fig. 6; Supplemental Fig. 2; Supplemental Table 12–15)

**Fig. 6:**
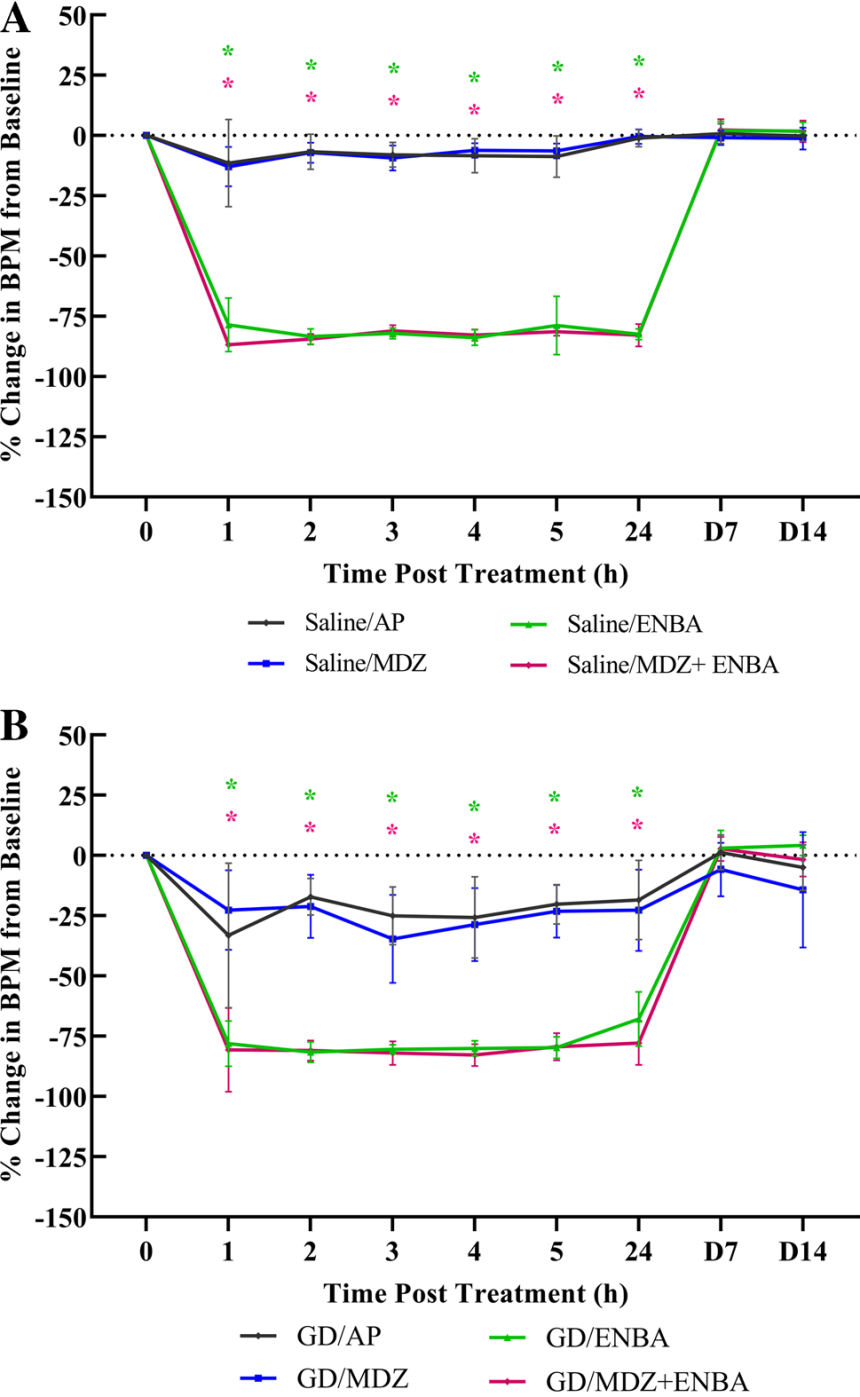
Fourteen-day percent change in heart rate (HR) measurements following saline (sham) or soman (GD) exposure and treatments. KIKO mice were treated with HI-6 (125 mg/kg, i,p,) 30 min prior to challenge with a dose of saline (6A) or GD (33 µg/kg, s.c.; 6B) and treated one min later with atropine methyl nitrate (2 mg/kg for saline-exposed, 4 mg/kg for GD-exposed, i.p.). Animals were randomly assigned to one of the 4 treatment groups: AP (atropine sulfate + 2-PAM), MDZ (AP + midazolam), ENBA (AP + ENBA), or MDZ + ENBA (AP + midazolam + ENBA). Treatments were administrated i.p. at 15 min after GD-induced EEG seizure onset or relevant time after saline exposure groups. Heart rate (HR) was recorded hourly on experimental day for 5 h, at 24 h, and on day 7 and 14 following exposure. The percent change from baseline was calculated as: (100 x (24 h HR – baseline HR))/baseline HR for each animal before averaging. HR was analyzed using REMLS/Mixed effect model and Dunn’s multiple comparisons for within and between group comparisons within each exposure. (*) indicates significantly different (*p* ≤ 0.05) from respective AP treatment group. ENBA and MDZ + ENBA negatively impacted HR within 1 h of treatment compared to AP treatment alone for saline (6A) and GD (6B) exposures (saline exposure: [AP vs. ENBA and MDZ + ENBA: *p* < 0.001]; GD exposure: [AP vs. ENBA: p = 0.003; AP vs. MDZ + ENBA: p = 0.002]). This effect was observed for 24 h after administration (saline exposure: [AP vs. ENBA and MDZ + ENBA: *p* < 0.001]; GD exposure: [AP vs. ENBA and MDZ + ENBA: *p* < 0.001]). The suppression of HR was readily reversible with gentle warming to overcome sedation after ENBA and HR readings were comparable to their respective AP treatment group by day 7

In addition to lowering body temperature, A_1_AR agonists rapidly reduced heart rate (HR). It is well known that adenosine reduces HR and impulse generation in heart tissues (Drury and Szent-Györgyi 1929; Headrick et al. 2011). At the start of the study, all KIKO mice HR averaged from 729 ± 41 to 770 ± 34 beats per min (BPM) at baseline. Within the AP group, there was no significant difference in percent change of HR from baseline 1 h after treatment. Within the MDZ group, treatment caused a significant reduction in HR from baseline from 1 h throughout the 5 h after treatment (vs. baseline at 1 h: DF = 7, *P* = 0.015; at 5 h: DF = 7, *P* = 0.003). Although throughout the saline exposure (Fig. 6A), the MDZ treatment group was not significantly different from the AP (control) group with mean HR change from baseline ranging from -11.4 ± 18.1% and -13.0 ± 8.2% (at h 1), to -8.8 ± 8.5% and -6.4 ± 3.0% (at h 5), for AP and MDZ respectively. Within the ENBA group, treatment induced a -78.5 ± 11.1% change from baseline by h 1 (DF = 7, *P* =  < 0.001), while MDZ + ENBA treatment induced an even lower change of -86.8 ± 0.7% from baseline at h 1 (DF = 6, *P* =  < 0.001). This induction of bradycardia was reliably observed within min of ENBA treatment, quickly accompanied by lethargy and pallor (Loughery et al. 2021). The depression of HR relative to baseline persisted to the 24-h endpoint for ENBA treatment alone or in combination with MDZ, reaching -82.5 ±   2.3% and -82.8 ± 4.6% of baseline HR (i.e., 132 ± 17 and 126 ± 33 BPM), respectively, after saline exposure with significantly lower rates compared to the control treatment (AP vs. ENBA at 24 h: DF = 11.9, *P* =  < 0.001; AP vs. MDZ + ENBA at 24 h: DF = 13.1, *P* =  < 0.001). The bradycardia was no longer detected at the 7th day measurement and beyond, both ENBA-treated groups were at or above average baseline HR. However, in the AP and AP + MDZ groups the HRs were relatively unchanged from baseline values.

For GD-exposed KIKO mice (Fig. 6B), the AP and MDZ treatment groups had HR equally impacted for 24 h after exposure, when comparing with saline-exposed groups. To compare across exposures, a Mann–Whitney U test was performed to compare the percent change in HR from baseline between the saline exposed AP-treated group to the GD exposed AP-treated group. The change was not significantly different between the two exposures after 1 h and remained comparable at 5 h. The same test was performed between the saline exposed MDZ-treated group to the GD exposed MDZ-treated group and there was no significant difference 1 h after treatment. A significant reduction began at 3 h after treatment and did not resolve by 5 h (3 h PT: Mann–Whitney U = 4, q-value = 0.0023; Supplemental Table 15). Within the AP group, a significant reduction in HR from baseline began at 1 h and persisted until 5 h after treatment, (-33.2 ± 30.0% Δ from baseline at 1 h: DF = 9, *P* = 0.036; to -18.5 ± 16.4% Δ from baseline at 5 h: DF = 9, *P* =  < 0.001). Within the MDZ group, a similar reduction in HR from baseline began at 1 h and did not resolve 24 h after treatment, (-22.0 ± 17.3% Δ from baseline at 1 h: DF = 9, *P* = 0.016; -24.3 ± 17.3% Δ from baseline at 24 h: DF = 7, *P* = 0.022). Although, the change in HR of the MDZ treated group was not significantly different from the AP control group throughout the 24 h after exposure.

Similar to the effect observed within the saline exposure, ENBA and MDZ + ENBA treatment caused large reductions in HR from baseline. Presenting at 160 ± 72 BPM for ENBA and 148 ± 134 BPM for MDZ + ENBA at 1 h after treatment. This is a severe reduction from baseline for both groups, (-78.1 ± 9.4% Δ from baseline for ENBA at 1 h: DF = 7, *P* =  < 0.001; − 80.7 ± 17.4% Δ from baseline for MDZ + ENBA at 1 h: DF = 7, *P* =  < 0.001). HR maintained at this level for both groups throughout the 24 h period. When compared between exposures, treatment with ENBA or MDZ + ENBA did not present with a significant difference at any point (Supplemental Table 15). HR was measured again on the 7th day after exposure, where both ENBA-treated groups presented back to baseline range (Supplemental Table 14a).

### Effects on Motor Activity and General State of Movement (Fig. 7, Table 6)

**Fig. 7:**
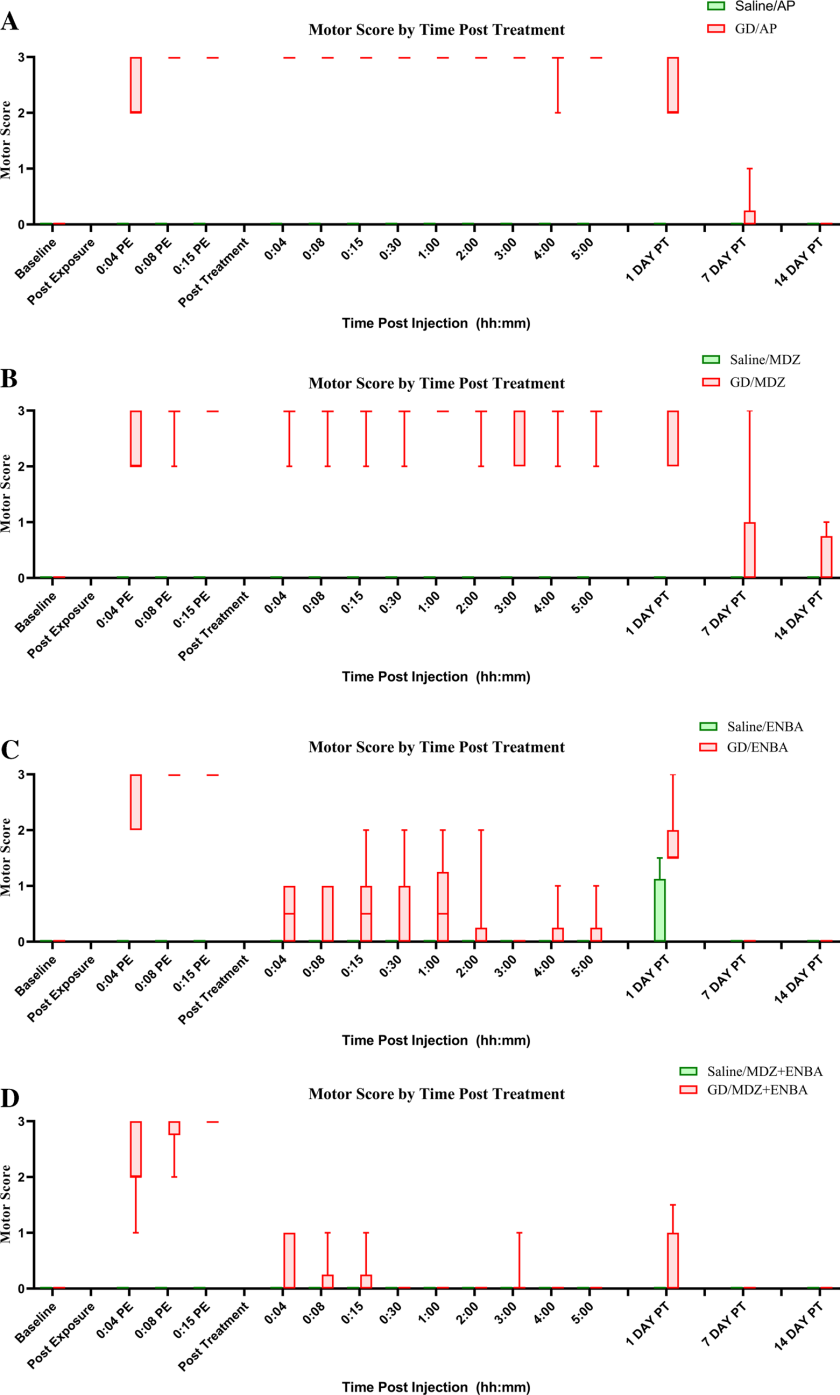
Fourteen-day motor activity (convulsions) recordings following saline (sham) or soman (GD) exposure and treatments. KIKO mice were pretreated with HI-6 (125 mg/kg, i,p,) 30 min prior to challenge with a dose of saline (  ) or GD (33 µg/kg, s.c.;  ) and treated one min later with atropine methyl nitrate (2 mg/kg for saline-exposed, 4 mg/kg for GD-exposed, i.p.). Animals were randomly assigned to one of the 4 treatment groups: AP (atropine sulfate + 2-PAM; 7A), MDZ (AP + midazolam; 7B), ENBA (AP + ENBA; 7C), and MDZ + ENBA (AP + midazolam + ENBA; 7D). Treatments were administrated i.p. at 15 min after GD-induced EEG seizure onset or relevant time after saline exposure groups. Toxic motor activity was recorded and statistically compared for 5 h during experimental day, at 24 h, and on day 7 and 14 following exposure. Toxic motor score assessed physical seizure activity for severity (0 = none, 1 = fasciculations, 2 = localized tremors, 3 = full body convulsions). Treatment with ENBA and MDZ + ENBA equally reduced motor convulsions from a median score of 3 to a median score of 0 within 5 min after administration

Treatments including ENBA produced rapid decrements of motor activity and subsequent sedation regardless of saline or GD exposure (Table 6 and Fig. 7). A functional observational battery (FOB), including assessments for startle reflex, righting reflex, motor activity, gait, and arousal was performed on each animal at regular intervals after saline and GD exposure and treatments (Tables 1 and 6). Of the various functional assessments, ambulation (e.g., general state of movement) was the most representative measure of function or incapacitation for these mice. That is, the ability to ambulate even slowly indicated the return of a righting reflex and at least a moderate degree of arousal or cognitive function. ENBA at 10 mg/kg in saline-exposed and 15 mg/kg in GD-exposed mice experienced severe immobility and depth of sedation; they became unconscious, which corresponded with the suppression of EEG activity. The suppression of EEG activity progressed rapidly within min after ENBA treatment (Table 2, Fig. 3). Subjects became unresponsive, displayed complete loss of muscle tension, and lacked a righting reflex. These effects lasted for more than 24 h and returned to baseline by the 7th day of observation time point. In saline-exposed mice (Fig. 7; green box), treatment with either AP (Fig. 7A) or AP + MDZ (Fig. 7B) showed no deficit in motor functions (score = 0, Table 6). However, in GD-exposed animals (Fig. 7, red box), these two treatment groups induced a higher degree of impaired movement and loss of muscle tension (scores ranges 1.2 – 3.0, Table 6). AP (Fig. 7A) and MDZ (Fig. 7B) treatments were not able to prevent or stop GD induced toxicity on muscular activity in these mice. They were prostrated and their muscular convulsions unabated. The most toxic effect of GD was the muscular convulsive activity, which was attenuated quickly and completely by the two ENBA treatment groups (Fig. 7C and D), clearly displaying the anticonvulsive efficacy of ENBA against GD-induced chronic and tonic muscular toxicity.

**Table 6:** Summary of Functional Observational Battey (FOB) scores for toxic signs after saline and soman (GD) exposures and treatments

Observation	Treatment	Saline exposure				GD exposure			
	Mean range	AP	MDZ	ENBA	MDZ + ENBA	AP	MDZ	ENBA	MDZ + ENBA
Righting Reflex	Before	0.0–0.0	0.0–0.0	0.0–0.0	0.0–0.0	0.0–0.1	0.3–0.5	0.2–0.3	0.0–0.2
	After (Last 24 h)	0.0–0.0	0.0–0.0	1.0–2.0	1.8–2.0	0.0–0.4	0.2–1.8	0.8–2.0	1.7–2.0
General state of movement	Before	0.0–0.0	0.0–0.0	0.0–0.0	0.0–0.0	1.0–1.1	1.1–1.2	1.0–1.3	0.9–1.0
	After (Last 24 h)	0.0–0.0	0.0–1.1	2.1–3.0	2.8–3.0	1.1–2.1	1.6–3.0	2.1–3.0	2.8–3.0
Toxic motor activity (convulsion)	Before	0.0–0.0	0.0–0.0	0.0–0.0	0.0–0.0	2.4–3.0	2.5–3.0	2.7–3.0	2.2–3.0
	After (Last 24 h)	0.0–0.0	0.0–0.0	0.0–0.4	0.0–0.0	2.4–3.0	2.6–3.0	0.0–1.8	0.0–0.5

Of the many FOB variables ambulation was the most representative measure of incapacitation. EMBA treatments caused loss of muscle tension and righting reflex, but quickly attenuated GD-induced convulsive activity, while AP and MDZ treatments were not able to stop GD-induced toxic motor activity

### Effects on Body Weights (Table 7, Supplemental Table 16–20)

Weight loss can indicate when an animal is under stress or suffering from poor health. At the start of the study, all KIKO mice body weights averaged from 26.3 ± 1.2 to 29.7 ± 1.4 g at baseline. Following saline exposure (Table 7) the AP (control) and MDZ treatment groups presented above baseline from day 2 throughout day 14. On day 7, the AP and MDZ groups had recovered weight above their day 1 average (+ 2.1 ± 2.2% Δ from baseline for AP: (DF = 56, *P* =  < 0.001); + 0.2 ± 3.4% Δ from baseline for MDZ: (DF = 56, *P* = 0.001)). However, at day 2, the ENBA and MDZ + ENBA treatment groups presented -7.6 ± 3.0% and -7.7 ± 3.0% below baseline. This timepoint aligns to the recovery period from ENBA-dependent sedation and hypothermia. Therefore, these effects had an intermittent effect on normal behavior and weight. Weight recovered within 5% of baseline by day 4 for the ENBA group and continued to improve on day 7, presenting significantly improved compared to day 1 after exposure (-1.1 ± 2.1% Δ from baseline for ENBA: DF = 56, *P* = 0.009). The MDZ + ENBA group presented with the smallest change in weight after 24 h among the saline exposed groups. Their weight change was -0.975 ± 1.8% Δ from baseline on day 1 and was significantly above the AP group, with a -7.3 ± 5.2% Δ from baseline on day 1 (Sal/AP vs. MDZ + ENBA: DF = 84, *P* = 0.001)).

**Table 7:** Weight percent change from baseline by exposure and treatment

A. Saline-exposed												
	Saline/AP			Saline/MDZ			Saline/ENBA			Saline/MDZ + ENBA		
Day	AVG	SD	*N*	AVG	SD	*N*	AVG	SD	*N*	AVG	SD	*N*
1	− 7.3	5.2	8	− 4.5	3.2	8	− 4.9	1.9	8	− 1.0	1.8	8
2	0.4	1.1	3	− 1.1	2.4	7	− 7.6	3.0	7	− 7.7	3.0	8
3	1.9	0.0	1	− 1.6	2.9	3	− 7.5	3.4	3	− 8.8	4.0	8
4	1.9	3.2	6	− 1.9	0.0	1	− 4.0	0.0	1	---	---	---
5	1.3	3.2	7	0.7	2.5	5	− 2.2	2.5	5	---	---	---
6	0.7	4.5	7	0.3	3.3	8	− 0.7	2.8	8	− 3.5	2.6	8
7	2.1	2.2	8	0.2	3.4	8	− 1.1	2.1	8	− 4.2	2.5	8
8	1.8	2.6	8	2.2	3.2	8	− 0.7	2.1	8	− 4.6	2.8	8
9	2.2	2.2	3	1.7	3.6	7	− 0.7	1.1	7	− 3.3	2.4	8
10	4.8	0.0	1	1.9	7.2	3	− 0.2	1.4	3	− 3.5	3.7	8
11	1.5	6.1	6	− 1.6	0.0	1	− 1.0	0.0	1	---	---	---
12	− 0.2	5.0	7	− 0.1	2.8	5	− 0.5	1.6	5	---	---	---
13	1.1	4.8	7	0.9	3.5	8	− 1.1	2.0	8	− 3.5	3.1	8
14	0.4	4.8	8	0.0	4.4	8	− 1.1	2.2	8	− 4.4	3.1	8
B. Soman-exposed												
	GD/AP			GD/MDZ			GD/ENBA			GD/MDZ + ENBA		
Day	AVG	SD	*N*	AVG	SD	*N*	AVG	SD	*N*	AVG	SD	*N*
1	− 9.4	4.6	5	− 10.3	4.1	7	− 9.0	5.5	8	− 4.9	3.2	9
2	− 16.3	3.8	4	− 14.9	3.7	6	− 22.1	0.0	1	− 11.3	1.4	3
3	− 12.7	5.6	4	− 13.3	5.1	5	− 27.1	0.0	1	− 6.4	0.0	2
4	---	---	---	− 15.6	0.0	1	− 18.3	5.7	6	− 4.7	7.4	5
5	− 7.2	0.0	1	− 9.8	3.6	2	− 13.5	3.4	6	− 1.9	6.0	6
6	− 11.7	4.9	2	− 7.5	8.0	3	− 15.2	4.4	7	− 2.0	4.1	8
7	− 11.4	7.8	5	− 10.9	8.8	7	− 14.5	4.8	7	− 1.9	4.7	8
8	− 7.1	7.4	5	− 7.3	9.6	7	− 10.6	5.6	6	− 2.4	4.0	8
9	− 4.9	9.1	4	− 5.4	9.5	6	---	---	---	− 3.3	4.2	3
10	− 5.6	9.2	4	− 7.1	8.7	5	---	---	---	− 2.2	1.5	2
11	− 4.8	0.0	1	− 9.1	6.0	2	− 22.8	9.9	6	0.6	3.8	5
12	− 7.6	0.0	1	− 2.1	7.7	2	− 18.4	7.1	6	− 0.8	3.5	6
13	− 1.0	5.3	4	− 0.7	4.6	5	− 23.9	11.1	6	− 0.4	3.9	8
14	− 7.0	14.4	5	− 4.9	12.3	7	− 21.7	13.9	5	− 0.3	3.8	8

Fourteen-day percent change in daily body weight recordings following saline (sham) or soman (GD) exposure and treatments. KIKO mice were pretreated with HI-6 (125 mg/kg, i,p,) 30 min prior to challenge with a dose of saline (6A) or GD (33 µg/kg, s.c.; 6B) and treated one min later with atropine methyl nitrate (2 mg/kg, i.p. for saline exposure and 4 mg/kg, i.p. for GD exposure). Animals were randomly assigned to one of the 4 treatment groups: AP (atropine sulfate + 2-PAM), MDZ (AP + midazolam = standard medical countermeasure), ENBA (AP + ENBA), or MDZ + ENBA (AP + midazolam + ENBA). Treatments were administrated i.p. at 15 min after GD-induced EEG seizure onset or relevant time for saline exposure groups. Body weight was recorded daily for up to 14 days following saline and GD exposure. The percent change from baseline was calculated as: (100 x (24 h weight – baseline weight))/baseline weight for each animal before averaging

However, the MDZ + ENBA had a large impact on weight after they recovered from sedation 48 h after treatment. On day 7, although they were not significantly different from the other ENBA group, the MDZ + ENBA group was significantly below AP with a -3.3 ± 2.4% Δ in weight from baseline (Sal/AP vs. MDZ + ENBA: DF = 84, *P* = 0.001) (Supplemental Table 17b). The shift in weight from baseline remained for the MDZ + ENBA group, presenting at -4.4 ± 3.1% Δ from baseline on 14 day. While this weight fluctuation can be due to prolonged recovery period from the effects of ENBA in combination for MDZ, a 5% shift below baseline is within a relatively safe range without indication of further impact on observed animal health.

Of the GD exposure groups (Table 7), the AP and MDZ treatment groups had weight equally and severely impacted at 24 h after exposure. The AP group presented at -10.3 ± 4.1% Δ from baseline while MDZ group presented at -9.0 ± 5.5% Δ from baseline weight. The ENBA-treated group had moderate weight loss of -4.9 ± 3.2% Δ from baseline, while the MDZ + ENBA treatment had a minimum loss (-1.0 ± 2.2% Δ from baseline at 24 h) which significantly prevented a GD-induced weight loss when compared to the -10.3 ± 4.1% Δ from baseline for the AP treatment group, (DF = 81, *P* = 0.019). Although both ENBA and MDZ + ENBA groups showed the same pattern of weight reduction of approximately 10—11% beginning at day 2 until day 3 after treatment, they slowly gained weight thereafter. By day 7, both ENBA and MDZ + ENBA were at -1.9 ± 4.7% and -1.6 ± 3.0% Δ from baseline, respectively, which were significantly above AP (GD/AP vs. ENBA: DF = 81, *P* = 0.028; GD/AP vs. MDZ + ENBA: DF = 81, *P* = 0.02) and the MDZ groups (GD/MDZ vs. ENBA: DF = 81, *P* = 0.001; GD/MDZ vs. MDZ + ENBA: DF = 81, *P* =  < 0.001).

The AP treatment failed to fully recover body weight after the GD exposure and had body weight severely reduced through day 11 (− 9.1 ± 6.0% Δ from baseline), this was not significantly different from day 1 by day 14 (− 4.9 ± 12.3% Δ from baseline). The MDZ treatment did not prevent this deficit and the group saw the most severe weight loss throughout the 14 days. The average weight at day 1 was − 22.1 ± 0.0% Δ from baseline and was significantly lower at day 14 where the average for the 5 surviving animals was − 21.7 ± 13.9% Δ from baseline (DF = 81, *P* =  < 0.001). Weight recovery after NA exposure can indicate improving health. The AP-treated group experienced a profound reduction of weight while the MDZ group, on the other hand, displayed a deeper and longer duration of reduction and displayed a zig-zag variability among survivors at the level of 70% of baseline body weight, and none of the survivors were able to fully recover to their baseline levels by the 14-day endpoint (remained at level of 78.3 ± 13.9% Δ from baseline). ENBA-treated animals showed faster patterns of body weight recovery than that of AP-treatment group throughout the experiment period.

## Discussion

Our team has been investigating A_1_ adenosine receptor (A_1_AR) stimulation alone as a novel mechanism to shutdown excitotoxicity and to prevent brain damage with some success in a rat seizure model following exposure to organophosphorus nerve agents (NAs) (Thomas and Shih 2014; Thomas et al. 2019; Loughery et al. 2021; Meads et al.^2021^). We have shown that treatment with A_1_AR agonists (such as CPA, CCPA, and ENBA) alone enhances survival and effectively prevents seizure and neuropathology induced by the organophosphorus NAs, such as soman (GD) and sarin (GB), when given immediately (Thomas et al. 2019) and even when given 15, 30, or 60 min after seizure onset in the case of GB exposure in rats (Loughery et al. 2021). The goal of this study was to assess the therapeutic efficacy of an A_1_AR specific agonist, ENBA, when administered intraperitoneally (i.p.) in the newly developed genetically modified KIKO mouse strain (Cerasoli et al. 2019) as a test model at a delayed therapy paradigm. The KIKO mouse strain represents a mammalian system that possesses cholinesterase enzymatic profile similar to humans (i.e., knocked-out mouse-specific plasma CES and knocked-in human-specific AChE) and is, thus, expected to more closely mimic human responses to organophosphorus NA and standard medical countermeasures (MCMs) (Reinhardt 2020).

In this study, we determined the effectiveness of the A_1_AR agonist ENBA, at the minimum effective dose (MED) of 15 mg/kg to suppress ongoing sustained *status epilepticus* (SSE), as an adjunct to current NA MCMs (i.e., atropine sulfate [A], 2-PAM [P], and midazolam [MDZ]) in male KIKO mouse when administered at 15 min after GD-induced SSE. We also ran a saline exposure study as a control to observe the pharmacological effects of these treatments compared to the GD exposure study. For saline exposures, the MED for ENBA that suppresses normal EEG activity in 100% of KIKO mice is 10 mg/kg in lieu of the 15 mg/kg dose following GD exposure (Shih 2023). It’s reasonable to expect that with GD-induced sustained seizure activity, 15 mg/kg of ENBA is required to counter the SSE after a 15 min delay in treatment to produce same level of isoelectric activity in 100% of animals as 10 mg/kg does to saline-exposed animals. Mice were randomly assigned to a total of 8 treatment groups, which were divided into 4 similar groups each for either GD or saline exposure. The 4 treatment groups were AP (designated for atropine sulfate + 2-PAM), MDZ (AP + MDZ), ENBA (AP + ENBA), and MDZ + ENBA (AP + MDZ + ENBA), as shown in Fig. 2. AP treatment served as a control within GD or saline exposures, and the AP group in the saline exposure served as the overall study control. Our goal was to evaluate if the adenosine signaling pathway, especially A_1_AR stimulation as a main mechanism of action, provides the much-needed anticonvulsant and neuroprotective (A/N) effect of ENBA in case of mass NA intoxication and casualties. Thus, our focus became the pharmacologic actions of ENBA on prevention/termination of seizures, mitigation of brain pathology, and enhancement of survival following NA intoxication.

Minutes after ENBA (10 mg/kg) administration, the brain EEG activity of saline-exposed KIKO mice went into an isoelectric state (Table 2). The time to an isoelectric state of deep sedation was rapid and occurred within min (~ 2 min) and the isoelectric EEG activity didn’t recover to baseline activity by 24-h. The duration of action for ENBA lasted for the entire 24-h. No neuropathology was observed at 14 days. Even up to an ENBA dose of 45 mg/kg in saline-exposed mice, no lethality was observed, indicating a relative safety of this chemical (Shih 2023). In the case of GD exposure ENBA was capable of terminating EEG seizures induced by GD quickly (~ 2–3 min) into an isoelectric state (Fig. 3) even when administered 15 min after onset of SSE (Table 2) and significantly reducing both mean change in EEG gamma power (Fig. 4A) and spiking frequency (Fig. 4B), demonstrating that ENBA can provide a therapeutic window of at least 15 min following seizure onset. Similar to saline-exposed mice, the isoelectric EEG activity didn’t recover to baseline activity by the 24-h, and the duration of action for ENBA lasted for over 24-h recording period. These animals displayed no evidence of brain pathology (Table 2). However, mice treated with AP and AP + MDZ suffered from continued SSE, and the pathology scores were 13.7 ± 5.7 and 14.2 ± 2.7, respectively, out of the maximum scores of 24. The anti-seizure and neuroprotective capacity of ENBA treatment clearly demonstrated that even after 15 min of severe EEG seizure activity, the CNS pathology can be protected.

In terms of the A/N effect on A1AR agonists, the results imply that ENBA without or with standard medical countermeasures, in particular the benzodiazepine anticonvulsant MDZ, produces similar pharmacologic actions against GD toxicity. Thus, in circumstances where a delayed anti-seizure remedy is necessary, ENBA can be substituted for MDZ and can carry out the A/N functions for MDZ. Due to their time-sensitive receptor internalization nature (McDonough et al. 2010; Niquet et al. 2016), the benzodiazepine class of anticonvulsants (e.g., diazepam, MDZ) may not be suitable for the sole therapeutic treatment of NA exposures, especially when treatment delays are expected. This fact, coupled with its lower MED requirement (Shih 2023) and quick antiseizure actions and beneficial application up to 60 min after seizure onset (Loughery et al. 2021), makes the A_1_AR agonists an extremely attractive choice as a more versatile A/N antidote for seizure suppression following NA exposure, particularly in case of mass civilian casualties’ scenario. With this lower dose of ENBA, the inherent side effects can be expected to be milder and have a relatively rapid recovery time. The effectiveness of ENBA may be attributable to its highly specific A_1_AR actions.

The available literature of this class of compounds implies that ENBA might have actions other than direct agonism at adenosine receptors, which might be responsible for its A/N effects noted within this report, such as reduction on glutamatergic and cholinergic neurotransmitter release via the A_1_AR (St Hilaire et al. 2009; Borea et al. 2018; Thomas and Shih 2019), apoptosis and inflammatory action (Effendi et al. 2020), therapeutic hypothermia (Thomas and Shih 2019; Munoz et al. 2021, 2024), competition against the NAs (such as sarin and GD) for reversible binding of the active site of AChE (Beste et al. 2018), effect on adenosine homeostasis (Purnell et al. 2021; Liu et al. 2019; Németh et al. 2003, Pastor-Anglada and Pérez-Torras 2018), and other adenosine receptor independent effects (Purnell et al. 2021; Boison 2016; Masino et al. 2011; Liu et al. 2019; Németh et al. 2003; Chen et al. 2019).

Stimulation of peripheral A_1_AR receptors can cause bradycardia, hypothermia, and sedation. This deterred previous researchers (van Helden et al. 1998; Bueters et al. 2002) from further exploring the therapeutic potential of A_1_AR agonist CPA in terminating NA-induced toxicity. However, in this current study, though animals experienced profound hypothermia, bradycardia, and sedation, KIKO mice treated with ENBA recovered well within 48 h following GD exposure. Therapeutic hypothermia has been found to offer neuroprotection against epileptic or ischemic events, so it is hypothesized that it could be one of the mechanisms that afford A_1_AR agonists their neuroprotective efficacy (Thomas and Shih 2019; Niquet et al. 2015; Cilio and Ferriero 2010). A separate study aimed to better understand the role of therapeutic hypothermia in A_1_AR agonist treatment following NA exposure, comparing ENBA treatment with surface cooling, has been conducted and our data showed that following GD intoxication, hypothermia actually promoted survival (Munoz et al. 2021, 2024). While others have reported that CPA’s cardiac suppression limited clinical utility (Joosen et al. 2004), our data clearly demonstrated that acute ENBA-induced bradycardia is not lethal regardless of whether the animal is exposed to GD or not. There was zero lethality for all saline-exposed and ENBA-treated mice up to 45 mg/kg (Shih 2023). Moreover, all ENBA treatments promoted survival after GD exposure when compared with AP and AP + MDZ treatments. The prolonged sedation likely prevented body weight recovery to baseline levels initially for animals treated with ENBA; however, recovery was observed by the third day after treatment, and animals ultimately gained weight faster than AP-treated controls (Table 7). Weight recovery after a stressful event like NA exposure can be an important indicator of an animal’s overall health and wellbeing and likely plays a role in survival. Animals were offered supplemental nutrition to encourage weight recovery over the span of the 14-day study, still those animals treated with AP and especially with AP + MDZ showed mild or no improvement in weight gains and subsequently poor QoL in survivors at 14 days (Table 3).

While ENBA demonstrated the potential to prevent or reduce neuropathology after GD exposure, comprehensive evaluation of neuroprotection requires functional behavioral assessments. Therefore, during this experiment, we assessed ENBA’s neuroprotective efficacy by measuring changes in motor and memory function via a two-way active avoidance shuttle box test with these treatment groups in the KIKO mouse and related those outcomes to neuropathological analysis. Each mouse underwent shuttle box behavioral testing at 7 and 14 days after GD or saline exposure (Harkins et al. 2024). In comparison with standard MCMs (AP + MDZ) group, the addition of ENBA showed decreases in escape latency, response latency, and pre-session crossings, as well as increases in avoidances. Only ENBA-treated groups showed control level inter-trial interval crossings by day 14. These active avoidance shuttle box data suggest that ENBA, alone and as an adjunct to medical treatments, can improve behavioral and cognitive outcomes when given at delayed time points after GD intoxication (for details, see Harkins et al. 2024). Thus, we utilized a genetically modified mouse strain that lacks the mouse-exclusive serum CES gene and knocks-in the human AChE gene as a KIKO seizure model (Shih 2023) for the evaluation of the A/N efficacy of an A_1_AR agonist, ENBA, when administered at 15 min after GD-induced SSE, in the presence or absence of standard MCMs. Following ENBA treatment, SSE was suppressed rapidly and permanently, toxic signs, hypothermia, and bradycardia were recovered by 48 h, neuropathology remained absent, and QoL was markedly enhanced. Treatment with AP + MDZ (= current standard medical countermeasures), death from GD exposure was 50%, while with AP + ENBA treatment, with or without MDZ, survival improved to 80% and 90%, respectively. ENBA is efficacious when brain seizure activity is just getting started or well-established by toxic NA exposure (Thomas et al. 2019; Loughery et al 2021). In the case of delayed treatment, ENBA can be substituted for MDZ. ENBA alone or as an adjunct to standard medical treatments for NAs can improve behavioral and cognitive outcomes when given at delayed time points after GD exposure. Animals with sustained seizure activity who are treated with ENBA mimicked those animals with no seizures and are able to learn faster and retain memories better. Our findings confirm ENBA is a potent anticonvulsant for therapy following GD intoxication and is a potent A/N for both immediate and delayed therapy.

In conclusion, the currently available data from our investigations seem to indicate that A_1_AR agonists, such as ENBA, are potent anticonvulsants and neuroprotectants for organophosphorus NA intoxication and that they can be served as either an anticonvulsant or a neuroprotectant, or both, depending on when they are administered. Based on the side effects of A_1_AR agonists (bradycardia, hypothermia, and sedation), however, it would be undesirable to include them in a pre-treatment administration paradigm like pyridostigmine bromide (Keeler et al. 1991; Charatan 1999) following warnings of possible NA attack. Our data show that this treatment should likely be administered shortly after exposure, since it is efficacious when administered either immediately following NA exposure (e.g., during military operations in the chemical battlefield or a laboratory accident event) or when seizure activity is well-established (e.g., in a civilian mass casualty event) by toxic NA exposure. They can be supplemented as an adjunct to standard MCMs (i.e., atropine sulfate, 2-PAM, and MDZ) for ultimate NA therapy. However, when combined with MCMs, the dose of either the A_1_AR agonist or MDZ (a component of MCM) may need to be reduced and adjusted, due to the potential synergistic inhibitory effects between MDZ and the A_1_AR agonist (Keith et al. 2024). Furthermore, in the case of delayed treatment, an A_1_AR agonist may be substituted for MDZ since MDZ loses its anticonvulsant potency at that time due to internalization of inhibitory GABA receptors (McDonough et al. 2010; Niquet et al. 2016) and an A_1_AR agonist can exert immediate anticonvulsant effects upon administration. Most critically, inclusion of A_1_AR agonists, such as ENBA, in the therapeutic regimen can improve behavioral and cognitive outcomes (Meads et al 2021; Harkins et al. 2024) when given following NA exposure due to its strong anticonvulsant and neuroprotective potency.

## Supplementary Information

Below is the link to the electronic supplementary material.

Supplementary file1 Supplemental Figure 1. Fourteen-day body temperature recordings following saline (sham) exposure or soman (GD) and treatments. KIKO mice were pretreated with HI-6 (125 mg/kg, i,p,) 30 min prior to challenge with a dose of saline (green line) or GD (33 µg/kg, s.c.; red line) and treated one min later with atropine methyl nitrate (2 mg/kg for saline-exposed, 4 mg/kg for GD-exposed, i.p.). Animals were randomly assigned to one of the 4 treatment groups: AP (atropine sulfate+2-PAM, 1A), MDZ (AP+midazolam, 1B), ENBA (AP+ENBA, 1C), or MDZ+ENBA (AP+midazolam+ENBA, 1D). Treatments were administrated i.p. at 15 min after GD-induced EEG seizure onset or relevant time after saline exposure groups. Body temperature was recorded on experimental day for 5 h, at 24 h, and on day 7 and 14 following exposure. Across exposure analysis performed using Mann-Whitney test. (*) indicates datapoints where groups are significantly different (p≤0.05) between exposure groups. GD negatively impacts temperature shortly after exposure, as seen between all treatment groups by 8 min post-exposure. AP and MDZ treatments did not conceal this effect within the experiment day. ENBA treatment (alone and in conjunction with MDZ) made temperatures comparable between exposure groups. (PNG 147 kb)

High resolution image (TIF 1602 kb)

Supplementary file2 (PNG 154 kb)

High resolution image (TIF 1688 kb)

Supplementary file3 (PNG 155 kb)

High resolution image (TIF 1616 kb)

Supplementary file4 (PNG 155 kb)

High resolution image (TIF 1571 kb)

Supplementary file5 Supplemental Figure 2. Fourteen-day percent change in heart rate (HR) measurements following saline (sham) exposure or soman (GD) and treatments. KIKO mice were pretreated with HI-6 (125 mg/kg, i,p,) 30 min prior to challenge with a dose of saline (green line) or GD (33 µg/kg, s.c.; red line) and treated one min later with atropine methyl nitrate (2 mg/kg for saline-exposed, 4 mg.kg for GD-exposed, i.p.). Animals were randomly assigned to one of the 4 treatment groups: AP (atropine sulfate + 2-PAM, 2A), MDZ (AP + midazolam, 2B), ENBA (AP + ENBA, 2C), and MDZ + ENBA (AP + midazolam + ENBA, 2D). Treatments were administrated i.p. at 15 min after GD-induced EEG seizure onset or relevant time after saline exposure groups. Heart rate (HR) was recorded hourly on experimental day for 5 h, at 24 h, and on day 7 and 14 following exposure. The percent change from baseline was calculated as: (100 x (24 h HR – baseline HR))/baseline HR for each animal before averaging. Across exposure analysis performed using Mann-Whitney test. (*) indicates datapoints where groups are significantly different (p≤0.05) between exposure groups. The negative impact of GD on HR was enhanced in the MDZ treatment group (2B). ENBA treatments (alone and in conjunction with MDZ) obscured this effect. (PNG 98 kb)

High resolution image (TIF 1466 kb)

Supplementary file6 (PNG 99 kb)

High resolution image (TIF 1475 kb)

Supplementary file7 (PNG 108 kb)

High resolution image (TIF 1493 kb)

Supplementary file8 (PNG 112 kb)

High resolution image (TIF 1512 kb)

Supplementary file9 (DOCX 73.5 KB)

## Data Availability

Data are provided within the manuscript file.

## Abbreviations

A_1_AR: A_1_ adenosine receptor
AChE: Acetylcholinesterase
A/N: Anticonvulsant and neuroprotectant
AMN: Atropine methyl nitrate
ATSO_4_: Atropine sulfate
CPA: N6-cyclopentaladenosine
CCPA: 2-Chloro-N6-cyclopentyladenosine
CNS: Central nervous system
EEG: Electroencephalogram
ENBA: N-bicyclo-(2.2.1)-hept-2-yl-5'-chloro-5'-deoxyadenosine
FOB: Functional observational battery
GABA: γ-Amino-butyric acid
GB: Sarin
GD: Soman
HI-6: Asoxime chloride
HR: Heart rate
KIKO: Knock in/knock out
MCM: Medical countermeasure
MDZ: Midazolam
NA: Nerve agent
2-PAM: Pralidoxime chloride
PB: Pyridostigmine bromide
QoL: Quality of life
R&D: Research and development
SSE: Sustained *status epilepticus*

## Declarations

### Disclaimers

The views expressed in this report are those of the author(s) and do not reflect the official policy of the Department of Army, Department of Defense, or the U.S. Government. The experimental protocol was approved by the Institutional Animal Care and Use Committee (IACUC) at the U.S. Army Medical Research Institute of Chemical Defense (USAMRICD), and all procedures were conducted in accordance with the principles stated in the Guide for the Care and Use of Laboratory Animals and the Animal Welfare Act of 1966 (P.L. 89–544), as amended. The facility where this research was conducted is fully accredited by the Association for Assessment and Accreditation of Laboratory Animal Care International. The use of trade names does not constitute an official endorsement or approval of the use of such commercial hardware or software. This document may not be cited for purposes of advertisement.
