# Supplementary figures and images for: Pharmacology of Adenosine A1 Receptor Agonist in a Humanized Esterase Mouse Seizure Model Following Soman Intoxication
Source: Neurotox Res. 2024 Sep 4;42(5):41. doi: 10.1007/s12640-024-00717-z (PMC11374867; doi:10.1007/s12640-024-00717-z)

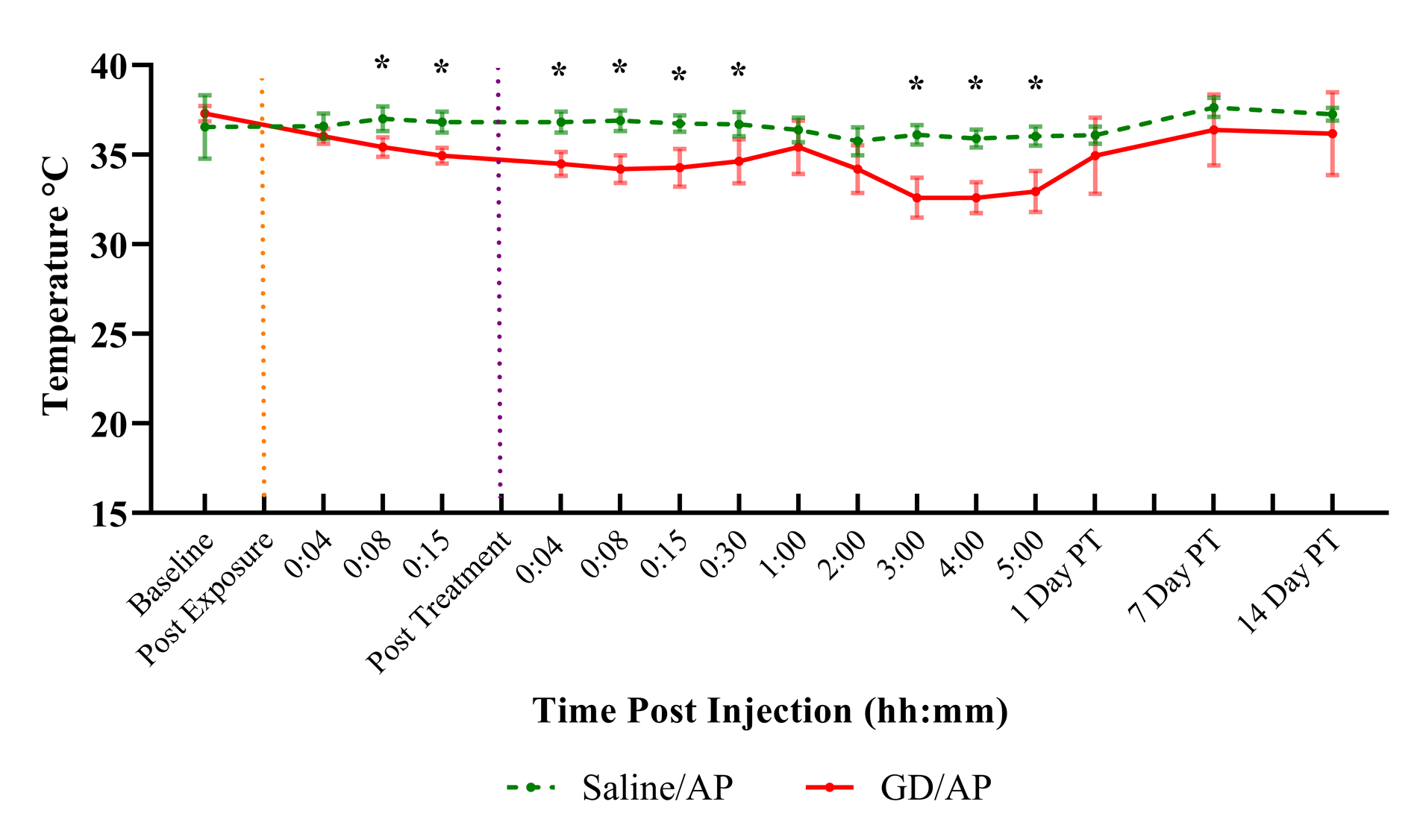

Supplement: Supplementary file 1 — Supplementary file1 Supplemental Figure 1. Fourteen-day body temperature recordings following saline (sham) exposure or soman (GD) and treatments. KIKO mice were pretreated with HI-6 (125 mg/kg, i,p,) 30 min prior to challenge with a dose of saline (green line) or GD (33 µg/kg, s.c.; red line) and treated one min later with atropine methyl nitrate (2 mg/kg for saline-exposed, 4 mg/kg for GD-exposed, i.p.). Animals were randomly assigned to one of the 4 treatment groups: AP (atropine sulfate+2-PAM, 1A), MDZ (AP+midazolam, 1B), ENBA (AP+ENBA, 1C), or MDZ+ENBA (AP+midazolam+ENBA, 1D). Treatments were administrated i.p. at 15 min after GD-induced EEG seizure onset or relevant time after saline exposure groups. Body temperature was recorded on experimental day for 5 h, at 24 h, and on day 7 and 14 following exposure. Across exposure analysis performed using Mann-Whitney test. (*) indicates datapoints where groups are significantly different (p≤0.05) between exposure groups. GD negatively impacts temperature shortly after exposure, as seen between all treatment groups by 8 min post-exposure. AP and MDZ treatments did not conceal this effect within the experiment day. ENBA treatment (alone and in conjunction with MDZ) made temperatures comparable between exposure groups. (PNG 147 kb) [file 12640_2024_717_Fig8_ESM.png]

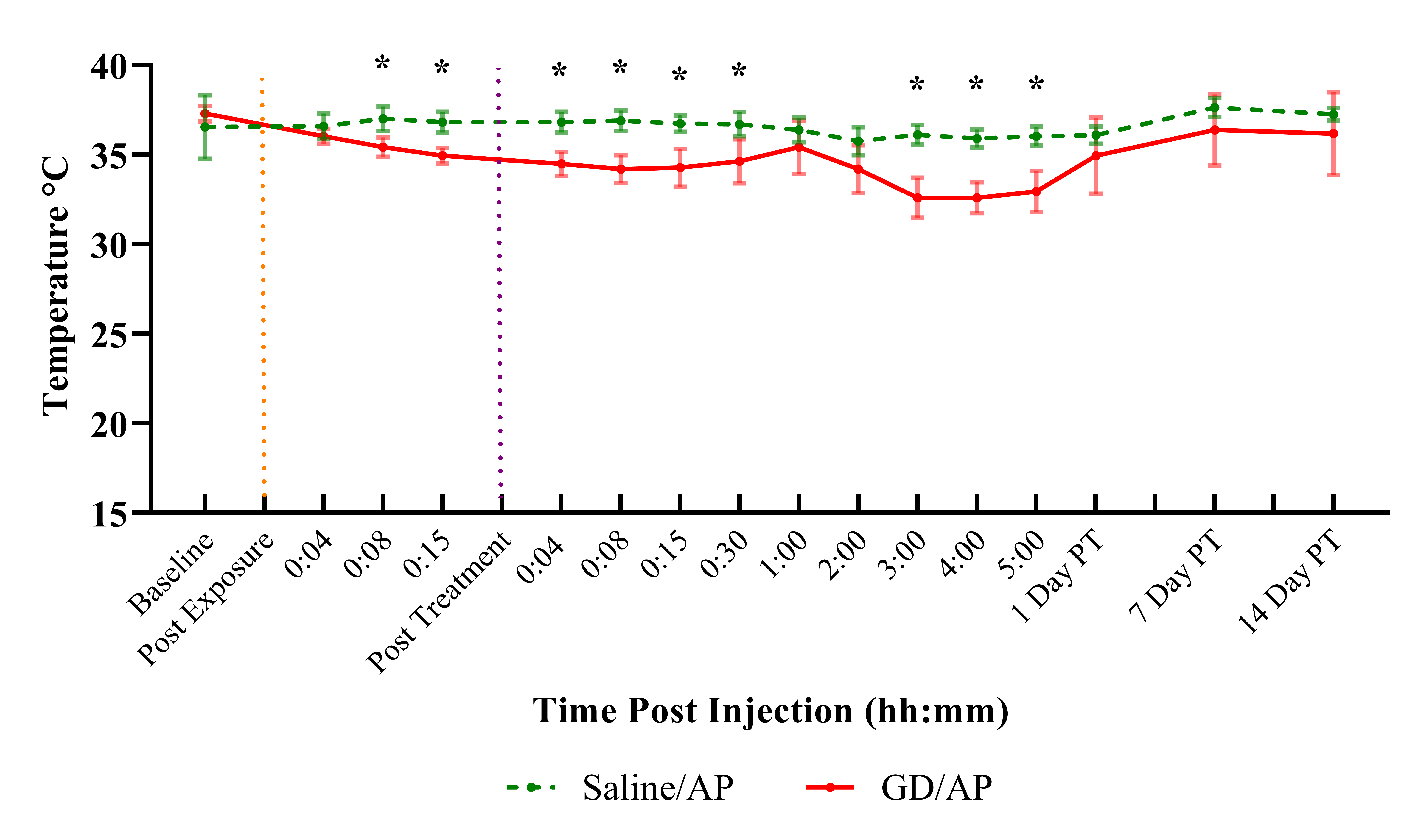

Supplement: Supplementary file 2 — High resolution image (TIF 1602 kb) [file 12640_2024_717_MOESM1_ESM.tif]

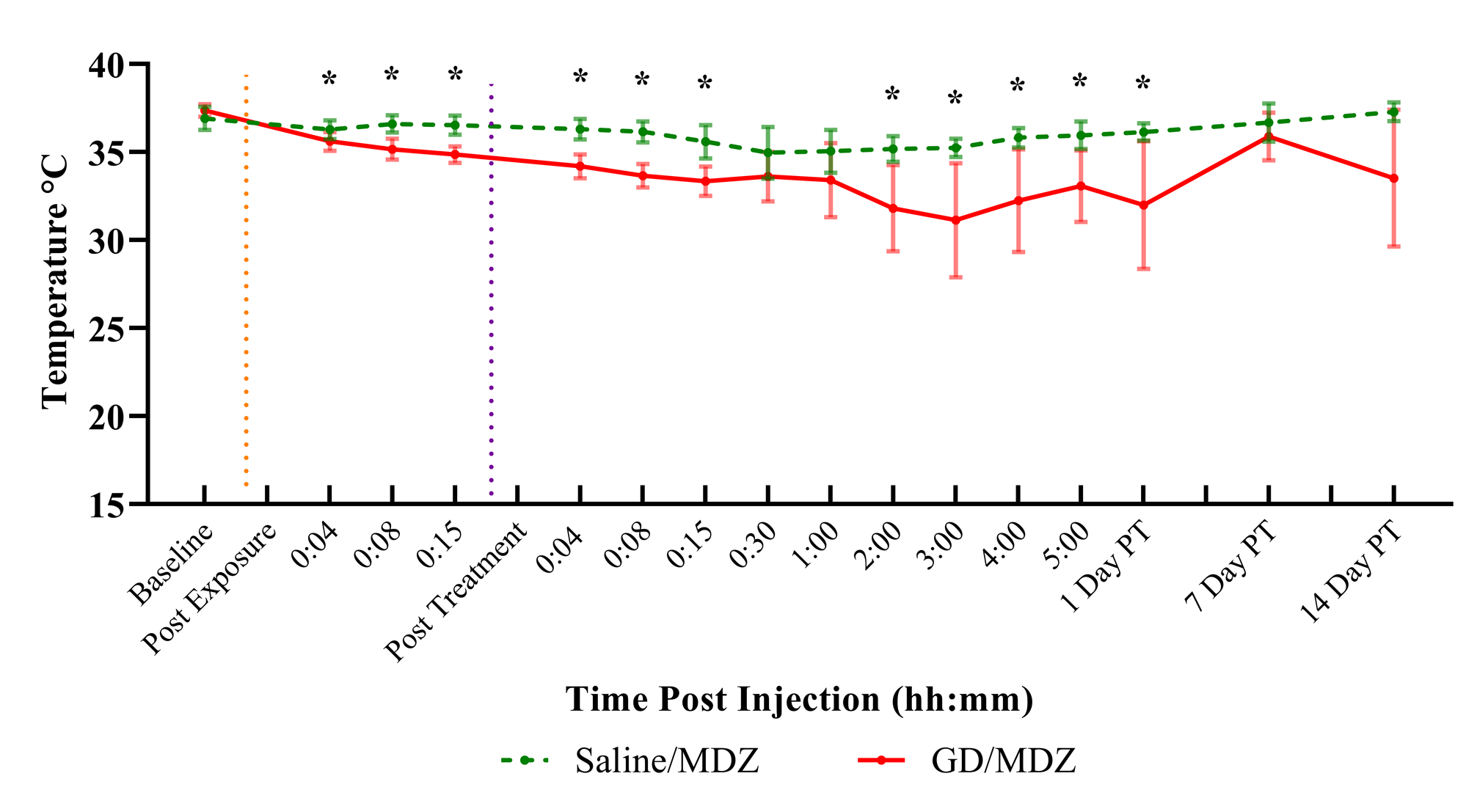

Supplement: Supplementary file 3 — Supplementary file2 (PNG 154 kb) [file 12640_2024_717_Fig9_ESM.png]

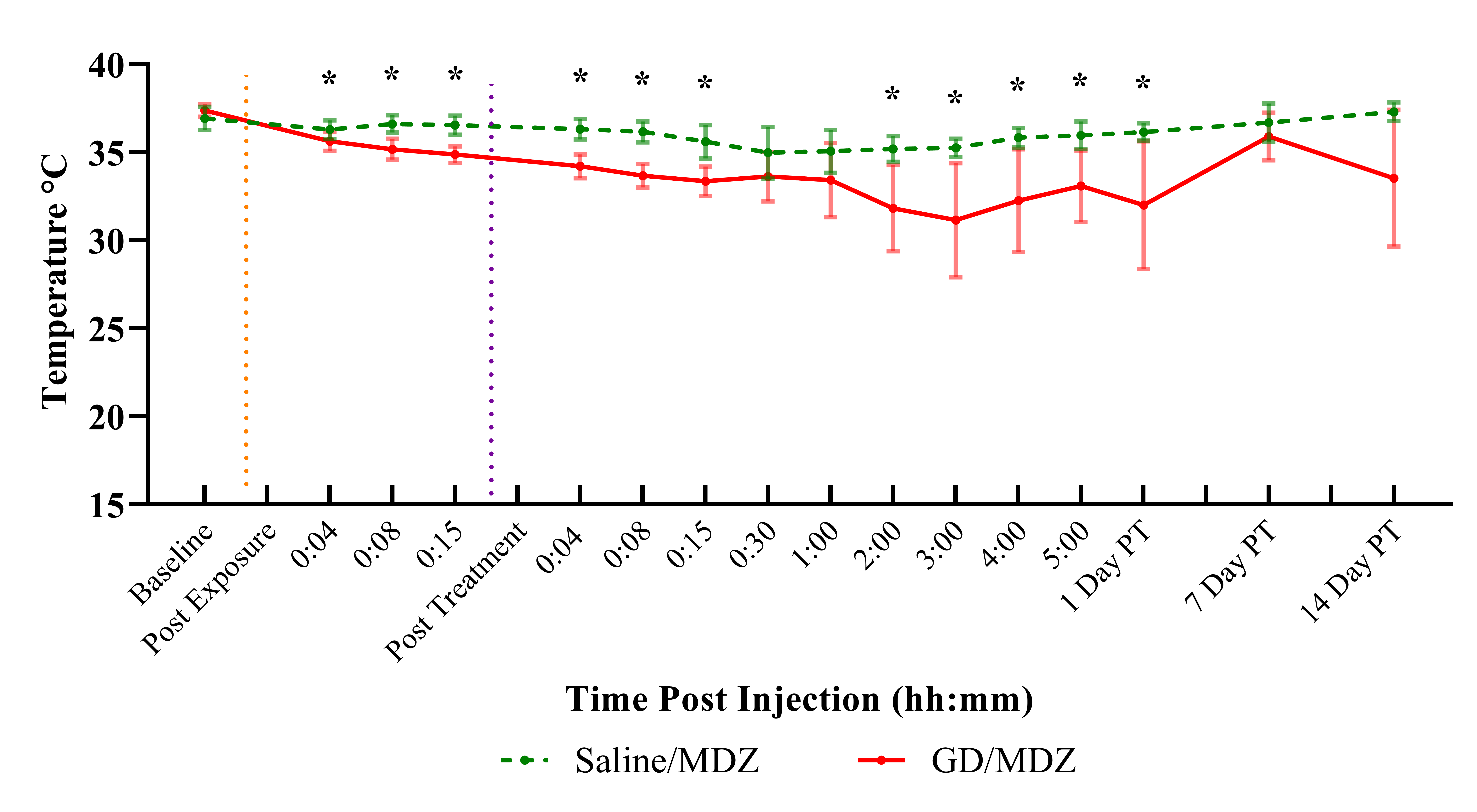

Supplement: Supplementary file 4 — High resolution image (TIF 1688 kb) [file 12640_2024_717_MOESM2_ESM.tif]

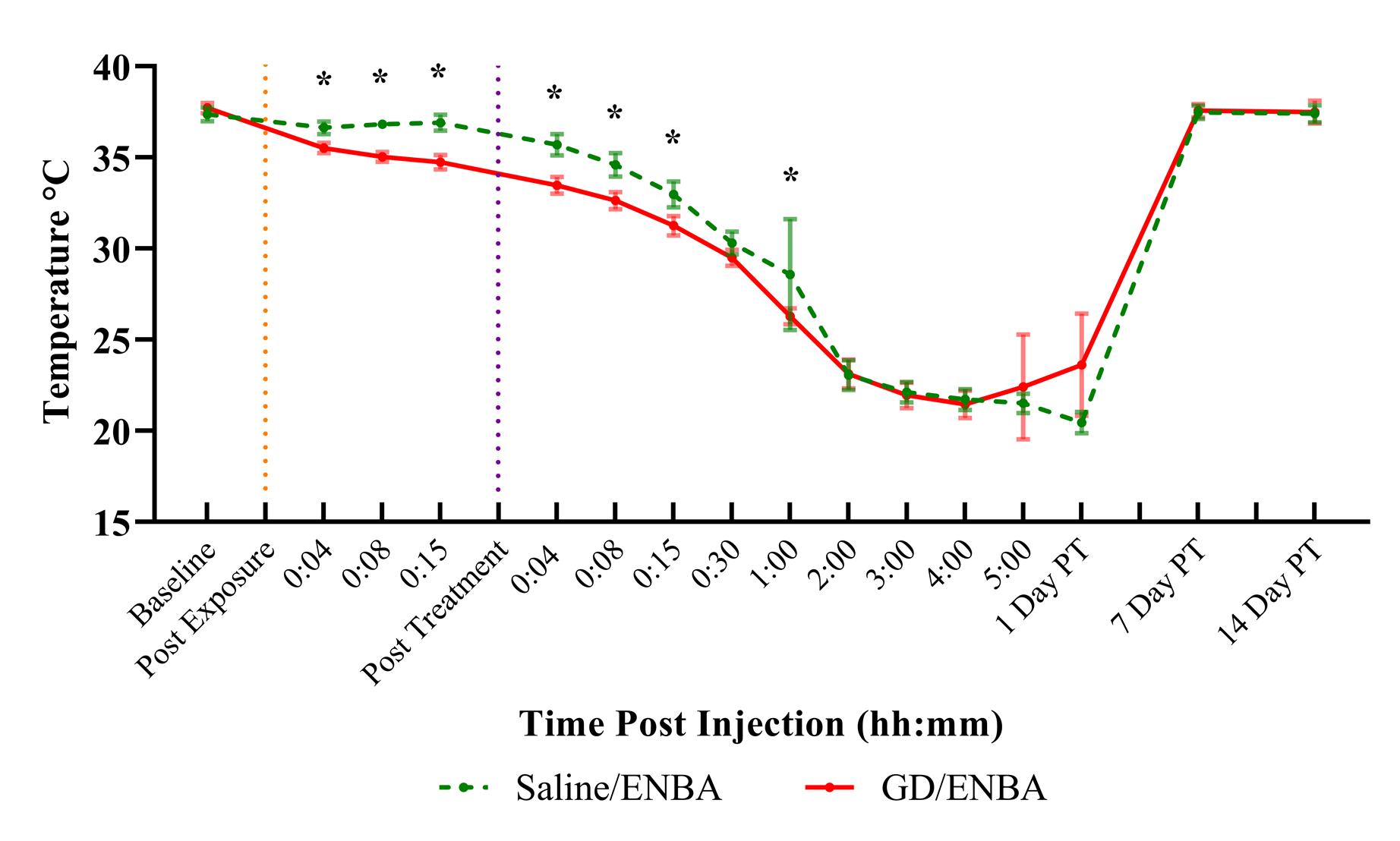

Supplement: Supplementary file 5 — Supplementary file3 (PNG 155 kb) [file 12640_2024_717_Fig10_ESM.png]

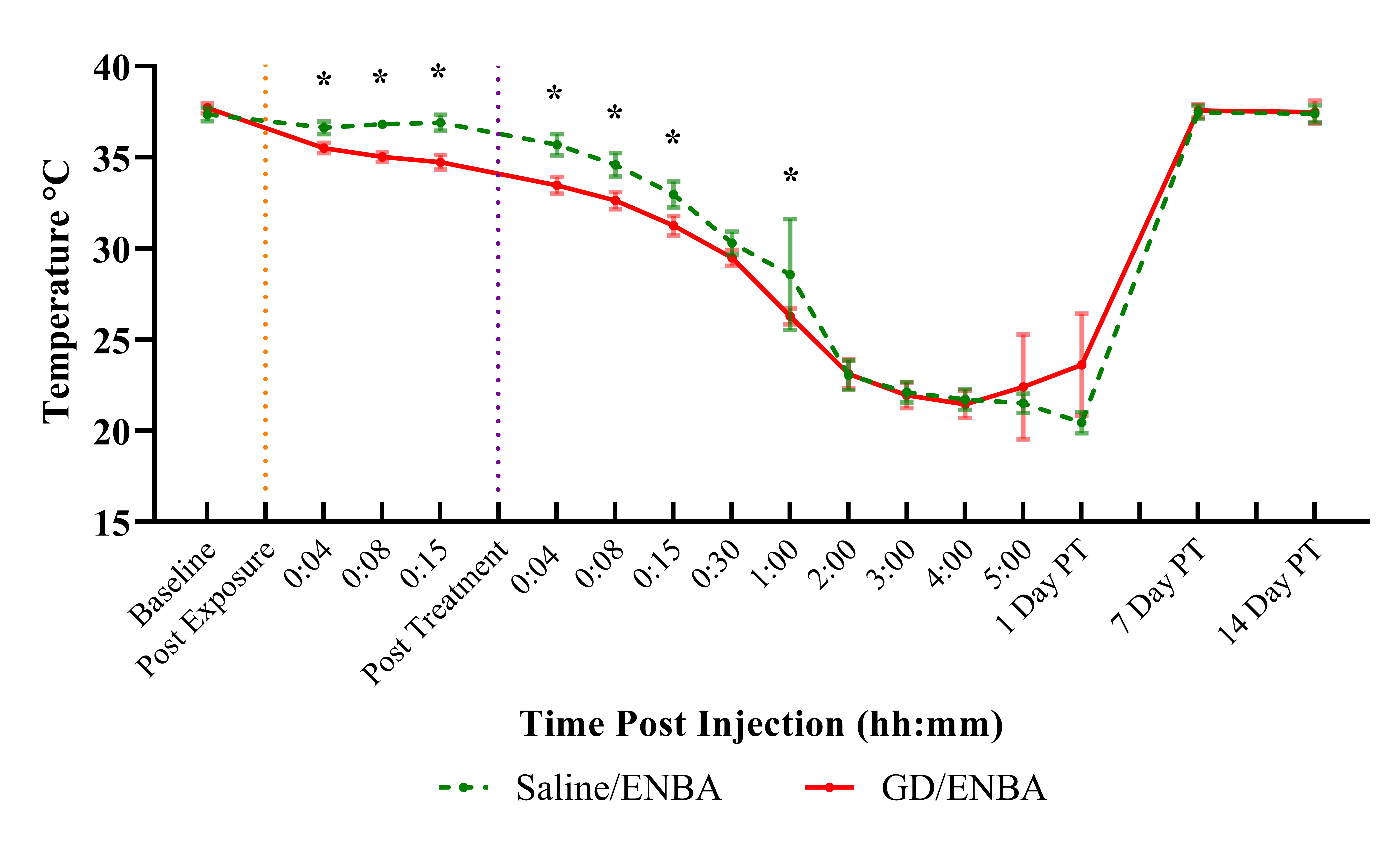

Supplement: Supplementary file 6 — High resolution image (TIF 1616 kb) [file 12640_2024_717_MOESM3_ESM.tif]

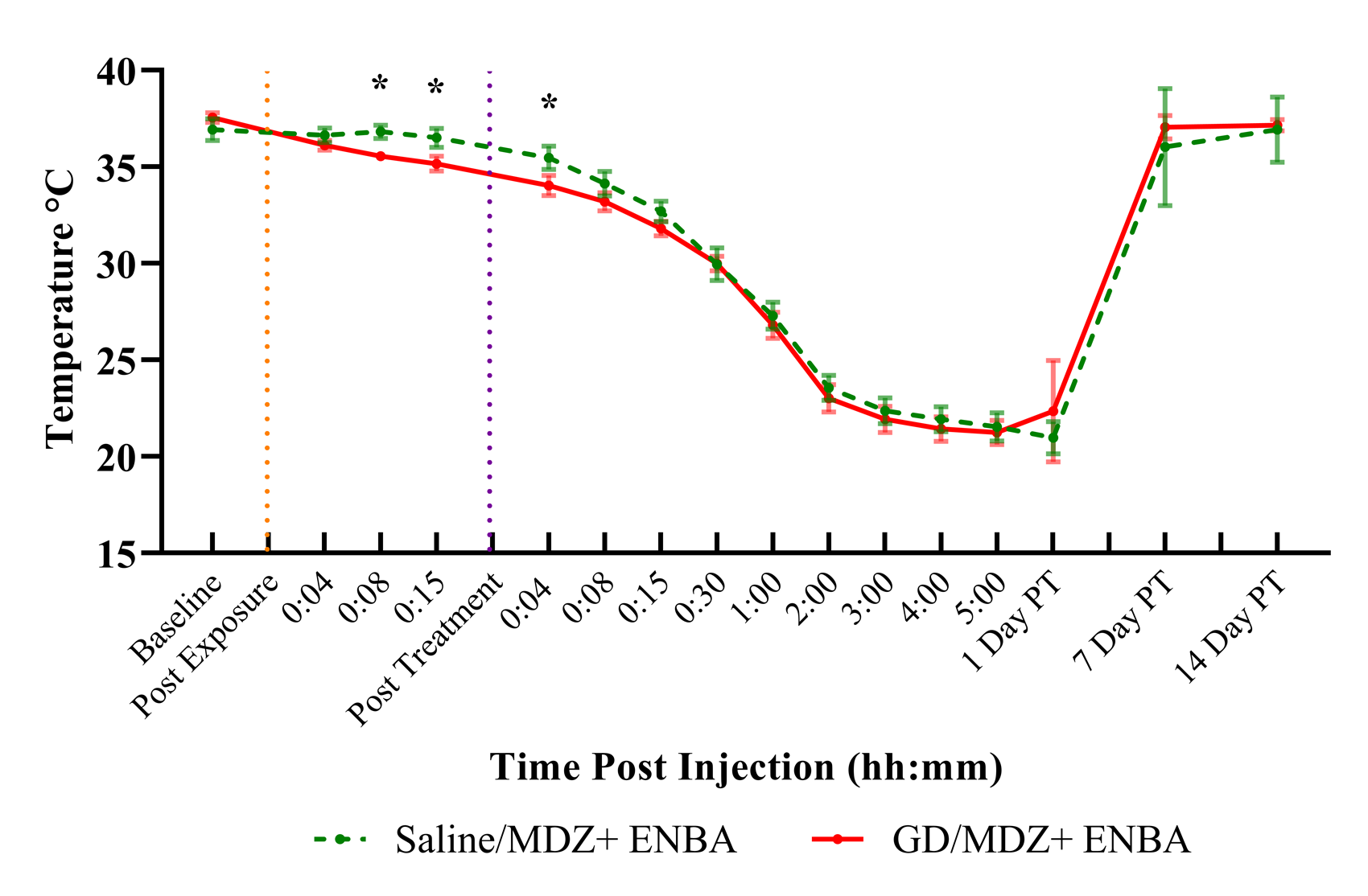

Supplement: Supplementary file 7 — Supplementary file4 (PNG 155 kb) [file 12640_2024_717_Fig11_ESM.png]

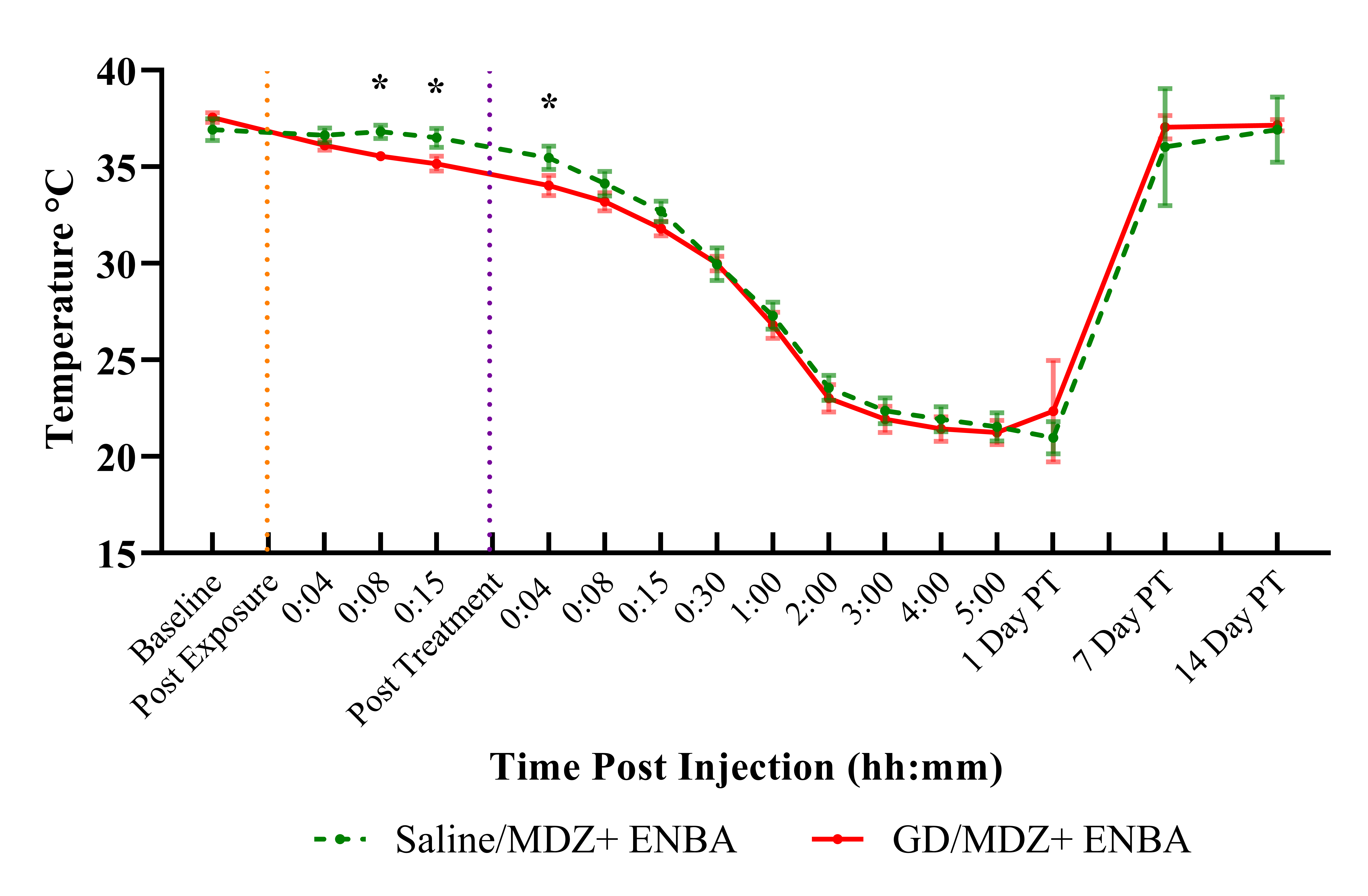

Supplement: Supplementary file 8 — High resolution image (TIF 1571 kb) [file 12640_2024_717_MOESM4_ESM.tif]

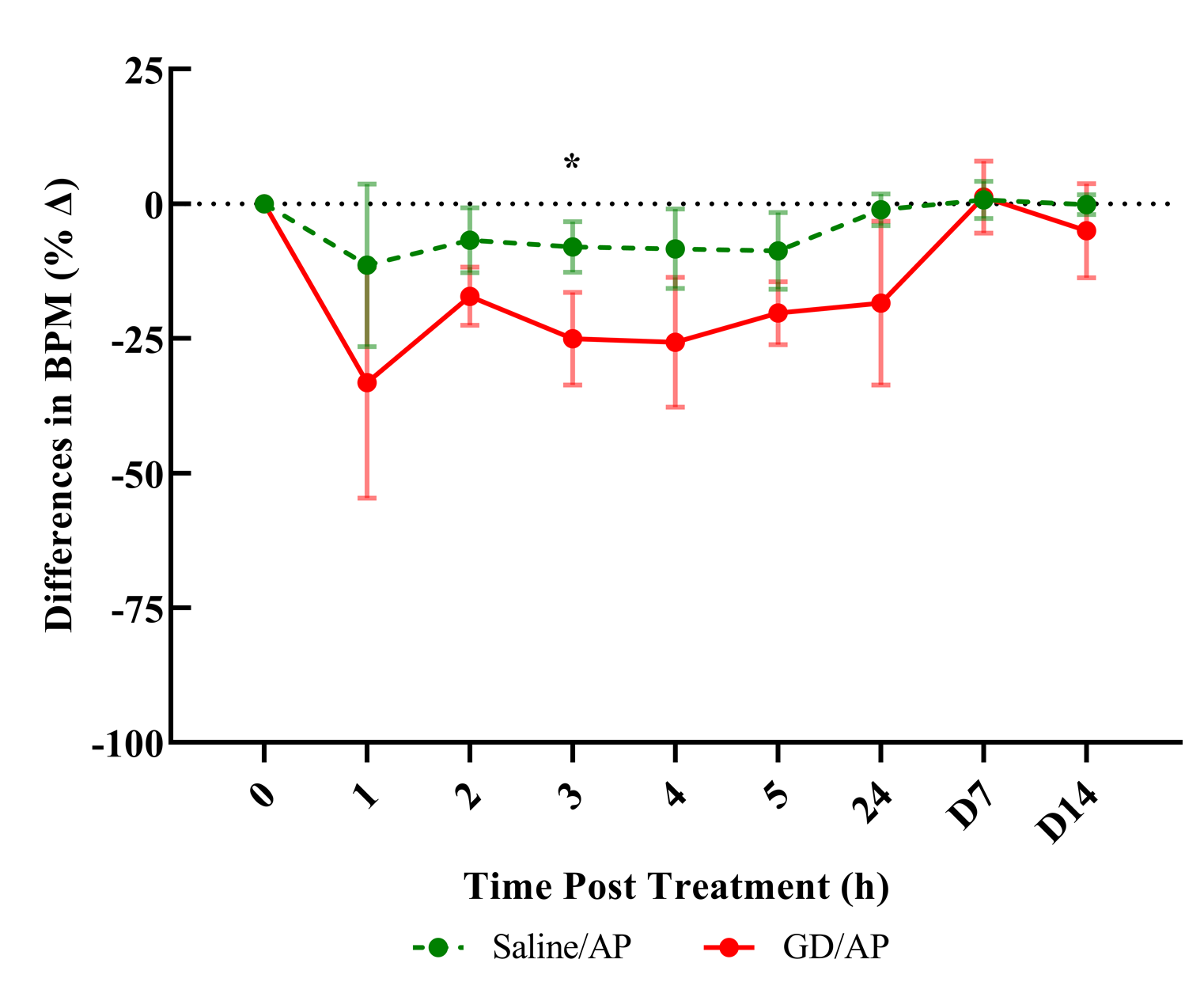

Supplement: Supplementary file 9 — Supplementary file5 Supplemental Figure 2. Fourteen-day percent change in heart rate (HR) measurements following saline (sham) exposure or soman (GD) and treatments. KIKO mice were pretreated with HI-6 (125 mg/kg, i,p,) 30 min prior to challenge with a dose of saline (green line) or GD (33 µg/kg, s.c.; red line) and treated one min later with atropine methyl nitrate (2 mg/kg for saline-exposed, 4 mg.kg for GD-exposed, i.p.). Animals were randomly assigned to one of the 4 treatment groups: AP (atropine sulfate + 2-PAM, 2A), MDZ (AP + midazolam, 2B), ENBA (AP + ENBA, 2C), and MDZ + ENBA (AP + midazolam + ENBA, 2D). Treatments were administrated i.p. at 15 min after GD-induced EEG seizure onset or relevant time after saline exposure groups. Heart rate (HR) was recorded hourly on experimental day for 5 h, at 24 h, and on day 7 and 14 following exposure. The percent change from baseline was calculated as: (100 x (24 h HR – baseline HR))/baseline HR for each animal before averaging. Across exposure analysis performed using Mann-Whitney test. (*) indicates datapoints where groups are significantly different (p≤0.05) between exposure groups. The negative impact of GD on HR was enhanced in the MDZ treatment group (2B). ENBA treatments (alone and in conjunction with MDZ) obscured this effect. (PNG 98 kb) [file 12640_2024_717_Fig12_ESM.png]

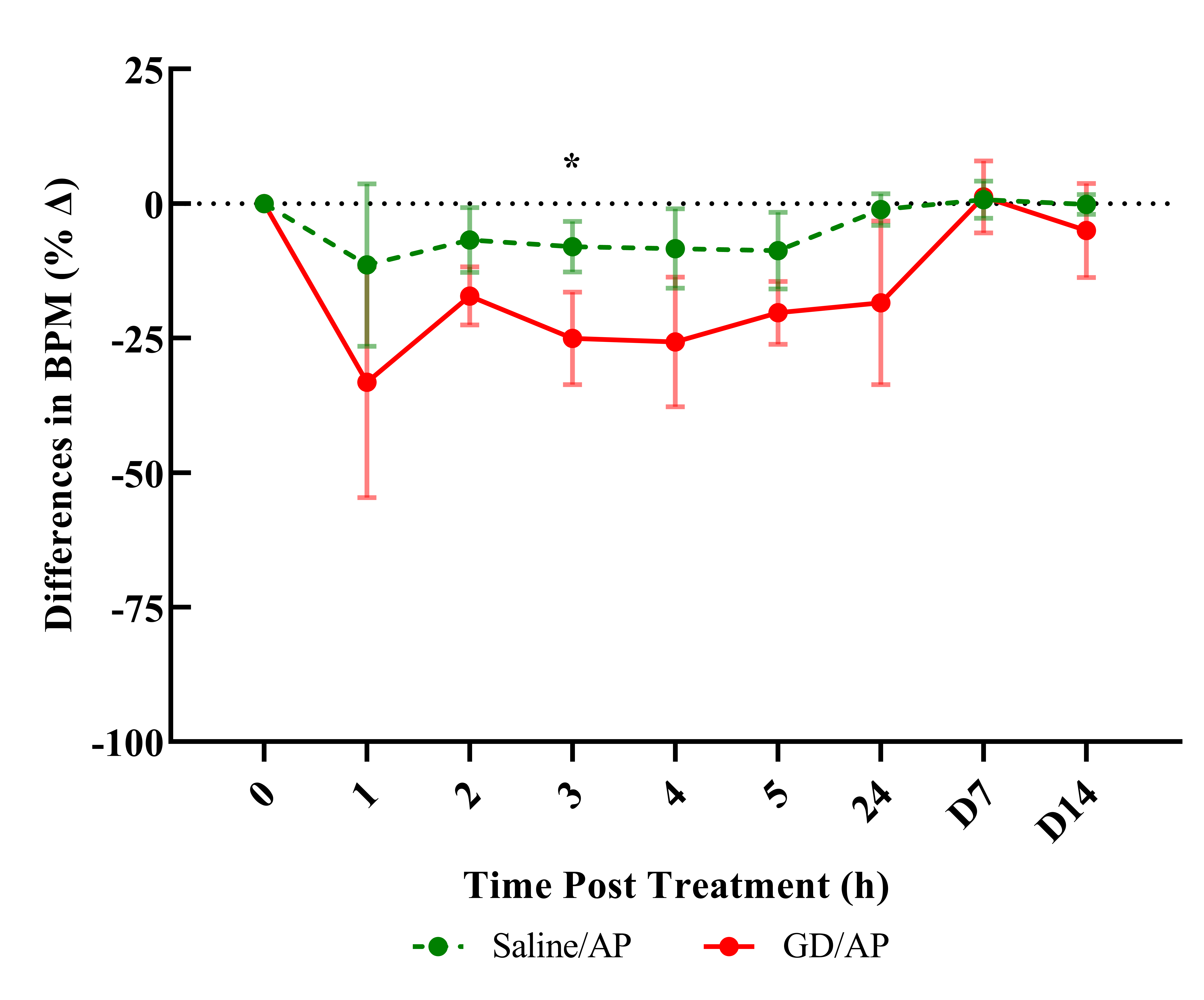

Supplement: Supplementary file 10 — High resolution image (TIF 1466 kb) [file 12640_2024_717_MOESM5_ESM.tif]

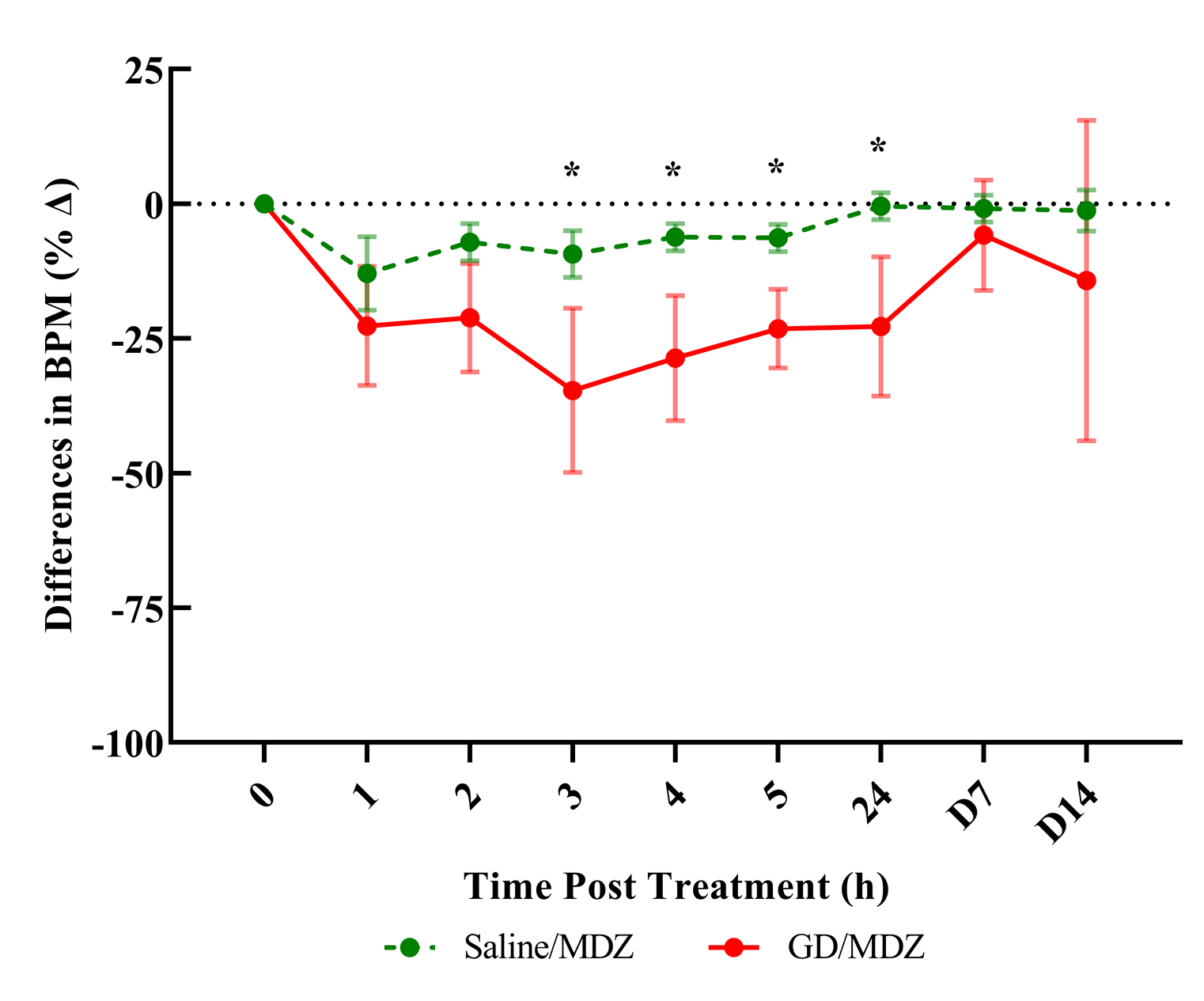

Supplement: Supplementary file 11 — Supplementary file6 (PNG 99 kb) [file 12640_2024_717_Fig13_ESM.png]

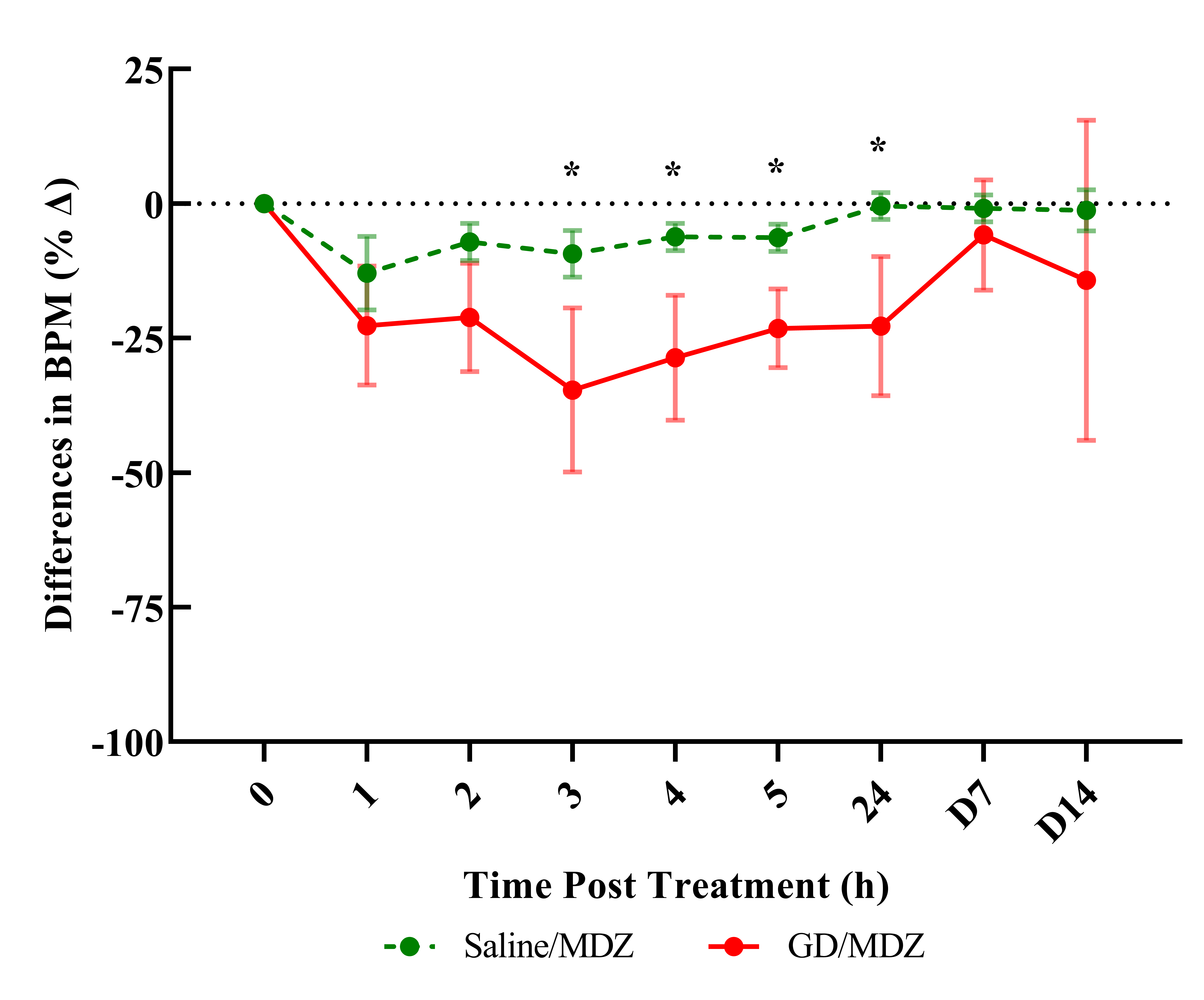

Supplement: Supplementary file 12 — High resolution image (TIF 1475 kb) [file 12640_2024_717_MOESM6_ESM.tif]

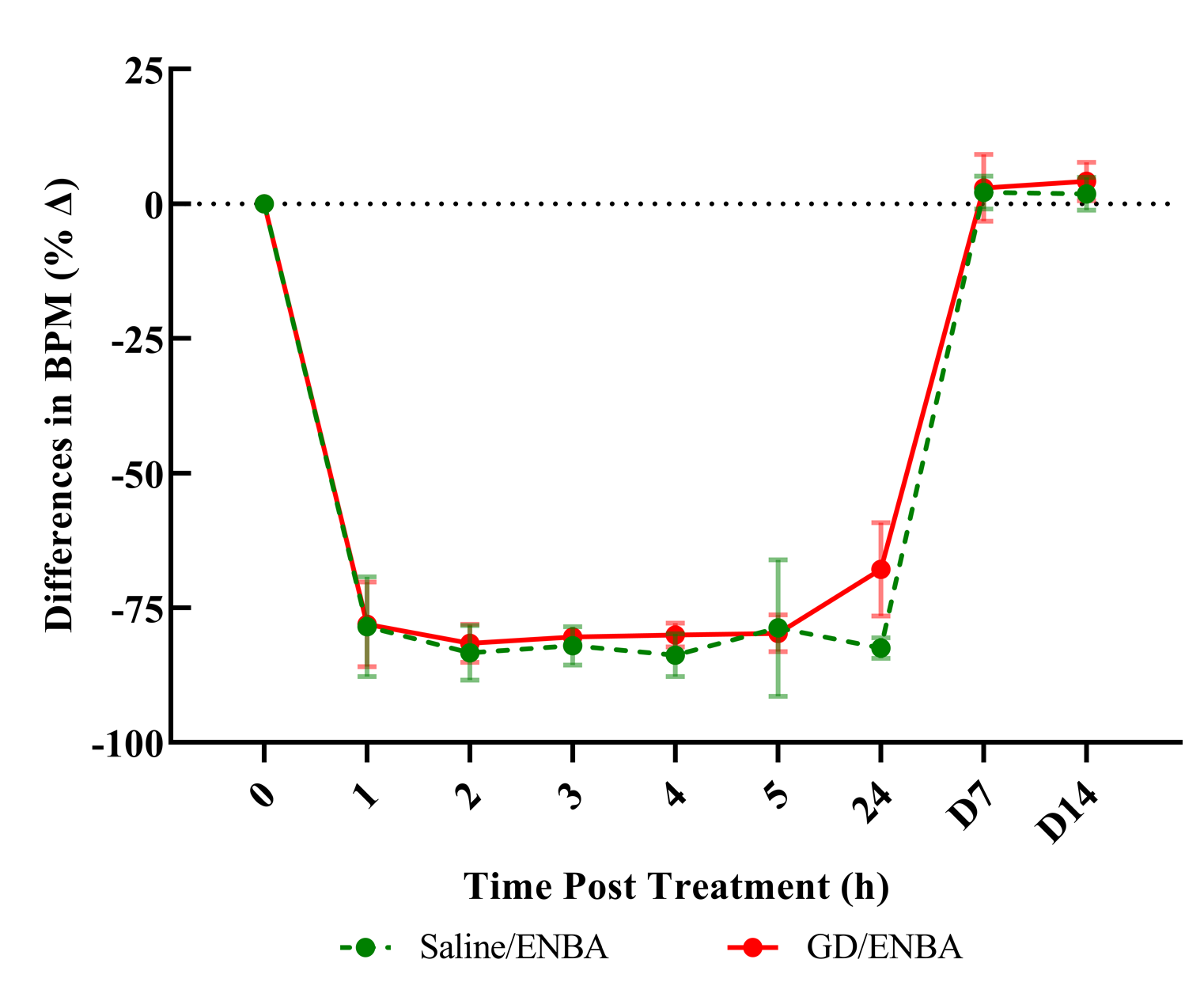

Supplement: Supplementary file 13 — Supplementary file7 (PNG 108 kb) [file 12640_2024_717_Fig14_ESM.png]

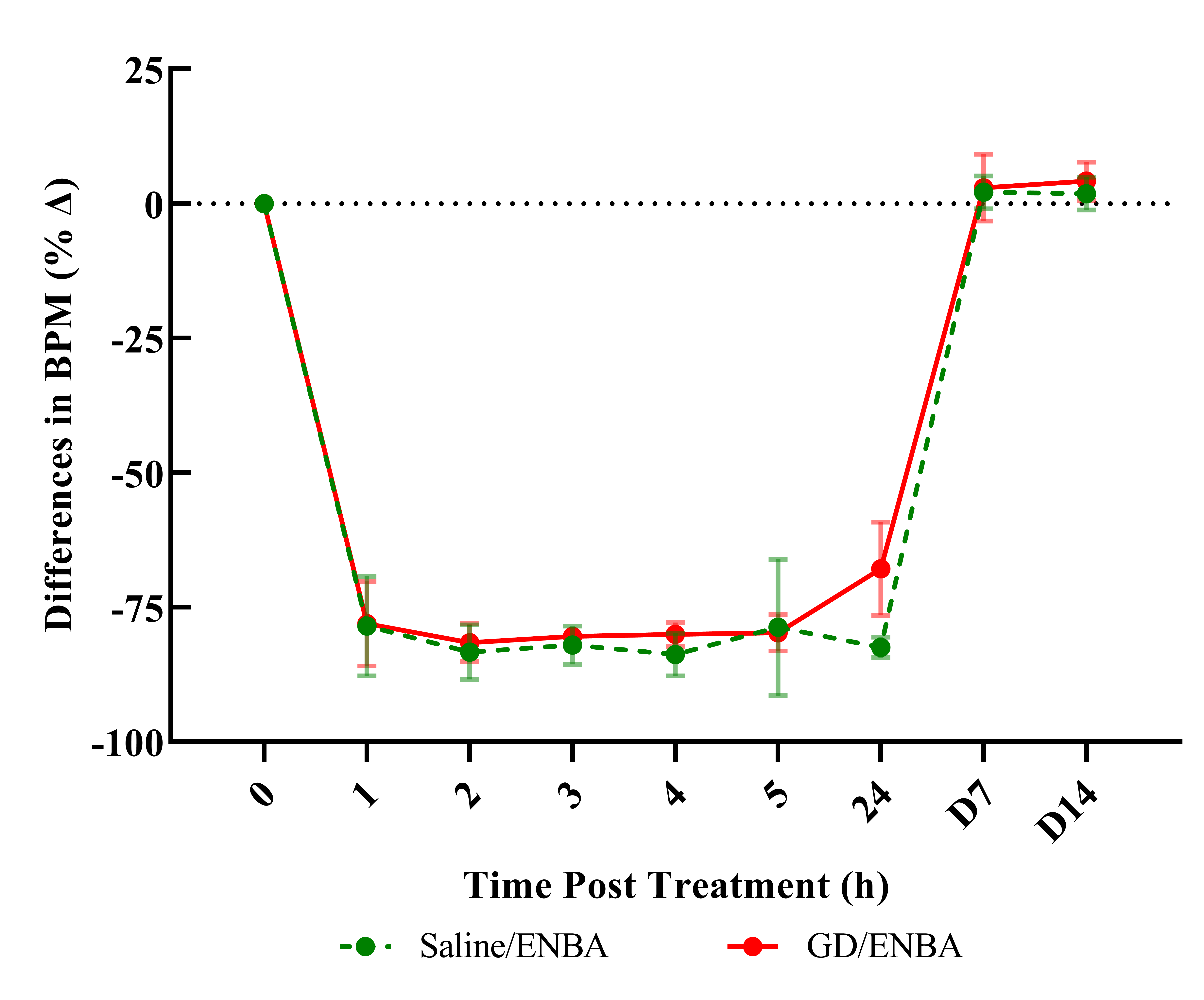

Supplement: Supplementary file 14 — High resolution image (TIF 1493 kb) [file 12640_2024_717_MOESM7_ESM.tif]

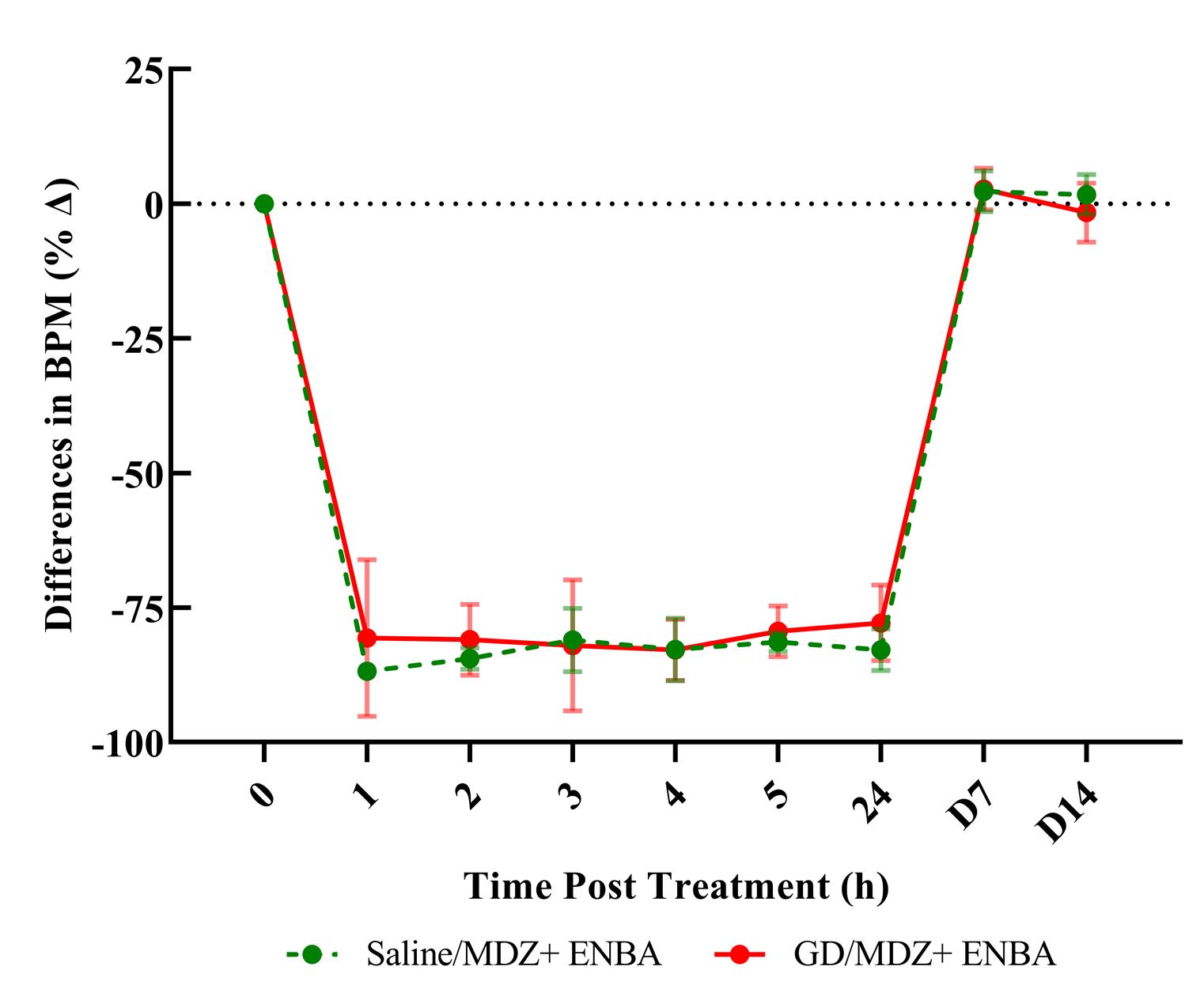

Supplement: Supplementary file 15 — Supplementary file8 (PNG 112 kb) [file 12640_2024_717_Fig15_ESM.png]

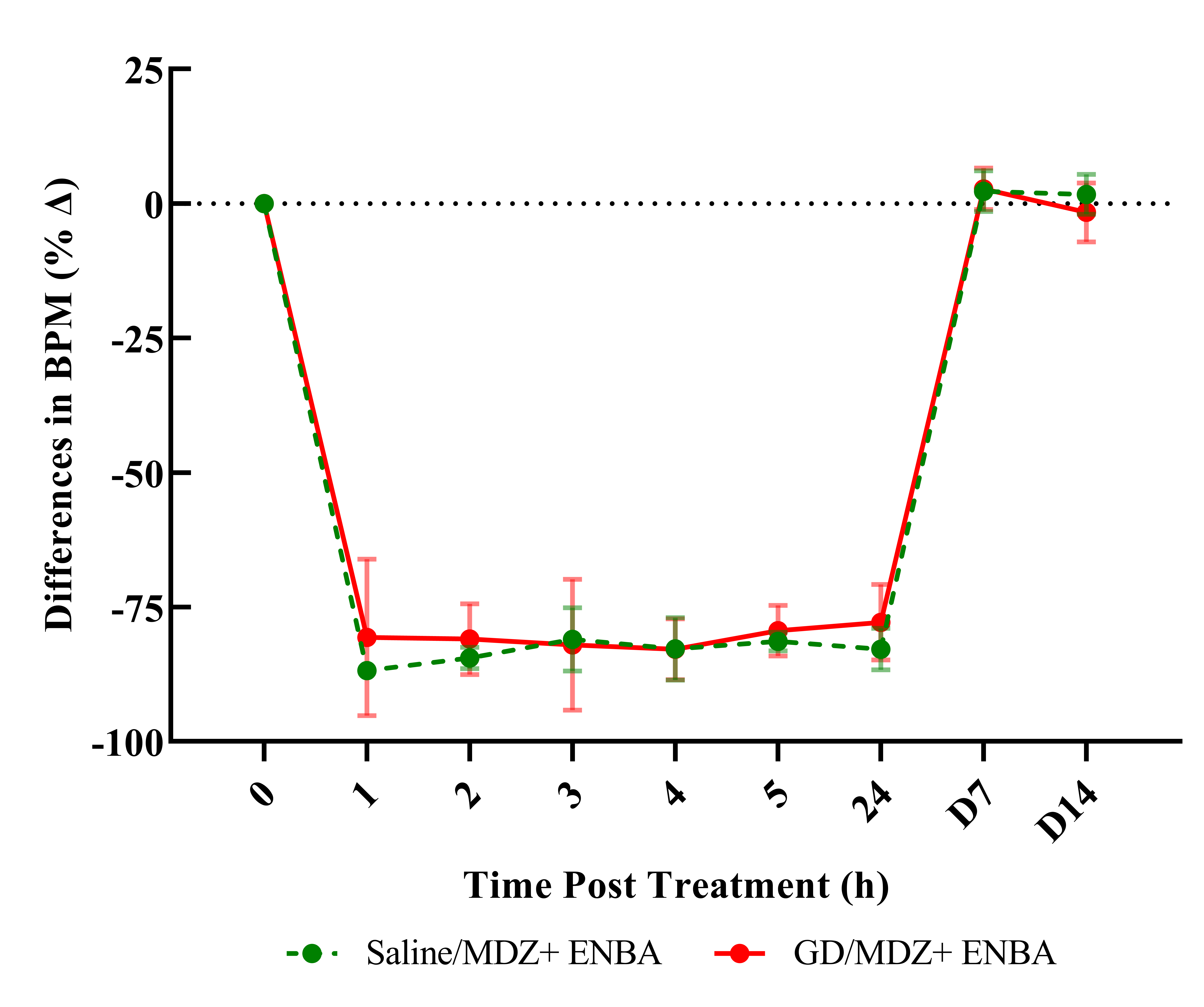

Supplement: Supplementary file 16 — High resolution image (TIF 1512 kb) [file 12640_2024_717_MOESM8_ESM.tif]
