# Supplementary material for: Pharmacology of Adenosine A1 Receptor Agonist in a Humanized Esterase Mouse Seizure Model Following Soman Intoxication
Source: Neurotox Res. 2024 Sep 4;42(5):41. doi: 10.1007/s12640-024-00717-z (PMC11374867; doi:10.1007/s12640-024-00717-z)
Supplement: Supplementary file 17 — Supplementary file9 (DOCX 73.5 KB) [file 12640_2024_717_MOESM9_ESM.docx]

**Supplementary Tables**

**Table 8.**  Pairwise comparisons on differences in wave power at the gamma (γ) frequency range.

1. Within Treatment. Zero is equivalent to baseline.

|  | AP | MDZ | ENBA | ENBA+MDZ |
| --- | --- | --- | --- | --- |
| 0 vs. 30 | *** | *** | *** |  |
| 0 vs. 60 | *** | *** | *** | *** |
| 0 vs. 90 | *** | *** | *** | *** |
| 0 vs. 120 | *** | ** | *** | *** |
| 0 vs. 150 | *** |  | *** | *** |
| 0 vs. 180 | *** |  | *** | *** |
| 0 vs. 210 | *** |  | *** | *** |
| 0 vs. 240 | ** |  | *** | *** |
| 0 vs. 270 | ** |  | *** | *** |
| 0 vs. 300 | * | * | *** | *** |
| * p<0.05, ** p<0.01, *** p<0.001 | | | | |

1. Across Pairs of Treatment Groups.

|  | 0 | 30 | 60 | 90 | 120 | 150 | 180 | 210 | 240 | 270 | 300 |
| --- | --- | --- | --- | --- | --- | --- | --- | --- | --- | --- | --- |
| AP vs. MDZ |  |  | * |  | * | *** | ** | * |  | * |  |
| AP vs. ENBA+MDZ |  | * | *** | *** | *** | *** | *** | *** | *** | *** | *** |
| AP vs. ENBA |  | *** | *** | *** | *** | *** | *** | *** | *** | *** | *** |
| * p<0.05, ** p<0.01, *** p<0.001 | | | | | | | | | | | |

**Table 9.**  Significant Differences in Spike Frequency

1. Within Treatment. Zero is equivalent to baseline.

|  | AP | MDZ | ENBA | ENBA+MDZ |
| --- | --- | --- | --- | --- |
| 0 vs. 30 | *** | *** | *** | *** |
| 0 vs. 60 | *** | *** | *** | *** |
| 0 vs. 90 | *** | ** | *** | *** |
| 0 vs. 120 | *** |  | *** | *** |
| 0 vs. 150 | *** |  | *** | *** |
| 0 vs. 180 | ** |  | *** | *** |
| 0 vs. 210 | ** |  | *** | *** |
| 0 vs. 240 | * |  | *** | *** |
| 0 vs. 270 | ** |  | *** | *** |
| 0 vs. 300 | ** |  | *** | *** |
| * p<0.05, ** p<0.01, *** p<0.001 | | | | |

1. Across Pairs of Treatment Groups.

|  | 0 | 30 | 60 | 90 | 120 | 150 | 180 | 210 | 240 | 270 | 300 |
| --- | --- | --- | --- | --- | --- | --- | --- | --- | --- | --- | --- |
| AP vs. MDZ |  |  |  |  | * | * |  |  |  |  |  |
| AP vs. ENBA+MDZ |  | *** | *** | *** | *** | *** | *** | *** | *** | *** | *** |
| AP vs. ENBA |  | *** | *** | *** | *** | *** | *** | *** | *** | *** | *** |
| * p<0.05, ** p<0.01, *** p<0.001 | | | | | | | | | | | |

**Table 10**. Pairwise comparisons in Temperature after Saline Exposure, post-exposure (PE) and after Treatment.

1. Within Treatments.

| Saline | AP | MDZ | ENBA | MDZ+ENBA |
| --- | --- | --- | --- | --- |
| Baseline vs. 0:04 PE |  |  |  |  |
| Baseline vs. 0:08 PE |  |  | * |  |
| Baseline vs. 0:15 PE |  |  |  |  |
| Baseline vs. 0:04 |  |  | * | * |
| Baseline vs. 0:08 |  |  | ** | ** |
| Baseline vs. 0:15 |  |  | *** | *** |
| Baseline vs. 0:30 |  |  | *** | *** |
| Baseline vs. 1:00 |  | * | ** | *** |
| Baseline vs. 2:00 |  | ** | *** | *** |
| Baseline vs. 3:00 |  | *** | *** | *** |
| Baseline vs. 4:00 |  | ** | *** | *** |
| Baseline vs. 5:00 |  |  | *** | *** |
| Baseline vs. 24:00 |  |  | *** | *** |
| Baseline vs. Day 7 |  |  |  |  |
| Baseline vs. Day 14 |  |  |  |  |
| * p<0.05, ** p<0.01, *** p<0.001 | | | | |

1. Across pairs of Treatments.

| Baseline |  |
| --- | --- |
| Saline/AP+15 min vs. Saline/MDZ + 15 min |  |
| Saline/AP+15 min vs. Saline/ENBA + 15 min |  |
| Saline/AP+15 min vs. Saline/MDZ+ ENBA + 15 min |  |
|  |  |
| 0:04 PE |  |
| Saline/AP+15 min vs. Saline/MDZ + 15 min |  |
| Saline/AP+15 min vs. Saline/ENBA + 15 min |  |
| Saline/AP+15 min vs. Saline/MDZ+ ENBA + 15 min |  |
|  |  |
| 0:08 PE |  |
| Saline/AP+15 min vs. Saline/MDZ + 15 min |  |
| Saline/AP+15 min vs. Saline/ENBA + 15 min |  |
| Saline/AP+15 min vs. Saline/MDZ+ ENBA + 15 min |  |
|  |  |
| 0:15 PE |  |
| Saline/AP+15 min vs. Saline/MDZ + 15 min |  |
| Saline/AP+15 min vs. Saline/ENBA + 15 min |  |
| Saline/AP+15 min vs. Saline/MDZ+ ENBA + 15 min |  |
|  |  |
| 0:04 |  |
| Saline/AP+15 min vs. Saline/MDZ + 15 min |  |
| Saline/AP+15 min vs. Saline/ENBA + 15 min | * |
| Saline/AP+15 min vs. Saline/MDZ+ ENBA + 15 min | ** |
|  |  |
| 0:08 |  |
| Saline/AP+15 min vs. Saline/MDZ + 15 min |  |
| Saline/AP+15 min vs. Saline/ENBA + 15 min | *** |
| Saline/AP+15 min vs. Saline/MDZ+ ENBA + 15 min | *** |
|  |  |
| 0:15 |  |
| Saline/AP+15 min vs. Saline/MDZ + 15 min | * |
| Saline/AP+15 min vs. Saline/ENBA + 15 min | *** |
| Saline/AP+15 min vs. Saline/MDZ+ ENBA + 15 min | *** |
|  |  |
| 0:30 |  |
| Saline/AP+15 min vs. Saline/MDZ + 15 min | * |
| Saline/AP+15 min vs. Saline/ENBA + 15 min | *** |
| Saline/AP+15 min vs. Saline/MDZ+ ENBA + 15 min | *** |
|  |  |
| 1:00 |  |
| Saline/AP+15 min vs. Saline/MDZ + 15 min |  |
| Saline/AP+15 min vs. Saline/ENBA + 15 min | ** |
| Saline/AP+15 min vs. Saline/MDZ+ ENBA + 15 min | *** |
|  |  |
| 2:00 |  |
| Saline/AP+15 min vs. Saline/MDZ + 15 min |  |
| Saline/AP+15 min vs. Saline/ENBA + 15 min | *** |
| Saline/AP+15 min vs. Saline/MDZ+ ENBA + 15 min | *** |
|  |  |
| 3:00 |  |
| Saline/AP+15 min vs. Saline/MDZ + 15 min | * |
| Saline/AP+15 min vs. Saline/ENBA + 15 min | *** |
| Saline/AP+15 min vs. Saline/MDZ+ ENBA + 15 min | *** |
|  |  |
| 4:00 |  |
| Saline/AP+15 min vs. Saline/MDZ + 15 min |  |
| Saline/AP+15 min vs. Saline/ENBA + 15 min | *** |
| Saline/AP+15 min vs. Saline/MDZ+ ENBA + 15 min | *** |
|  |  |
| 5:00 |  |
| Saline/AP+15 min vs. Saline/MDZ + 15 min |  |
| Saline/AP+15 min vs. Saline/ENBA + 15 min | *** |
| Saline/AP+15 min vs. Saline/MDZ+ ENBA + 15 min | *** |
|  |  |
| 24:00 |  |
| Saline/AP+15 min vs. Saline/MDZ + 15 min |  |
| Saline/AP+15 min vs. Saline/ENBA + 15 min | *** |
| Saline/AP+15 min vs. Saline/MDZ+ ENBA + 15 min | *** |
|  |  |
| Day 7 |  |
| Saline/AP+15 min vs. Saline/MDZ + 15 min |  |
| Saline/AP+15 min vs. Saline/ENBA + 15 min |  |
| Saline/AP+15 min vs. Saline/MDZ+ ENBA + 15 min |  |
|  |  |
| Day 14 |  |
| Saline/AP+15 min vs. Saline/MDZ + 15 min |  |
| Saline/AP+15 min vs. Saline/ENBA + 15 min |  |
| Saline/AP+15 min vs. Saline/MDZ+ ENBA + 15 min |  |
| * p<0.05, ** p<0.01, *** p<0.001 |  |

**Table 11**. Pairwise comparisons in Temperature after Soman Exposure, post-exposure (PE) and after Treatment.

1. Within Treatments.

| GD | AP | MDZ | ENBA | MDZ+ENBA |
| --- | --- | --- | --- | --- |
| Baseline vs. 0:04 PE | * | *** | *** | *** |
| Baseline vs. 0:08 PE | ** | *** | *** | *** |
| Baseline vs. 0:15 PE | *** | *** | *** | *** |
| Baseline vs. 0:04 | ** | *** | *** | *** |
| Baseline vs. 0:08 | ** | *** | *** | *** |
| Baseline vs. 0:15 | ** | *** | *** | *** |
| Baseline vs. 0:30 | * | *** | *** | *** |
| Baseline vs. 1:00 |  | ** | *** | *** |
| Baseline vs. 2:00 | ** | *** | *** | *** |
| Baseline vs. 3:00 | *** | *** | *** | *** |
| Baseline vs. 4:00 | *** | ** | *** | *** |
| Baseline vs. 5:00 | *** | *** | *** | *** |
| Baseline vs. 24:00 |  | * | *** | *** |
| Baseline vs. Day 7 |  |  |  |  |
| Baseline vs. Day 14 |  |  |  | * |
| * p<0.05, ** p<0.01, *** p<0.001 | | | | |

1. Across pairs of treatments.

| Baseline |  |
| --- | --- |
| GD/AP+15 min vs. GD/MDZ + 15 min |  |
| GD/AP+15 min vs. GD/ENBA + 15 min |  |
| GD/AP+15 min vs. GD/MDZ+ ENBA + 15 min |  |
|  |  |
| 0:04 PE |  |
| GD/AP+15 min vs. GD/MDZ + 15 min |  |
| GD/AP+15 min vs. GD/ENBA + 15 min |  |
| GD/AP+15 min vs. GD/MDZ+ ENBA + 15 min |  |
|  |  |
| 0:08 PE |  |
| GD/AP+15 min vs. GD/MDZ + 15 min |  |
| GD/AP+15 min vs. GD/ENBA + 15 min |  |
| GD/AP+15 min vs. GD/MDZ+ ENBA + 15 min |  |
|  |  |
| 0:15 PE |  |
| GD/AP+15 min vs. GD/MDZ + 15 min |  |
| GD/AP+15 min vs. GD/ENBA + 15 min |  |
| GD/AP+15 min vs. GD/MDZ+ ENBA + 15 min |  |
|  |  |
| 0:04 |  |
| GD/AP+15 min vs. GD/MDZ + 15 min |  |
| GD/AP+15 min vs. GD/ENBA + 15 min | * |
| GD/AP+15 min vs. GD/MDZ+ ENBA + 15 min |  |
|  |  |
| 0:08 |  |
| GD/AP+15 min vs. GD/MDZ + 15 min |  |
| GD/AP+15 min vs. GD/ENBA + 15 min | ** |
| GD/AP+15 min vs. GD/MDZ+ ENBA + 15 min |  |
|  |  |
| 0:15 |  |
| GD/AP+15 min vs. GD/MDZ + 15 min |  |
| GD/AP+15 min vs. GD/ENBA + 15 min | *** |
| GD/AP+15 min vs. GD/MDZ+ ENBA + 15 min | *** |
|  |  |
| 0:30 |  |
| GD/AP+15 min vs. GD/MDZ + 15 min |  |
| GD/AP+15 min vs. GD/ENBA + 15 min | *** |
| GD/AP+15 min vs. GD/MDZ+ ENBA + 15 min | *** |
|  |  |
| 1:00 |  |
| GD/AP+15 min vs. GD/MDZ + 15 min |  |
| GD/AP+15 min vs. GD/ENBA + 15 min | *** |
| GD/AP+15 min vs. GD/MDZ+ ENBA + 15 min | *** |
|  |  |
| 2:00 |  |
| GD/AP+15 min vs. GD/MDZ + 15 min |  |
| GD/AP+15 min vs. GD/ENBA + 15 min | *** |
| GD/AP+15 min vs. GD/MDZ+ ENBA + 15 min | *** |
|  |  |
| 3:00 |  |
| GD/AP+15 min vs. GD/MDZ + 15 min |  |
| GD/AP+15 min vs. GD/ENBA + 15 min | *** |
| GD/AP+15 min vs. GD/MDZ+ ENBA + 15 min | *** |
|  |  |
| 4:00 |  |
| GD/AP+15 min vs. GD/MDZ + 15 min |  |
| GD/AP+15 min vs. GD/ENBA + 15 min | *** |
| GD/AP+15 min vs. GD/MDZ+ ENBA + 15 min | *** |
|  |  |
| 5:00 |  |
| GD/AP+15 min vs. GD/MDZ + 15 min |  |
| GD/AP+15 min vs. GD/ENBA + 15 min | *** |
| GD/AP+15 min vs. GD/MDZ+ ENBA + 15 min | *** |
|  |  |
| 24:00 |  |
| GD/AP+15 min vs. GD/MDZ + 15 min |  |
| GD/AP+15 min vs. GD/ENBA + 15 min | *** |
| GD/AP+15 min vs. GD/MDZ+ ENBA + 15 min | *** |
|  |  |
| Day 7 |  |
| GD/AP+15 min vs. GD/MDZ + 15 min |  |
| GD/AP+15 min vs. GD/ENBA + 15 min |  |
| GD/AP+15 min vs. GD/MDZ+ ENBA + 15 min |  |
|  |  |
| Day 14 |  |
| GD/AP+15 min vs. GD/MDZ + 15 min |  |
| GD/AP+15 min vs. GD/ENBA + 15 min |  |
| GD/AP+15 min vs. GD/MDZ+ ENBA + 15 min |  |
| * p<0.05, ** p<0.01, *** p<0.001 |  |

**Table 12**. Pairwise comparisons in Temperature between Saline and Soman Exposure, post-exposure (PE) and after Treatment.

|  | AP: Saline vs. GD | | MDZ: Saline/vs. GD | | ENBA: Saline vs. GD | | MDZ+ENBA: Saline vs. GD | |
| --- | --- | --- | --- | --- | --- | --- | --- | --- |
|  | Mann-Whitney U | q-value | Mann-Whitney U | q-value | Mann-Whitney U | q-value | Mann-Whitney U | q-value |
| Baseline |  |  |  |  |  |  |  |  |
| 0:04 PE |  |  | 14 | 0.006094 | 2 | 0.000323 |  |  |
| 0:08 PE | 6.5 | 0.001546 | 2 | 0.00016 | 0 | 0.000154 | 0 | 0.0006 |
| 0:15 PE | 0.5 | 0.000162 | 0.5 | 0.000092 | 0 | 0.000154 | 4.5 | 0.004301 |
|  | |  |  |  |  |  |  |  |
| 0:04 | 2 | 0.000215 | 0 | 0.000092 | 0 | 0.000154 | 5.5 | 0.004301 |
| 0:08 | 1 | 0.000162 | 0 | 0.000092 | 0.5 | 0.000323 |  |  |
| 0:15 | 7 | 0.001656 | 3.5 | 0.000241 | 6 | 0.002116 |  |  |
| 0:30 | 8.5 | 0.002567 |  |  |  |  |  |  |
| 1:00 |  |  |  |  | 10 | 0.008177 |  |  |
| 2:00 |  |  | 9 | 0.001395 |  |  |  |  |
| 3:00 | 0 | 0.000162 | 4 | 0.000285 |  |  |  |  |
| 4:00 | 0 | 0.000162 | 5.5 | 0.000391 |  |  |  |  |
| 5:00 | 2 | 0.000215 | 6 | 0.001121 |  |  |  |  |
| 24:00 |  |  | 2 | 0.000285 |  |  |  |  |
| Day 7 |  |  |  |  |  |  |  |  |
| Day 14 |  |  |  |  |  |  |  |  |

**Table 13.** Heart Rate Means across time by Exposure and Treatment

1. Saline-Exposed

| Time | Saline/AP | | | Saline/MDZ | | | Saline/ENBA | | | Saline/MDZ+ ENBA | | |
| --- | --- | --- | --- | --- | --- | --- | --- | --- | --- | --- | --- | --- |
| (Day) | Mean | SD | N | Mean | SD | N | Mean | SD | N | Mean | SD | N |
| 0 | 760 | 22 | 8 | 760 | 14 | 8 | 755 | 31 | 8 | 737 | 19 | 8 |
| 1 | 671 | 131 | 8 | 661 | 58 | 8 | 162 | 80 | 8 | 98 | 6 | 7 |
| 2 | 708 | 56 | 8 | 706 | 32 | 8 | 124 | 25 | 4 | 115 | 17 | 7 |
| 3 | 695 | 33 | 7 | 689 | 40 | 8 | 139 | 14 | 4 | 138 | 13 | 3 |
| 4 | 691 | 46 | 6 | 713 | 24 | 8 | 124 | 23 | 5 | 129 | 19 | 3 |
| 5 | 692 | 59 | 8 | 712 | 17 | 8 | 160 | 85 | 6 | 137 | 13 | 6 |
| 24 | 751 | 28 | 8 | 757 | 24 | 8 | 132 | 17 | 8 | 126 | 33 | 8 |
| D7 | 765 | 21 | 8 | 754 | 23 | 8 | 770 | 10 | 8 | 753 | 23 | 8 |
| D14 | 759 | 24 | 8 | 750 | 25 | 8 | 769 | 30 | 8 | 749 | 25 | 8 |

1. Soman-Exposed

| Time | GD/AP | | | GD/MDZ | | | GD/ENBA | | | GD/MDZ+ ENBA | | |
| --- | --- | --- | --- | --- | --- | --- | --- | --- | --- | --- | --- | --- |
| (Day) | Mean | SD | N | Mean | SD | N | Mean | SD | N | Mean | SD | N |
| 0 | 758 | 40 | 10 | 750 | 26 | 10 | 729 | 41 | 10 | 770 | 34 | 10 |
| 1 | 508 | 236 | 10 | 582 | 129 | 10 | 160 | 72 | 8 | 148 | 134 | 8 |
| 2 | 628 | 68 | 10 | 590 | 96 | 8 | 137 | 32 | 8 | 142 | 26 | 4 |
| 3 | 566 | 82 | 10 | 528 | 89 | 7 | 143 | 15 | 9 | 142 | 38 | 3 |
| 4 | 562 | 129 | 10 | 522 | 103 | 8 | 145 | 23 | 10 | 135 | 35 | 5 |
| 5 | 602 | 47 | 10 | 566 | 69 | 10 | 146 | 25 | 9 | 160 | 42 | 8 |
| 24 | 612 | 95 | 7 | 568 | 128 | 8 | 234 | 83 | 9 | 171 | 71 | 9 |
| D7 | 765 | 39 | 7 | 701 | 76 | 7 | 760 | 41 | 8 | 789 | 32 | 9 |
| D14 | 722 | 99 | 7 | 634 | 166 | 5 | 770 | 21 | 8 | 755 | 51 | 9 |

**Table 14.** Pairwise comparisons in % change of Heart Rate after Saline Exposure and Treatment.

1. Within treatment. Zero is equivalent to baseline.

| Saline | AP | MDZ | ENBA | MDZ+ENBA |
| --- | --- | --- | --- | --- |
| 0 vs. 1 |  | * | *** | *** |
| 0 vs. 2 |  | ** | *** | *** |
| 0 vs. 3 | * | ** | *** | *** |
| 0 vs. 4 |  | ** | *** | *** |
| 0 vs. 5 |  | ** | *** | *** |
| 0 vs. 24 |  |  | *** | *** |
| 0 vs. D7 |  |  |  |  |
| 0 vs. D14 |  |  |  |  |
| * p<0.05, ** p<0.01, *** p<0.001 | | | | |

1. Across pairs of Treatment groups.

| 0 |  |
| --- | --- |
| Saline/AP vs. Saline/MDZ |  |
| Saline/AP vs. Saline/ENBA |  |
| Saline/AP vs. Saline/MDZ+ ENBA |  |
|  |  |
| 1 |  |
| Saline/AP vs. Saline/MDZ |  |
| Saline/AP vs. Saline/ENBA | *** |
| Saline/AP vs. Saline/MDZ+ ENBA | *** |
|  |  |
| 2 |  |
| Saline/AP vs. Saline/MDZ |  |
| Saline/AP vs. Saline/ENBA | *** |
| Saline/AP vs. Saline/MDZ+ ENBA | *** |
|  |  |
| 3 |  |
| Saline/AP vs. Saline/MDZ |  |
| Saline/AP vs. Saline/ENBA | *** |
| Saline/AP vs. Saline/MDZ+ ENBA | *** |
|  |  |
| 4 |  |
| Saline/AP vs. Saline/MDZ |  |
| Saline/AP vs. Saline/ENBA | *** |
| Saline/AP vs. Saline/MDZ+ ENBA | *** |
|  |  |
| 5 |  |
| Saline/AP vs. Saline/MDZ |  |
| Saline/AP vs. Saline/ENBA | *** |
| Saline/AP vs. Saline/MDZ+ ENBA | *** |
|  |  |
| 24 |  |
| Saline/AP vs. Saline/MDZ |  |
| Saline/AP vs. Saline/ENBA | *** |
| Saline/AP vs. Saline/MDZ+ ENBA | *** |
|  |  |
| D7 |  |
| Saline/AP vs. Saline/MDZ |  |
| Saline/AP vs. Saline/ENBA |  |
| Saline/AP vs. Saline/MDZ+ ENBA |  |
|  |  |
| D14 |  |
| Saline/AP vs. Saline/MDZ |  |
| Saline/AP vs. Saline/ENBA |  |
| Saline/AP vs. Saline/MDZ+ ENBA |  |
| * p<0.05, ** p<0.01, *** p<0.001 |  |

**Table 15.** Pairwise comparisons in % change of Heart Rate after Soman Exposure and Treatment.

1. Within treatment. Zero is equivalent to baseline.

| GD | AP | MDZ | ENBA | MDZ+ENBA |
| --- | --- | --- | --- | --- |
| 0 vs. 1 | * | * | *** | *** |
| 0 vs. 2 | *** | * | *** | *** |
| 0 vs. 3 | *** | ** | *** | ** |
| 0 vs. 4 | ** | ** | *** | *** |
| 0 vs. 5 | *** | *** | *** | *** |
| 0 vs. 24 |  | * | *** | *** |
| 0 vs. D7 |  |  |  |  |
| 0 vs. D14 |  |  |  |  |
| * p<0.05, ** p<0.01, *** p<0.001 | | | | |

1. Across pairs of Treatment groups.

| 0 |  |
| --- | --- |
| GD/AP vs. GD/MDZ |  |
| GD/AP vs. GD/ENBA |  |
| GD/AP vs. GD/MDZ+ ENBA |  |
|  |  |
| 1 |  |
| GD/AP vs. GD/MDZ |  |
| GD/AP vs. GD/ENBA | ** |
| GD/AP vs. GD/MDZ+ ENBA | ** |
|  |  |
| 2 |  |
| GD/AP vs. GD/MDZ |  |
| GD/AP vs. GD/ENBA | *** |
| GD/AP vs. GD/MDZ+ ENBA | *** |
|  |  |
| 3 |  |
| GD/AP vs. GD/MDZ |  |
| GD/AP vs. GD/ENBA | *** |
| GD/AP vs. GD/MDZ+ ENBA | *** |
|  |  |
| 4 |  |
| GD/AP vs. GD/MDZ |  |
| GD/AP vs. GD/ENBA | *** |
| GD/AP vs. GD/MDZ+ ENBA | *** |
|  |  |
| 5 |  |
| GD/AP vs. GD/MDZ |  |
| GD/AP vs. GD/ENBA | *** |
| GD/AP vs. GD/MDZ+ ENBA | *** |
|  |  |
| 24 |  |
| GD/AP vs. GD/MDZ |  |
| GD/AP vs. GD/ENBA | *** |
| GD/AP vs. GD/MDZ+ ENBA | *** |
|  |  |
| D7 |  |
| GD/AP vs. GD/MDZ |  |
| GD/AP vs. GD/ENBA |  |
| GD/AP vs. GD/MDZ+ ENBA |  |
|  |  |
| D14 |  |
| GD/AP vs. GD/MDZ |  |
| GD/AP vs. GD/ENBA |  |
| GD/AP vs. GD/MDZ+ ENBA |  |
| * p<0.05, ** p<0.01, *** p<0.001 |  |

**Table 16.** Pairwise comparisons in % change of Heart Rate between Saline and Soman (GD) Exposure and Treatment.

|  | AP: Saline vs. GD |  | MDZ: Saline/vs. GD |  | ENBA: Saline vs. GD | | MDZ+ENBA: Saline vs. GD |  |
| --- | --- | --- | --- | --- | --- | --- | --- | --- |
|  | Mann-Whitney U | q-value | Mann-Whitney U | q-value | Mann-Whitney U | q-value | Mann-Whitney U | q-value |
| Baseline |  |  |  |  |  |  |  |  |
| 1:00 |  |  |  |  |  |  |  |  |
| 2:00 |  |  |  |  |  |  |  |  |
| 3:00 | 2 | 0.00332 | 4 | 0.002354 |  |  |  |  |
| 4:00 |  |  | 2 | 0.000554 |  |  |  |  |
| 5:00 |  |  | 4 | 0.000554 |  |  |  |  |
| 24:00 |  |  | 0 | 0.000415 |  |  |  |  |
| Day 7 |  |  |  |  |  |  |  |  |
| Day 14 |  |  |  |  |  |  |  |  |

**Table 17**. Weight Means at Baseline by Exposure and Treatment.

1. Saline-Exposed

|  | Saline/AP | | | Saline/MDZ | | | Saline/ENBA | | | Saline/MDZ+ ENBA | | |
| --- | --- | --- | --- | --- | --- | --- | --- | --- | --- | --- | --- | --- |
| Day | AVG | SD | N | AVG | SD | N | AVG | SD | N | AVG | SD | N |
| 0 | 26.3 | 1.2 | 8 | 29.0 | 2.9 | 8 | 27.4 | 2.3 | 8 | 29.7 | 1.4 | 8 |

1. Soman-Exposed

|  | GD/AP | | | GD/MDZ | | | GD/ENBA | | | GD/MDZ+ ENBA | | |
| --- | --- | --- | --- | --- | --- | --- | --- | --- | --- | --- | --- | --- |
| Day | AVG | SD | N | AVG | SD | N | AVG | SD | N | AVG | SD | N |
| 0 | 26.9 | 1.4 | 10 | 27.6 | 1.6 | 10 | 27.9 | 1.7 | 10.0 | 27.1 | 1.6 | 10 |

**Table 18**. Weight Percent Change from Baseline by Exposure and Treatment.

1. Saline-Exposed

|  | Saline/AP | | | Saline/MDZ | | | Saline/ENBA | | | Saline/MDZ+ ENBA | | |
| --- | --- | --- | --- | --- | --- | --- | --- | --- | --- | --- | --- | --- |
| Day | AVG | SD | N | AVG | SD | N | AVG | SD | N | AVG | SD | N |
| 1 | -7.3 | 5.2 | 8 | -4.5 | 3.2 | 8 | -4.9 | 1.9 | 8 | -1.0 | 1.8 | 8 |
| 2 | 0.4 | 1.1 | 3 | -1.1 | 2.4 | 7 | -7.6 | 3.0 | 7 | -7.7 | 3.0 | 8 |
| 3 | 1.9 | 0.0 | 1 | -1.6 | 2.9 | 3 | -7.5 | 3.4 | 3 | -8.8 | 4.0 | 8 |
| 4 | 1.9 | 3.2 | 6 | -1.9 | 0.0 | 1 | -4.0 | 0.0 | 1 | --- | --- | **---** |
| 5 | 1.3 | 3.2 | 7 | 0.7 | 2.5 | 5 | -2.2 | 2.5 | 5 | --- | --- | **---** |
| 6 | 0.7 | 4.5 | 7 | 0.3 | 3.3 | 8 | -0.7 | 2.8 | 8 | -3.5 | 2.6 | 8 |
| 7 | 2.1 | 2.2 | 8 | 0.2 | 3.4 | 8 | -1.1 | 2.1 | 8 | -4.2 | 2.5 | 8 |
| 8 | 1.8 | 2.6 | 8 | 2.2 | 3.2 | 8 | -0.7 | 2.1 | 8 | -4.6 | 2.8 | 8 |
| 9 | 2.2 | 2.2 | 3 | 1.7 | 3.6 | 7 | -0.7 | 1.1 | 7 | -3.3 | 2.4 | 8 |
| 10 | 4.8 | 0.0 | 1 | 1.9 | 7.2 | 3 | -0.2 | 1.4 | 3 | -3.5 | 3.7 | 8 |
| 11 | 1.5 | 6.1 | 6 | -1.6 | 0.0 | 1 | -1.0 | 0.0 | 1 | --- | --- | **---** |
| 12 | -0.2 | 5.0 | 7 | -0.1 | 2.8 | 5 | -0.5 | 1.6 | 5 | --- | --- | **---** |
| 13 | 1.1 | 4.8 | 7 | 0.9 | 3.5 | 8 | -1.1 | 2.0 | 8 | -3.5 | 3.1 | 8 |
| 14 | 0.4 | 4.8 | 8 | 0.0 | 4.4 | 8 | -1.1 | 2.2 | 8 | -4.4 | 3.1 | 8 |

1. Soman-Exposed

|  | GD/AP | | | GD/MDZ | | | GD/ENBA | | | GD/MDZ+ ENBA | | |
| --- | --- | --- | --- | --- | --- | --- | --- | --- | --- | --- | --- | --- |
| Day | AVG | SD | N | AVG | SD | N | AVG | SD | N | AVG | SD | N |
| 1 | -9.4 | 4.6 | 5 | -10.3 | 4.1 | 7 | -9.0 | 5.5 | 8 | -4.9 | 3.2 | 9 |
| 2 | -16.3 | 3.8 | 4 | -14.9 | 3.7 | 6 | -22.1 | 0.0 | 1 | -11.3 | 1.4 | 3 |
| 3 | -12.7 | 5.6 | 4 | -13.3 | 5.1 | 5 | -27.1 | 0.0 | 1 | -6.4 | 0.0 | 2 |
| 4 |  |  |  | -15.6 | 0.0 | 1 | -18.3 | 5.7 | 6 | -4.7 | 7.4 | 5 |
| 5 | -7.2 | 0.0 | 1 | -9.8 | 3.6 | 2 | -13.5 | 3.4 | 6 | -1.9 | 6.0 | 6 |
| 6 | -11.7 | 4.9 | 2 | -7.5 | 8.0 | 3 | -15.2 | 4.4 | 7 | -2.0 | 4.1 | 8 |
| 7 | -11.4 | 7.8 | 5 | -10.9 | 8.8 | 7 | -14.5 | 4.8 | 7 | -1.9 | 4.7 | 8 |
| 8 | -7.1 | 7.4 | 5 | -7.3 | 9.6 | 7 | -10.6 | 5.6 | 6 | -2.4 | 4.0 | 8 |
| 9 | -4.9 | 9.1 | 4 | -5.4 | 9.5 | 6 | --- | --- | **---** | -3.3 | 4.2 | 3 |
| 10 | -5.6 | 9.2 | 4 | -7.1 | 8.7 | 5 | --- | --- | **---** | -2.2 | 1.5 | 2 |
| 11 | -4.8 | 0.0 | 1 | -9.1 | 6.0 | 2 | -22.8 | 9.9 | 6 | 0.6 | 3.8 | 5 |
| 12 | -7.6 | 0.0 | 1 | -2.1 | 7.7 | 2 | -18.4 | 7.1 | 6 | -0.8 | 3.5 | 6 |
| 13 | -1.0 | 5.3 | 4 | -0.7 | 4.6 | 5 | -23.9 | 11.1 | 6 | -0.4 | 3.9 | 8 |
| 14 | -7.0 | 14.4 | 5 | -4.9 | 12.3 | 7 | -21.7 | 13.9 | 5 | -0.3 | 3.8 | 8 |

**Table 19**. Pairwise comparisons in % Weight Change after Saline and Treatment.

1. Within treatment.

| Saline | AP | MDZ | ENBA | MDZ+ENBA |
| --- | --- | --- | --- | --- |
| 1 DAY PT vs. 7 DAY PT | *** | ** | ** | * |
| 1 DAY PT vs. 14 DAY PT | *** | ** | ** | * |
| * p<0.05, ** p<0.01, *** p<0.001 | | | | |

1. Across treatment.

|  | 1 DAY PT | 7 DAY PT | 14 DAY PT |
| --- | --- | --- | --- |
| Saline/AP vs. Saline/MDZ |  |  |  |
| Saline/AP vs. Saline/ENBA |  |  |  |
| Saline/AP vs. Saline/MDZ+ ENBA | ****** | ** | * |
| Saline/MDZ vs. Saline/ENBA |  |  |  |
| Saline/MDZ vs. Saline/MDZ+ ENBA |  | * | * |
| Saline/ENBA vs. Saline/MDZ+ ENBA |  |  |  |
| * p<0.05, ** p<0.01, *** p<0.001 | | | |

**Table 20**. Pairwise comparisons in % Weight Change after Soman and Treatment.

1. Within treatment.

| GD | AP | MDZ | ENBA | MDZ+ENBA |
| --- | --- | --- | --- | --- |
| 1 DAY PT vs. 7 DAY PT |  |  |  |  |
| 1 DAY PT vs. 14 DAY PT |  | *** |  |  |
| * p<0.05, ** p<0.01, *** p<0.001 | | | | |

1. Across Pairs of Treatment Groups.

|  | 1 DAY PT | 7 DAY PT | 14 DAY PT |
| --- | --- | --- | --- |
| GD/AP vs. GD/MDZ |  |  | *** |
| GD/AP vs. GD/ENBA |  | * |  |
| GD/AP vs. GD/MDZ+ ENBA | * | * |  |
| GD/MDZ vs. GD/ENBA |  | ** | *** |
| GD/MDZ vs. GD/MDZ+ ENBA | * | *** | *** |
| GD/ENBA vs. GD/MDZ+ ENBA |  |  |  |
| * p<0.05, ** p<0.01, *** p<0.001 | | | |

**Table 21**. Pairwise comparisons in % Weight Change between Saline and Soman (GD) Exposure and Treatment.

|  | AP: Saline vs. GD |  | MDZ: Saline/vs. GD | | ENBA: Saline vs. GD | | MDZ+ENBA: Saline vs. GD | |
| --- | --- | --- | --- | --- | --- | --- | --- | --- |
|  | Mann-Whitney U | q-value | Mann-Whitney U | q-value | Mann-Whitney U | q-value | Mann-Whitney U | q-value |
| Day 1 |  |  |  |  |  |  |  |  |
| Day 7 |  |  | 0 | 0.000628 |  |  |  |  |
| Day 14 |  |  |  |  |  |  |  |  |

**Table 22**.

| Degree of Toxicity: |  | Temperature (°C) | Weight (% of baseline) |
| --- | --- | --- | --- |
| non-toxic | 0 | 36.0+ | 96 |
| minimal toxic effect | 1 | 35.9-35.6 | 95 |
| Median toxic effect | 2 | 35.5-35.0 | 94 |
| Gross toxic effect | 3 | 34.9 | 93 |
